# Genomic characterization of Aeromonas spp. isolates from striped catfish with motile Aeromonas septicemia and human bloodstream infections in Vietnam

**DOI:** 10.1099/mgen.0.001248

**Published:** 2024-05-13

**Authors:** Nhat Ha Minh Truong, Quynh Nguyen, Phat Vinh Voong, Vinh Chau, Nhi Huynh Thanh Nguyen, Tuan Hoa Minh Nguyen, Phuong Hong Vo, Luan Thanh Nguyen, Trinh Thi Phuong Ha, Lan Phu Huong Nguyen, Phuoc Hong Le, Duy Pham Thanh, Hoang Duc Nguyen

**Affiliations:** 1 Center for Bioscience and Biotechnology, VNUHCM-University of Science, Ho Chi Minh City, Vietnam; 2 Vietnam National University, Ho Chi Minh City, Vietnam; 3 Oxford University Clinical Research Unit, Ho Chi Minh City, Vietnam; 4 Research Institute for Aquaculture No.2, Ho Chi Minh City, Vietnam; 5 Gene Solutions Company, Ho Chi Minh City, Vietnam

**Keywords:** *Aeromonas dhakensis*, *Aeromonas hydrophila*, antibiotic resistance, bloodstream infections, Motile *Aeromonas septicemia*, striped catfish, virulence

## Abstract

*Aeromonas* spp. are commonly found in the aquatic environment and have been responsible for motile *Aeromonas* septicemia (MAS) in striped catfish, resulting in significant economic loss. These organisms also cause a range of opportunistic infections in humans with compromised immune systems. Here, we conducted a genomic investigation of 87 *Aeromonas* isolates derived from diseased catfish, healthy catfish and environmental water in catfish farms affected by MAS outbreaks in eight provinces in Mekong Delta (years: 2012–2022), together with 25 isolates from humans with bloodstream infections (years: 2010–2020). Genomics-based typing method precisely delineated *Aeromonas* species while traditional methods such as *aerA* PCR and MALDI-TOF were unable identify *A. dhakensis. A. dhakensis* was found to be more prevalent than * A. hydrophila* in both diseased catfish and human infections. *A. dhakensis* sequence type (ST) 656 followed by *A. hydrophila* ST251 were the predominant virulent species-lineages in diseased catfish (43.7 and 20.7 %, respectively), while diverse STs were found in humans with bloodstream infections. There was evidence of widespread transmission of ST656 and ST251 on striped catfish in the Mekong Delta region. ST656 and ST251 isolates carried a significantly higher number of acquired antimicrobial resistance (AMR) genes and virulence factors in comparison to other STs. They, however, exhibited several distinctions in key virulence factors (i.e. lack of type IV pili and enterotoxin *ast* in *A. dhakensis*), AMR genes (i.e. presence of *imiH* carbapenemase in *A. dhakensis*), and accessory gene content. To uncover potential conserved proteins of *Aeromonas* spp. for vaccine development, pangenome analysis has unveiled 2202 core genes between ST656 and ST251, of which 78 proteins were in either outer membrane or extracellular proteins. Our study represents one of the first genomic investigations of the species distribution, genetic landscape, and epidemiology of *Aeromonas* in diseased catfish and human infections in Vietnam. The emergence of antimicrobial resistant and virulent *A. dhakensis* strains underscores the needs of enhanced genomic surveillance and strengthening vaccine research and development in preventing *Aeromonas* diseases in catfish and humans, and the search for potential vaccine candidates could focus on *Aeromonas* core genes encoded for membrane and secreted proteins.

**Impact Statement**

*Aeromonas* spp. are opportunistic pathogens in the aquatic environment and have been responsible for motile *Aeromonas* septicemia (MAS) in striped catfish farms along Vietnam’s Mekong Delta region. There are two prevalent MAS-causing *Aeromonas* species, namely *A. hydrophila* and *A. dhakensis*, which have been characterized by the presence of diverse virulence and antibiotic resistance traits. Even though epidemiological investigations of *Aeromonas* infections are urgently needed; such efforts were hindered by the lack of reliable species identification methods. Here, by using whole genome sequencing data we uncovered the predominance of *A. dhakensis* ST656 and *A. hydrophila* ST251 in diseased catfish and their geographical spread in the Mekong Delta region. These two species-lineages were undetected in human-derived isolates; instead, human bloodstream infections were caused by a diverse array of sequence types. Compared to *A. hydrophila*, the gene clusters encoding type IV pili and a heat-stable enterotoxin were missing from most of the *A. dhakensis* isolates. Generally, *A. hydrophila* and *A. dhakensis* isolates from diseased catfish contained more antimicrobial resistance genes and virulence factors compared to human-derived isolates. Our study provides new insights into the species distribution, highlighting the emergence of virulent and antimicrobial resistance lineage ST656 of *A. dhakensis* in Vietnam. We also revealed the distinctions in genotype distribution, virulence factors and AMR gene content between *A. dhakensis* ST656 and *A. hydrophila* ST251, as well as between aquaculture-derived and human-derived isolates. Our research will pave the way for further research in molecular surveillance and vaccine development to understand the transmission of *Aeromonas* in a broader context and prevent the MAS in catfish.

## Data Summary

Metadata associated with *Aeromonas* isolates from our study are included in Table S1 (available in the online version of this article). Accession numbers for *Aeromonas* sequencing are listed in Table S2.

## Introduction

Vietnam has been a significant producer of aquaculture products, notably striped catfish (*Pangasianodon hypophthalmus*), which is exported worldwide under the name pangasius [1]. The production of striped catfish is mainly concentrated in the Mekong Delta region and has a pivotal role in the regional economy with exports worth more than USD 1.7 billion [2]. However, outbreak of bacterial diseases, such as motile *Aeromonas* septicemia (MAS) caused by *Aeromonas hydrophila* or bacillary necrosis of pangasius (BNP) caused by *Edwardsiella ictaluri* can result in high mortality rates in striped catfish and have a detrimental impact on fish production [3].

Antibiotics are commonly used to treat diseased catfish during outbreak and decontaminate the water in the fish ponds [4]. However, antibiotic use can promote the development of antimicrobial resistance (AMR) in both environmental and the infecting organisms, primarily through the horizontal transfer of AMR plasmids [5,6]. The circulation of multidrug-resistant (MDR) *A. hydrophila* and *A. dhakensis* strains has been reported in fish farms in Northern and Southern Vietnam. These strains carried multiple AMR genes conferring resistance against various antibiotics, including oxacillin, amoxicillin, vancomycin, erythromycin, oxytetracycline, florfenicol, and sulfamethoxazole [7]. Consequently, there is a crucial need for developing inexpensive vaccines that are straightforward to deliver for preventing the diseases caused by *Aeromonas* in catfish and limiting the use of antibiotics.

The importance of vaccine development is further emphasized in the context of the emergence of hypervirulent *Aeromonas* (vAh) strains. Recent studies have shown the appearance of a vAH, namely sequence type (ST) 251*,* posing a serious concern in aquaculture globally. These strains have been responsible for several MAS outbreaks in farmed fish in the USA and China [8,9]. Moreover, isolates within this lineage have recently been detected in provinces farming striped catfish in Vietnam [10,11]. The vAh lineage possesses a diverse array of virulent factors and multiple AMR genes. Furthermore, *A. dhakensis* strains that display virulence in both catfish and humans have also emerged recently. *A. dhakensis* share a high nucleotide identity to *A. hydrophila* and thus was misclassified as *Aeromonas hydrophila* subsp*. dhakensis* [12]. Since 2013, *A. dhakensis* was formally acknowledged as a distinct *Aeromonas* species [13], encompassing what were previously classified as *Aeromonas hydrophila* subsp*. dhakensis* and *A. aquariorum* [14,15]. In the Mekong Delta region of Vietnam, the first report of diseased striped catfish caused by *A. dhakensis* was linked to the genotype ST656 [16].

*Aeromonas* spp. can cause life-threatening invasive infections in humans, particularly immunocompromised individuals. There has been very few research on human *Aeromonas* infections in Vietnam and the extent of genetic and species overlap to those present in non-human sources. Furthermore, traditional method such as biochemical testing, protein-based MALDI-TOF or gene-based 16S rRNA typing often fail to distinguish closely related *Aeromonas* species, hindering a comprehensive understanding of the population structure and transmission patterns of these organisms [17]. In this investigation, we utilized whole genome sequencing approach to precisely determine *Aeromonas* species*,* characterize the gene content and depict the genomic epidemiology of *Aeromonas* isolates derived from diseased striped catfish and human infections in Southern Vietnam.

## Methods

### Sampling sites and primary isolation of *Aeromonas* spp. isolates

Eighty-seven *Aeromonas* spp. strains were collected in different fish farms in the Mekong Delta region between 2012 and 2022, encompassing several provinces: An Giang, Vinh Long, Dong Thap, Tien Giang, Can Tho, Ben Tre. The sources of bacterial isolation included the kidneys or livers of diseased catfish, encompassing juvenile, fingerling, and grown-up specimens (*n*=63), as well as healthy catfish (*n*=12) from the same farms. Additionally, 12 water samples were also collected from the farms housing the diseased catfish. Samples were grown on Trypticase soy broth (TSB) or Brain Heart Infusion (BHI) broth overnight and suspected colonies were identified by API 20E biochemical testing, and the presence of MAS-causing *Aeromonas* was confirmed by PCR assay targeting the 16S rRNA and a 209 bp region of the aerolysin-encoding *aerA* gene [18]. Among 87 isolates derived from catfish and water samples, 31 isolates were further classified as either *A. hydrophila* or *A. dhakensis* by *rpoD* sequencing method [19]. Additionally, 25 *A*. *hydrophila* isolates (classified by MALDI-TOF) identified from patients with bloodstream infections at Hospital for Tropical Diseases, Ho Chi Minh City, Vietnam were included, giving rise to a total of 112 *Aeromonas* isolates.

### DNA extraction and whole genome sequencing

DNA was extracted from the overnight culture of 112 *Aeromonas* isolates using the Wizard Genomic DNA Extraction Kit (Promega, USA) following the manufacturer’s recommendations. DNA library preparation was done using a Nextera XT kit, followed by genome sequencing on an Illumina Miseq or NextSeq platform (Illumina, USA) to generate either 150 bp (for human-related isolates) or 75 bp paired-end reads (for catfish- and water-related isolates). Illumina reads quality assessment were performed by FASTQC [20] and *de novo* assembled using Unicycler v0.4.8 with default parameters [21]. The raw Illumina sequencing reads of each isolate have been deposited in ENA with the project accession PRJEB65955 (Table S2).

### Genomics-based species identification

**Fig. 1.**
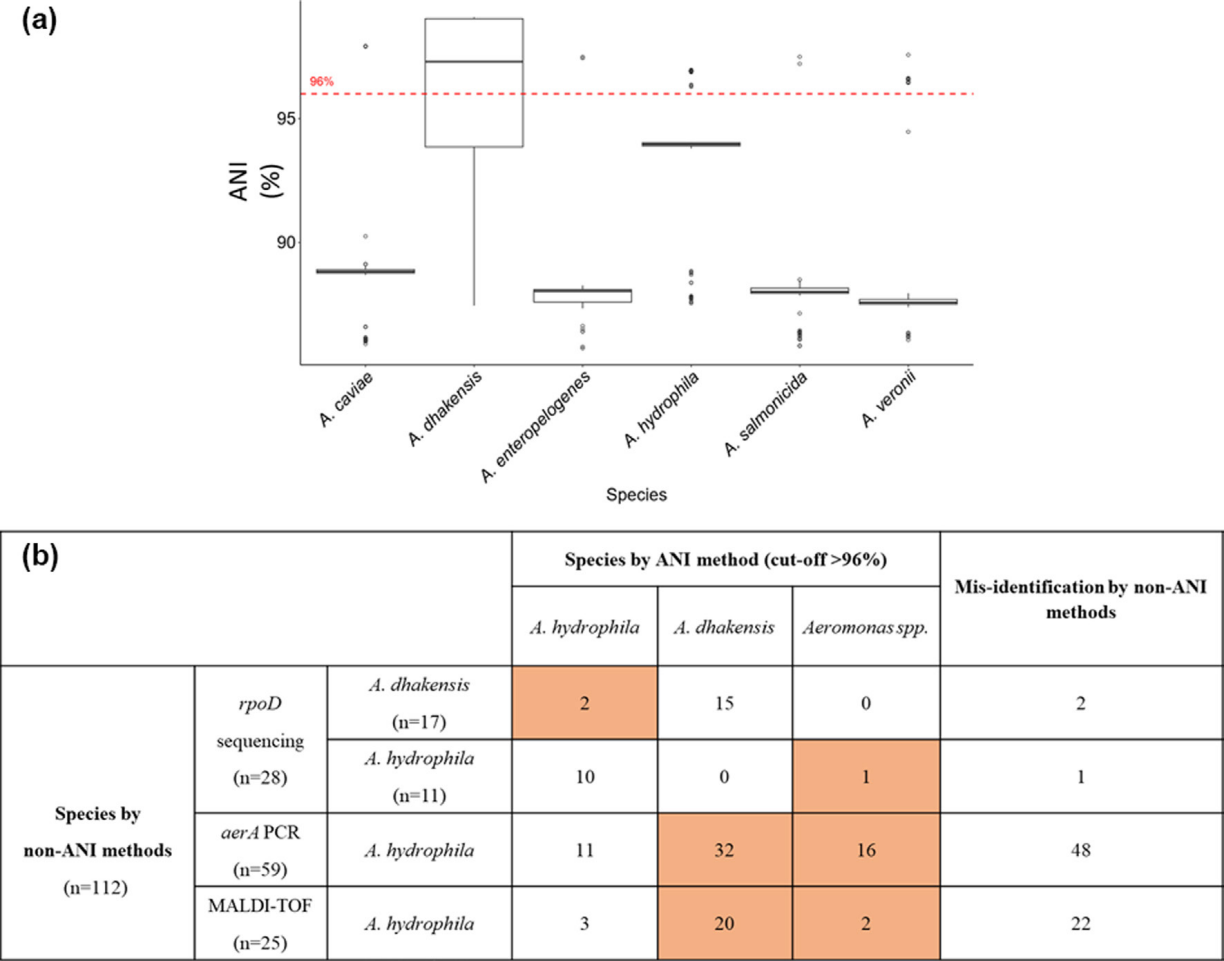
Comparison of different methods for species identification of *Aeromonas*. (a) The distribution of ANI values (represented in boxplot) between assembled genomes and each of the reference genomes. The dash line shows the ANI cut-off value of 96% for species identification. An ANI value above 96% indicates that the isolate belongs to the same species as the reference genome. (b) Statistics of agreement between *rpoD* sequencing, *aerA* PCR and MALDI-TOF in species identification. Colored cells indicate disagreements between non-ANI methods and ANI method.

A comparison of different methods for species identification of *Aeromonas* genus was conducted between PCR-based, gene-based sequencing and genomics-based methods. For the genomics-based approach, FastANI v1.32 [22] was employed to compute the Average Nucleotide Identity (ANI) value of each assembled genome in comparison to each of the reference genomes (Fig. 1a). The reference genomes were the complete genome of each *Aeromonas* species with the following accessions: GCF_016026875.1 (*A. hydrophila*), GCF_014169735.1 (*A. caviae*), GCF_020423125.1 (*A. media*), GCF_012931585.1 (*A. salmonicida*), GCF_020341435.1 (*A. enteropelogenes*), GCF_900637545.1 (*A. encheleia*), GCF_016127195.1 (*A. jandaei*), GCF_016026615.1 (*A. allosaccharophila*), GCF_008693705.1 (*A. veronii*), GCF_020149575.1 (*A. rivuli*), GCF_014892695.1 (*A. simiae*), GCF_020405345.1 (*A. dhakensis*), GCF_010974825.1 (*A. rivipollensis*). ANI is calculated as the average nucleotide identity of orthologous genes shared between two genomes, which has been widely accepted as a reliable tool for microbial taxonomy with high resolution and accuracy [12]. Based on the previous publication, an ANI value of 96 % was chosen as a threshold for species delineation of *Aeromonas* [11].

### Gene content analysis

SRST2 v0.2.0 [23] was used to determine the multi-locus sequence types (MLST), and the presence of virulence genes, AMR genes and plasmid replicons, using the respective databases: PubMLST (http://pubmlst.org), VFDB (http://www.mgc.ac.cn/VFs/), ARG-ANNOT v3 [24] and PlasmidFinder database [25]. Assembled genomes were annotated by Bakta v1.8.1 [26] and the pan-genome was reconstructed using ROARY v3.13 [27]. Protein’s cellular localization and topology were predicted by CELLO2GO v.2.5 [28] and DeepTMHMM v1.0.24 [29], both was accessed on 30 September 2023. Cogclassifier v1.0.5 [30] was used for functional classification of the pan-genome.

### Phylogenetic tree and data visualization

Based on the concatenated sequences of the six housekeeping genes from the MLST scheme (*gyrB*, *groL*, *gltA*, *metG*, *ppsA*, and *recA*), single nucleotide polymorphisms (SNP) were extracted using SNP-sites v2.5.1 [31], yielding a total of 203 SNPs (for *A. hydrophila*) and 306 SNPs (for *A. dhakensis*). Phylogenetic tree was reconstructed based on the resulting SNP alignment of each species, using maximum likelihood (ML) method and best-fit substitution model implemented in IQ-TREE v2.2.2.4 [32]. The ML tree was visualized and annotated using iTOL [33].

### Statistical analysis

T-test was used to analyse the differences in the number of virulence factors between *A. hydrophila* and *A. dhakensis*. *P* < 0.05 was considered to be statistically significant. The analysis was performed using the built-in ttest_ind function from the Scipy library written in Python programming language.

## Results

### Comparison of different methods for species identification of *Aeromonas*

Based on the ANI method, 110 out of 112 *Aeromonas* isolates were classified into the pre-defined species, with the ANI values ranging from 96.3–99.1 % (Fig. 1a). The majority of isolates (59.8 %, 67/112) were *A. dhakensis*, followed by *A. hydrophila* (24.1 %, 27/112), *A. veronii* (8.9 %, 10/112)*, A. caviae* (2.7 %, 3/112), *A. salmonicida* (1.8 %, 2/112) and *A. enteropelogenes* (1.8 %, 2/112). Two isolates were unclassified (ANI values<96 %), exhibiting the highest ANI values when compared to * A. rivipollensis* (95.3 %) and *A. veronii* (94.5 %).

We compared the genomics-based ANI method with *rpoD* sequencing, *aerA* PCR and MALDI-TOF for the identification of *Aeromonas* species, using the available data. Compared to the ANI method, MALDI-TOF and *aerA* PCR had the lowest agreement rates of 12 % (3/25) and 18.6 % (11/59), respectively; moreover, they misclassified *A. dhakensis* and other *Aeromonas* spp. as *A. hydrophila* (Fig. 1b). The *rpoD* sequencing method exhibited a high agreement rate with the ANI method (89.7 %, 26/29). However, it misidentified two *A. hydrophila* isolates as *A. dhakensis* and one *Aeromonas veronii* isolate as *A. hydrophila*.

### Distribution of sequence types

We sought to characterize the distribution of STs among isolates originating from catfish and water samples (aquaculture, *n*=87) and humans with BSIs (*n*=25) (Table 1). We found a predominance of *A. dhakensis* ST656 (43.7 %, 38/87) and *A. hydrophila* ST251 (20.7 %, 18/87) among isolates collected from aquaculture samples. Furthermore, 33.3 % (29/87) of isolates were designated as STNF, representing a group of novel STs not present in the existing MLST scheme from the PubMLST database. Conversely, a high diversity of STs was found among the BSI isolates and none of them belonged to ST656 and ST251. Out of 25 isolates from BSIs, 20 (80 %) were identified as *A. dhakensis*, three isolates (12 %) were *A. hydrophila* (12 %) and two isolates (8 %) were * A. veronii*. Among these, *A. dhakensis* isolates exhibited diverse STs, including ST252, ST337, ST407, ST540, ST1628, ST2068 and ST1330, with each ST represented by one or two isolates. The three *A. hydrophila* isolate were classified as ST515 (1) and STNF (2). Notably, 64 % (16/25) of isolates from BSIs were grouped into STNF.

**Table 1.** The distribution of sequence type (STs) within *Aeromonas* isolates

	Aquaculture-related isolates (*n*=87)					Human BSIs (*n*=25)								
**Species**	**ST656**	**ST251**	**ST445**	**ST1719**	**STNF**	**ST2068**	**ST540**	**ST1330**	**ST252**	**ST407**	**ST1628**	**ST337**	**ST517**	**STNF**
*A. hydrophila*	–	18	–	–	5	–	–	–	–	–	–	–	1	2
*A. dhakensis*	38	–	–	–	9	1	1	2	1	1	1	1	–	12
*A. enteropelogenes*	–	–	1	–	1	–	–	–	–	–	–	–	–	–
*A. veronii*	–	–	1	–	8	–	–	–	–	–	–	–	–	2
*A. salmonicida*	–	–	–	–	2	–	–	–	–	–	–	–	–	–
*A. rivipollensis*	–	–	–	–	1	–	–	–	–	–	–	–	–	–
*A. caviae*	–	–	–	–	3	–	–	–	–	–	–	–	–	–

### Distribution of virulence factors in *A. dhakensis* and *A. hydrophila* isolates

Here, we aimed to characterise and compare the distribution of key virulence factors between *A. dhakensis* and *A. hydrophila* isolates (Table 2). Among aquaculture-related isolates, the Type IV *Flp, Msh*, and *Tap* pili, which play a role in adherence and biofilm formation, were either missing (*Flp* and *Msh*) or incomplete (*Tap* with *tapB* gene missing) in *A. dhakensis* isolates. They were however detected in 78–100 % of *A. hydrophila* isolates. All *A. dhakensis* and *A. hydrophila* isolates harboured lateral flagella. The *A. dhakensis* isolates did not contain the genes responsible for polar flagella, whereas the *A. hydrophila* isolates possessed the flagellin-encoding *flaAB* genes but lacked the accessory factor modification gene *maf-1*. Notably, the distribution of secretion systems, toxins, and enzymes in aquaculture-related *A. hydrophila* and *A. dhakensis* isolates exhibited high similarities. The cytotoxic enterotoxin-encoding *AerA* gene was identified in 87 % (61/70) of *Aeromonas* isolates, while the hemolysin-encoding genes *ahh1* and *hlyA* were detected in all isolates. Intriguingly, 91.3 % (21/23) of *A. hydrophila* isolates possessed the heat-stable cytotonic enterotoxin *ast,* while only 2.1 % (1/47) of *A. dhakensis* carried this virulent factor. Meanwhile, the key enzymes such as protease, elastase, lipase, and nuclease were present in nearly all isolates. Among BSI-related isolates, both *Aeromonas* showed an absence of factors contributing to adhesion, with the only exception belonged to Msh pili in 33.3 % of *A. hydrophila* (1/3) and Tap pili in 20 % of *A. dhakensis* (4/20). Virulence factors involved with polar flagellum were undetected in both *Aeromonas*, while only *A. hydrophila* shown the presence of lateral flagella. Intriguingly, both aerolysin (*aerA*) and *rtx* were absent from human-related *A. hydrophila* but were detected in 35 % (7/20) and 85 % (17/20) of *A. dhakensis*, respectively. It is also noteworthy that most degradative enzymes did not present in both BSI-related *Aeromonas*, with only Exu1 being detected in 33.3 % (1/3) of *A. hydrophila* and EprA1 in 5 % (1/20) of *A. dhakensis*. Both secretion systems (T2SS and T6SS) were also detected in both *Aeromonas* with different distributions. Overall, the *A. dhakensis* and *A. hydrophila* isolates derived from aquaculture samples carried a significantly higher number of virulence factors in comparison to that of human BSI isolates (131.0 vs. 94.0, t-test two-tails, *P*=1.7×10^−16^, Table S3).

**Table 2.** Distribution of key virulence factors in *A. hydrophila* and *A. dhakensis* isolates

	Aquaculture-related samples(*n*=87)		Humans with bloodstream infections(*n*=23)	
	*A. hydrophila*	*A. dhakensis*	*A. hydrophila*	*A. dhakensis*
	(*n*=23)	(*n*=47)	(*n*=3)	(*n*=20)
**Motility and adhesion**				
Polar flagellum	78.3*	0	0	0
Lateral flagella	100	100	100	5
*Flp* type IV pilus	91.3	0	0	0
*Msh* type IV pilus	100	0	33.3	0
*Tap* type IV pilus	78.3	83.0*	0	20*
**Secretion system**				
T2SS	100	100	66.7	30
T6SS	95.7	85.1	33.3	75
**Toxin**				
Cytotoxic enterotoxin, AerA	87.0	87.2	0	35
Heat-labile hemolysin, AHH1	100	100	33.3	95
α-Hemolysin, HlyA	100	100	100	100
Heat-stable cytotonic enterotoxin, Ast	91.3	2.1	100	100
RtxA-H	88.5	97.9	0	85
**Enzyme**				
DNase-nuclease, Exu1	100	100	33.3	0
Elastase, AhpB	95.7	100	0	0
Extracellular protease, EprA1	100	100	0	5
Thermostable extracellular lipase, lip	100	100	0	0

*iIndicated incomplete/missing gene(s) responsible for the function of the virulence factors.

### Phylogenetic structure of *A. hydrophila* isolates

**Fig. 2.**
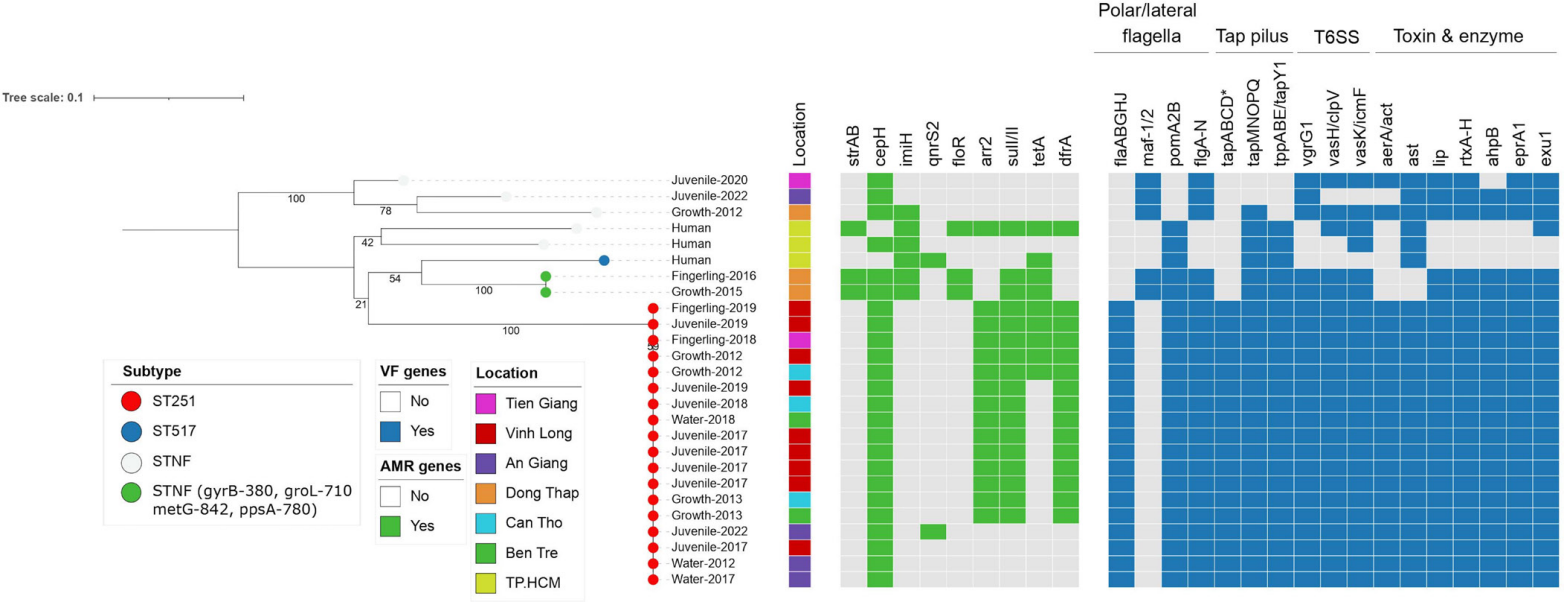
Maximum likelihood phylogenetic tree of *A. hydrophila* isolates from fish, water and bloodstream infections The node is coloured based on the sequence types (STs) of isolates. The first and second bar show the year and location of sample collection, respectively. The first heatmap illustrates the presence of acquired AMR genes. The second heatmap depicts the presence of key virulence factors. The tree scale represents the number of substitutions per site.

We sought to understand the genetic relatedness and transmission of *A. hydrophila* isolates as well as the distribution of AMR genes and virulence factors across different sequence types (STs). Therefore, a phylogenetic tree was reconstructed encompassing 23 isolates sourced from diseased catfish and water, and three isolates from human bloodstream infections (Table S1). All the ST251 isolates from infected fish and water samples formed a separate cluster on the phylogenetic tree, exhibiting limited genetic diversity (mean pairwise SNP distance of 37 SNPs), as shown in Fig. 2. These isolates were identified between 2012 and 2022 and were distributed across multiple provinces in the Mekong Delta region, indicating sustained and widespread transmission of this lineage. Overall, the non-ST251 isolates were distantly related to ST251 and displayed a higher degree of genetic diversity. ST251 isolates carried a higher abundance of virulence factors in comparison to other STs. More specifically, non-ST251 isolates were characterised by the absence of both *flaAB* and *tapA* genes, encoding for the polar flagella and the type IV *tap* pili, respectively (Fig. 2). Compared to the aquaculture-related isolates, isolates derived from human BSIs lacked a number of virulence factors, including the lateral flagella (*flgA-N*), T6SS (*vgrG*, *vasH*/*vasK*), protease (*eprA1*), elastase (*ahpB*), nuclease (*exu1*), lipase (*lip*) and repeat in toxin (RTX toxin). We observed that the aerolysin-encoding *aerA* gene was not found in seven out of eight non-ST251 isolates, providing further indication that aerolysin is not a suitable marker for the identification of *A. hydrophila*. Meanwhile, a novel ST (STNF: gyrB-380, groL-710, metG-842, ppsA-780) was detected in two isolates from diseased catfish between 2015 and 2016, characterised by possessing more virulence factors compared to other non-ST251. However, compared to ST251, they still lacked *flaAB, tapABCD, aerA,* and *lip* genes.

Regarding the presence of acquired AMR genes, most *A. hydrophila* isolates (92 %) carried the cephalosporin resistance gene *cepH*. Out of the 18 ST251 isolates, 14 isolates exhibited a similar AMR pattern of *dfrA-sulI/II-arr2-tetA* (+/−), predicted to confer resistance to rifampicin, cotrimoxazole, and/or tetracycline. Saliently, the carbapenem resistance gene *imiH* was only detected in non-ST251 isolates (6/8). With respect to non-ST251 isolates, an extensive array of AMR genes (*strAB-imiH-floR-sulI/II-tetA*) was identified in the two isolates belonging to the novel ST. The three isolates from BSIs showed three distinct AMR gene patterns of *cepH-imiH*, *imiH-qnrS2-tetA* and *strAB-imiH-floR-sulI/II-tetA-dfrA*.

### Phylogenetic structure of *A. dhakensis* isolates

**Fig. 3.**
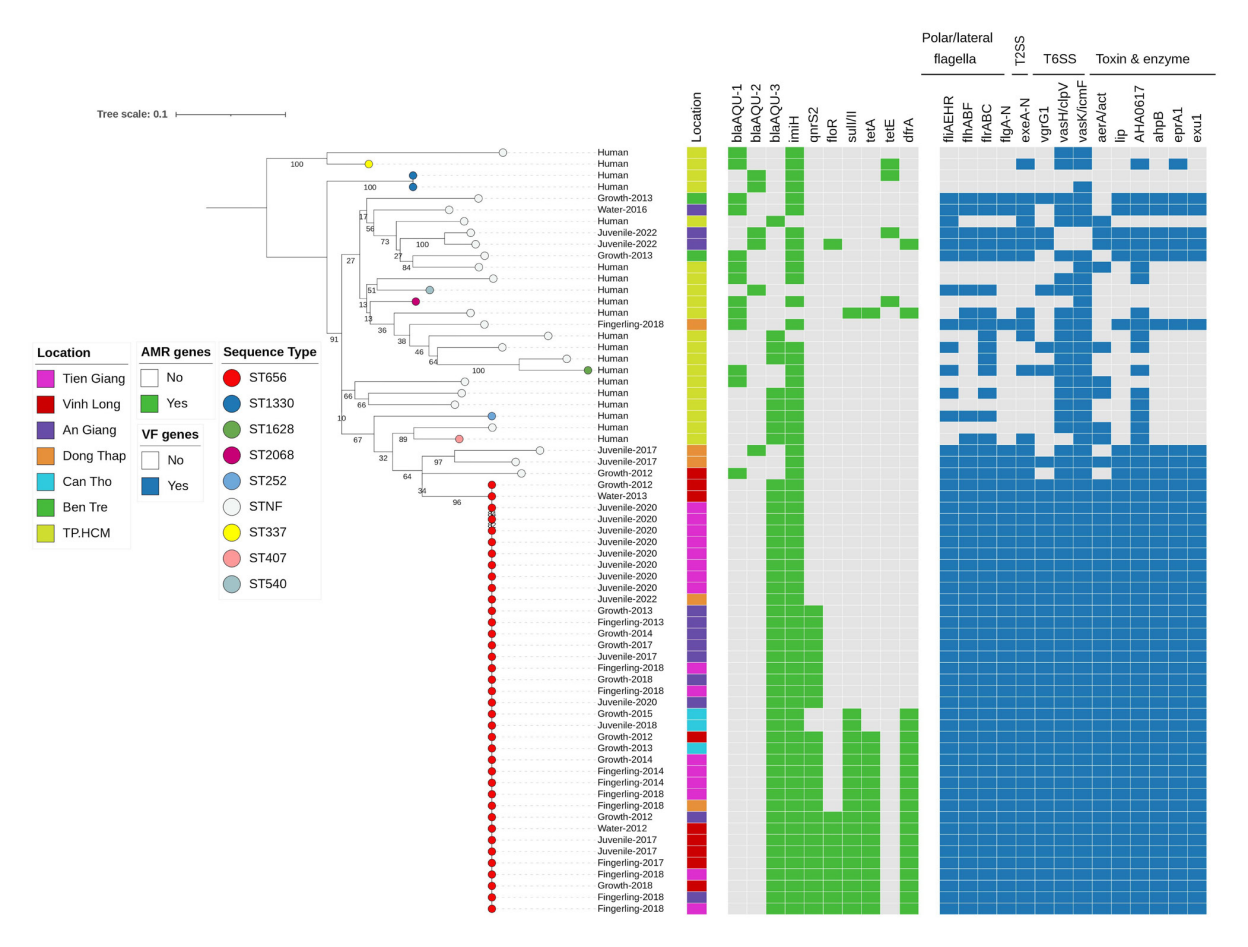
Maximum likelihood phylogenetic tree of *A. dhakensis* isolates from fish, water and bloodstream infections The node is coloured based on the sequence types (STs) of isolates. The first and second bar show the year and location of sample collection, respectively. The first heatmap illustrates the presence of acquired AMR genes. The second heatmap depicts the presence of key virulence factors. The tree scale represents the number of substitutions per site.

The phylogenetic tree of *A. dhakensis* isolates showed that the ST656 isolates from diseased catfish and water formed a separate lineage, with limited genetic variation (mean pairwise SNP distance of 71 SNPs) (Table S1, Fig. 3). Akin to *A. hydrophila* ST251, * A. dhakensis* ST656 appeared to spread across multiple provinces in the Mekong Delta. Conversely, the non-ST656 isolates exhibited a greater level of diversity, with 21/29 isolates belonging to novel STs (STNF), each exhibiting distinct allelic combinations. The isolates obtained from human BSIs were genetically distantly related. Again, we observed that the aquaculture-related isolates carried a significantly higher number of virulence factors compared to those derived from human BSIs (*P*<3.0×10^−9^). For example, the corresponding prevalence of intact flagella, T2SS, T6SS, toxins and enzymes in BSI-derived isolates were 0, 34.8, 8.7, and 62.4 %, compared to 62.9, 100, 88.6, and 93.4 % in aquaculture-related isolates.

Regarding the AMR gene profile, most *A. dhakensis* strains harboured a combination of the carbapenem resistance gene *imiH* (94.0 %) and a chromosomal class C β-lactamase *bla*_AQU_ gene (98.5 %) conferring resistance to cefotaxime. Compared to other STs, ST656 isolates possessed more AMR genes. Apart from the *imiH-bla*_AQU_ genes which were present in all ST656 isolates, they also carried different combinations with other AMR genes such as *qnrS2, floR, sul, tetA* and *dfrA* (Fig. 3).

### Pangenome analysis and localization prediction of core genes within *A. hydrophila* ST251 and *A. dhakensis* ST656

**Fig. 4.**
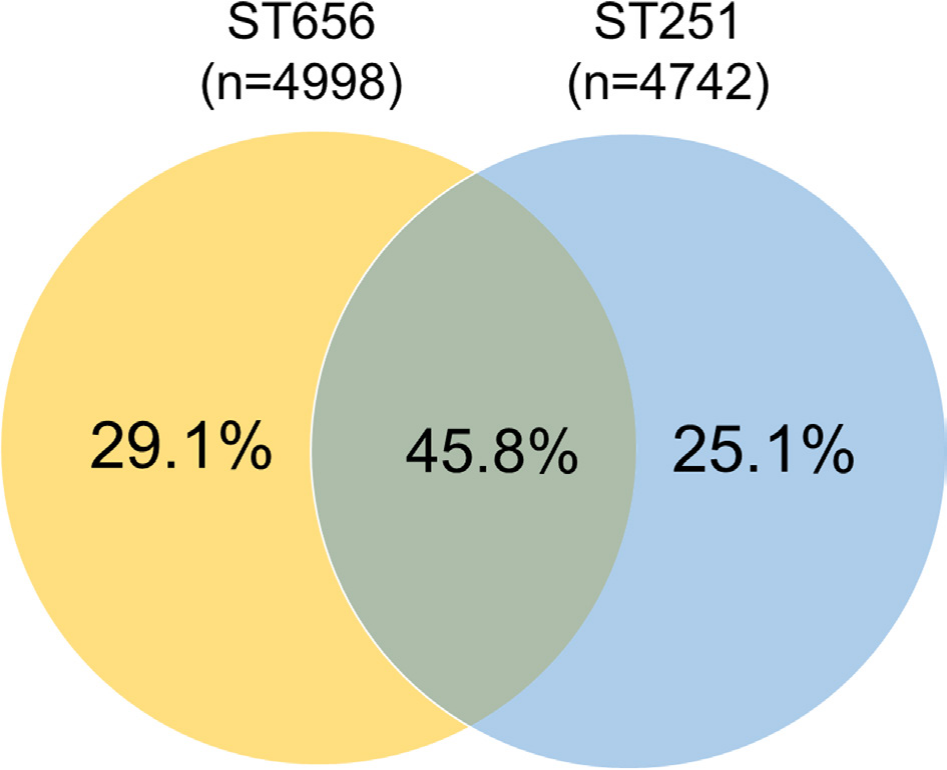
Pan-core genome content and functional analysis between *A. hydrophila* ST251 and *A. dhakensis* ST656.

A pangenome analysis was performed to investigate the genetic landscape and functional diversity between the two major species-lineages of *A. hydrophila*-ST251 and *A. dhakensis*-ST656 (Fig. 4). Out of 56 genomes of ST251 (*n*=18) and ST656 (*n*=38), the total number of genes found were 6782, of which 2202 genes were part of core genome (present in 99 % of the isolates). Of the 4580 accessory genes, 3306 were identified as shell genes (present in >15 % and <95 % of the isolates) and 874 were characterized as cloud genes (present in 15 % or less of the isolates).

To determine the cellular location of core genes, cellular localization prediction was performed by CELLO2GO on 2202 core genes, in which predicted outer membrane and extracellular proteins were further confirmed by DeepTMHMM. To this end, there were 18 proteins in the extracellular regions, 60 outer membrane proteins, 270 periplasmic proteins, 468 inner membrane proteins, and 1386 cytoplasmic proteins (Table S4). Further functional annotation of extracellular and outer membrane proteins by searching the Clusters of Orthologous Groups (COG) showed that 28.2 % (22/78) was contributing to cell wall/membrane/envelope biogenesis, 23.1 % (18/78) was related to transport pathways, 19.2 % (15/78) was related to cell motility, while proteins contributing to the post-translational modification or cell division only accounted for 6.4 % of the subset. Nevertheless, 21.8 % of the extracellular/outer membrane proteins were still of unknown function, which required more research to accurately annotate.

## Discussion

In this study, by comparing genomics-based ANI with other typing methods in the identification of *Aeromonas* species, we found the inaccuracy of gene-based typing (*aerA* PCR) or protein-based method (MALDI-TOF) in the detection of *A. dhakensis.* The lack of discriminatory power of these traditional methods might have resulted in a common perception that *A. hydrophila* is the primary causative agent of diseases in fish and humans [34]. In fact, our study findings revealed that *A. dhakensis* is a dominant cause of infections in both catfish (77 %) and bloodstream infections in humans (80 %). This result aligned with previous study showing the emergence of *A. dhakensis* in human infections [17,35] and in catfish from Vietnam [16,34]. However, our study also uncovered the presence of other *Aeromonas* species presented in the aquaculture-related samples: *A. veronii, A. caviae*, *A. salmonicida* and * A. enteropelogenes*. Among these, *A. veronii*, an emerging human enteric pathogen [36], was identified in both aquaculture and human BSI. Our research underscores the importance of developing novel markers, updating the existing MALDI-TOF database, or employing sequencing-based methods for a more comprehensive characterization of these species as well as their respective epidemiological and genetic profiles. Furthermore, research on vaccine and therapeutics may consider shifting their focus toward *A. dhakensis* given its significance and genomic dissimilarity from *A. hydrophila*.

*Aeromonas* are widely distributed in aquatic environment and can cause severe bacteremia in immunocompromised humans with a high mortality rate [37]. Our study represents the first genomic investigation of *Aeromonas* isolates causing bloodstream infections in humans in Vietnam. The source of *Aeromonas* bacteremia may involve ingestion of contaminated food or water followed by intestinal colonization and gut translocation in people with weakened immune system [38]. Here, human *Aeromonas* bacteremia is attributed to a wide range of sequence types (STs), in contrast to motile *Aeromonas* septicemia (MAS) in catfish, which is primarily caused by * A. hydrophila*-ST251 and *A. dhakensis*-ST656. While the presence of novel ST in human *Aeromonas* spp. was well within expectations [39], the presence of new STs in Vietnam aquaculture samples was unprecedented. *Aeromonas* isolates from humans with BSIs also exhibited distinct AMR and virulence gene profiles compared to the aquaculture-related isolates. These findings indicate that catfish exposure or catfish consumption is unlikely to be a source of human infections.

The *A. hydrophila* and *A. dhakensis* isolates derived from diseased catfish and water possessed a remarkably high number of virulence factors, encompassing motility, adhesion, secretion systems, various toxins and enzymes. Conversely, many virulence factors such as lateral flagella, T6SS, degradative enzymes (protease, elastase, nuclease, lipase) and toxin (RTX toxin) were either missing or present with significantly lower prevalence in human BSI-derived isolates. This finding implies that the virulence factors that are crucial for causing diseases in catfish may not necessarily have the same significance in human disease. Furthermore, there was an inherent difference between *A. hydrophila* and *A. dhakensis* regarding genes contributing to adhesions. Specifically, genes involving in the biogenesis and function of *msh, flp* and *tap* type IV pilus was absent in *A. dhakensis*, which further supports the notion that type IV pili might not be fundamental for the pathogenesis of *Aeromonas* [40]. Furthermore, the heat-stable cytotonic enterotoxin Ast, which was linked to clinical gastroenteritis [41] and increased fluid secretion in mouse model [42] was not detected in 98 % of * A. dhakensis*. Collectively, we reckon, the pathogenesis of *A. hydrophila* and *A. dhakensis* infections may have distinct features, warranting more in-depth investigations. We also observed that the two dominant-genotypes of *A. hydrophila*-ST251 and * A. dhakensis*-ST656 contained more virulence factors than other aquaculture-related isolates. For example, the mobility and adhesion factors were missing in non-ST251 *A. hydrophila*, and the *aerA* hemolysin was absent in non-ST251 *A. hydrophila* and non-ST656 * A. dhakensis* isolates. We speculate that the two prevalent genotypes of ST251 and ST656 are the main culprits for MAS in catfish, while other genotypes coexist in the same ecological niches.

Overall, *A. hydrophila* and *A. dhakensis* isolates carried the species-specific chromosomal AmpC β-lactamases of *bla_CepH_* and *bla_AQU,_* respectively*.* These enzymes can hydrolyse cephamycins and third generation cephalosporins, including clavulanic acid, tazobactam, and sulbactam [43]. Additionally, 23 % of *A. hydrophila* and 94 % of *A. dhakensis* isolates contained the class B MBL of *imiH* carbapenemase. *ImiH* has been sporadically reported in *A. hydrophila* [44] but is rarely documented in *A. dhakensis*. The genetic context surrounding the *imiH* gene is similar among the *imiH*-carrying *A. hydrophila* and *A. dhakensis* isolates, suggesting that the gene might has been horizontally transferred between the two species. In general, *A. hydrophila*-ST251 and * A. dhakensis*-ST656 isolates carried more AMR genes compared to non-ST251 and non-ST656 aquaculture-related isolates as well as human-derived isolates. The acquisition of multiple AMR genes may facilitate the survival and transmission of these virulent lineages in the aquatic environment.

With the relevance of multi-resistance motile *Aeromonas*, it is preferable to develop vaccines as a control measure against both * A. hydrophila* and *A. dhakensis.* To address this, a pangenome approach has been conducted to identify 2202 core genes shared by both ST251 *A. hydrophila* and ST656 *A. dhakensis*. As outer membrane/beta-barrel and extracellular/secreted proteins have been promising targets for vaccine development [45,46], a list of outer membrane and extracellular proteins were identified from the core gene subset. Further immunoinformatic analysis and *in vitro* validation would be necessary to determine a final vaccine against both *A. hydrophila* and *A. dhakensis* infections. Additionally, functional classification of these outer membrane/extracellular proteins was shown to be skewed toward cell wall/membrane/envelope biogenesis, transport pathways, and cell motility, implied that these pathways could be vital for *Aeromonas*’s pathogenesis.

In conclusion, our study represents one of the first genomic investigations of the species distribution, genetic landscape, and epidemiology of *Aeromonas* infections in catfish and humans. *A. dhakensis* has emerged as the primary cause of severe invasive diseases in both catfish and humans. We report the predominance of the two species-lineage of *A. dhakensis*-ST656 and *A. hydrophila*-ST251, responsible for motile *Aeromonas* septicemia in catfish. In contrast, human bloodstream infections are caused by a diverse array of sequence types. ST251 and ST656 isolates carried higher numbers of antimicrobial resistance and virulence genes compared to non-ST251 and non-ST656 isolates from aquaculture and human bloodstream infections. We also identify the presence of *imiH* carbapenemase in almost all *A. dhakensis* isolates, raising concern about difficult-to-treat infections caused by these organisms. We urge for more genomics-based research to better understand the emergence, reservoirs, transmission, and pathogenesis of different *Aeromonas* species-lineages. Future vaccine research and development should tailor to target both *A. dhakensis* ST656 and *A. hydrophila* ST251.

## supplementary material

10.1099/mgen.0.001248 Table S1.

## Abbreviations

AMR: antimicrobial resistance
ANI: average nucleotide identity
BSI: bloodstream infection
iToL: interactive Tree of Life
MALDI-TOF: Matrix-assisted laser desorption ionization /Time of Flight
MAS: motile Aeromonas septicemia
MDR: multi-drug resistance
ML: maximum-likelihood
MLST: multi-locus sequence typing
SNP: Single nucleotide polymorphism

## References

[1] Phuong NT, Oanh DTH, De Silva SS, FB FB (2010). Success Stories Asian Aquac.

[2] Hasan MR, Shipton TA (2021). Aquafeed value chain analysis of striped catfish in Vietnam. Aquaculture.

[3] Phu TM, Phuong NT, Dung TT, Hai DM, Son VN (2016). An evaluation of fish health-management practices and occupational health hazards associated with Pangasius catfish (*Pangasianodon hypophthalmus*) aquaculture in the Mekong Delta, Vietnam. Aquac Res.

[4] Phu TM, Phuong NT, Scippo M-L, Dalsgaard A (2015). Quality of antimicrobial products used in striped catfish (*Pangasianodon hypophthalmus*) aquaculture in Vietnam. PLoS One.

[5] Bello-López JM, Cabrero-Martínez OA, Ibáñez-Cervantes G, Hernández-Cortez C, Pelcastre-Rodríguez LI (2019). Horizontal gene transfer and its association with antibiotic resistance in the genus *Aeromonas* spp. Microorganisms.

[6] Ström GH, Björklund H, Barnes AC, Da CT, Nhi NHY (2019). Antibiotic use by small-scale farmers for freshwater aquaculture in the Upper Mekong Delta, Vietnam. J Aquat Anim Health.

[7] Nhinh DT, Le DV, Van KV, Huong Giang NT, Dang LT (2021). Prevalence, virulence gene distribution and alarming the multidrug resistance of *Aeromonas hydrophila* associated with disease outbreaks in freshwater aquaculture. Antibiotics.

[8] Griffin MJ, Goodwin AE, Merry GE, Liles MR, Williams MA (2013). Rapid quantitative detection of *Aeromonas hydrophila* strains associated with disease outbreaks in catfish aquaculture. J Vet Diagn Invest.

[9] Zhang X, Yang W, Wu H, Gong X, Li A (2014). Multilocus sequence typing revealed a clonal lineage of *Aeromonas hydrophila* caused motile *Aeromonas septicemia* outbreaks in pond-cultured cyprinid fish in an epidemic area in central China. Aquaculture.

[10] Ngo TPH, Vu HTT, Le TTT, Bui HCN, Liles MR (2022). Comparative genomic analysis of hypervirulent *Aeromonas hydrophila* strains from striped catfish (*Pangasianodon hypophthalmus*) in Vietnam. Aquaculture.

[11] Rasmussen-Ivey CR, Hossain MJ, Odom SE, Terhune JS, Hemstreet WG (2016). Classification of a hypervirulent *Aeromonas hydrophila* pathotype responsible for epidemic outbreaks in warm-water fishes. Front Microbiol.

[12] Rasmussen-Ivey CR, Figueras MJ, McGarey D, Liles MR (2016). Virulence factors of *Aeromonas hydrophila*: in the wake of reclassification. Front Microbiol.

[13] Beaz-Hidalgo R, Martínez-Murcia A, Figueras MJ (2013). Reclassification of *Aeromonas hydrophila* subsp. dhakensis Huys *et al*. 2002 and *Aeromonas aquariorum* Martínez-Murcia *et al*. 2008 as *Aeromonas dhakensis* sp. nov. comb nov. and emendation of the species *Aeromonas hydrophila*. Syst Appl Microbiol.

[14] Huys G, Kämpfer P, Albert MJ, Kühn I, Denys R (2002). *Aeromonas hydrophila* subsp. dhakensis subsp. nov., isolated from children with diarrhoea in Bangladesh, and extended description of Aeromonas hydrophila subsp. hydrophila (Chester 1901) Stanier 1943 (approved lists 1980). Int J Syst Evol Microbiol.

[15] Martínez-Murcia A, Monera A, Alperi A, Figueras M-J, Saavedra M-J (2009). Phylogenetic evidence suggests that strains of *Aeromonas hydrophila* subsp. dhakensis belong to the species *Aeromonas aquariorum* sp. nov. Curr Microbiol.

[16] Bartie KL, Ngô TPH, Bekaert M, Hoang Oanh DT, Hoare R (2022). *Aeromonas hydrophila* ST251 and *Aeromonas dhakensis* are major emerging pathogens of striped catfish in Vietnam. Front Microbiol.

[17] Fernández-Bravo A, Figueras MJ (2020). An update on the genus *Aeromonas*: taxonomy, epidemiology, and pathogenicity. Microorganisms.

[18] Pollard DR, Johnson WM, Lior H, Tyler SD, Rozee KR (1990). Detection of the aerolysin gene in *Aeromonas hydrophila* by the polymerase chain reaction. J Clin Microbiol.

[19] Pridgeon JW, Klesius PH (2011). Molecular identification and virulence of three *Aeromonas hydrophila* isolates cultured from infected channel catfish during a disease outbreak in west Alabama (USA) in 2009. Dis Aquat Organ.

[20] Andrews S (2010). Babraham bioinformatics - fastQC a quality control tool for high throughput sequence data. https://www.bioinformatics.babraham.ac.uk/projects/fastqc/.

[21] Wick RR, Judd LM, Gorrie CL, Holt KE (2017). Unicycler: resolving bacterial genome assemblies from short and long sequencing reads. PLoS Comput Biol.

[22] Jain C, Rodriguez-R LM, Phillippy AM, Konstantinidis KT, Aluru S (2018). High throughput ANI analysis of 90K prokaryotic genomes reveals clear species boundaries. Nat Commun.

[23] Inouye M, Dashnow H, Raven L-A, Schultz MB, Pope BJ (2014). SRST2: rapid genomic surveillance for public health and hospital microbiology labs. Genome Med.

[24] Gupta SK, Padmanabhan BR, Diene SM, Lopez-Rojas R, Kempf M (2014). ARG-ANNOT, a new bioinformatic tool to discover antibiotic resistance genes in bacterial genomes. Antimicrob Agents Chemother.

[25] Carattoli A, Zankari E, García-Fernández A, Voldby Larsen M, Lund O (2014). In silico detection and typing of plasmids using PlasmidFinder and plasmid multilocus sequence typing. Antimicrob Agents Chemother.

[26] Schwengers O, Jelonek L, Dieckmann MA, Beyvers S, Blom J (2021). Bakta: rapid and standardized annotation of bacterial genomes via alignment-free sequence identification. Microb Genom.

[27] Page AJ, Cummins CA, Hunt M, Wong VK, Reuter S (2015). Roary: rapid large-scale prokaryote pan genome analysis. Bioinformatics.

[28] Yu C-S, Lin C-J, Hwang J-K (2004). Predicting subcellular localization of proteins for Gram-negative bacteria by support vector machines based on n-peptide compositions. Protein Sci.

[29] Hallgren J, Tsirigos KD, Pedersen MD, Almagro Armenteros JJ, Marcatili P (2022). DeepTMHMM predicts alpha and beta transmembrane proteins using deep neural networks. Bioinformatics.

[30] Shimoyama Y (2022). Cogclassifier: a tool for classifying Prokaryote protein sequences into COG functional category.

[31] Page AJ, Taylor B, Delaney AJ, Soares J, Seemann T (2016). *SNP-sites*: rapid efficient extraction of SNPs from multi-FASTA alignments. Microb Genom.

[32] Minh BQ, Schmidt HA, Chernomor O, Schrempf D, Woodhams MD (2020). IQ-TREE 2: new models and efficient methods for phylogenetic inference in the genomic era. Mol Biol Evol.

[33] Letunic I, Bork P (2021). Interactive tree of life (iTOL) v5: an online tool for phylogenetic tree display and annotation. Nucleic Acids Res.

[34] Erickson VI, Khoi LM, Hounmanou YMG, Dung TT, Phu TM (2023). Comparative genomic analysis of *Aeromonas dhakensis* and *Aeromonas hydrophila* from diseased striped catfish fingerlings cultured in Vietnam. Front Microbiol.

[35] Chen P-L, Lamy B, Ko W-C (2016). Aeromonas dhakensis, an increasingly recognized human pathogen. Front Microbiol.

[36] Liu F, Yuwono C, Tay ACY, Wehrhahn MC, Riordan SM (2022). Analysis of global *Aeromonas veronii* genomes provides novel information on source of infection and virulence in human gastrointestinal diseases. BMC Genom.

[37] Zhao Y, Alexander J (2021). *Aeromonas hydrophilia* infection in an immunocompromised host. Cureus.

[38] Bhowmick UD, Bhattacharjee S (2018). Bacteriological, clinical and virulence aspects of *Aeromonas*-associated diseases in humans. Pol J Microbiol.

[39] Lau TTV, Tan J-AMA, Puthucheary SD, Puah S-M, Chua K-H (2020). Genetic relatedness and novel sequence types of clinical *Aeromonas dhakensis* from Malaysia. Braz J Microbiol.

[40] Boyd JM, Dacanay A, Knickle LC, Touhami A, Brown LL (2008). Contribution of type IV pili to the virulence of *Aeromonas salmonicida* subsp. salmonicida in Atlantic salmon (*Salmo salar* L.). Infect Immun.

[41] Albert MJ, Ansaruzzaman M, Talukder KA, Chopra AK, Kuhn I (2000). Prevalence of enterotoxin genes in *Aeromonas* spp. isolated from children with diarrhea, healthy controls, and the environment. J Clin Microbiol.

[42] Li J, Ni XD, Liu YJ, Lu CP (2011). Detection of three virulence genes alt, ahp and aerA in *Aeromonas hydrophila* and their relationship with actual virulence to zebrafish. J Appl Microbiol.

[43] Wu C-J, Wang H-C, Chen P-L, Chang M-C, Sunny Sun H (2013). AQU-1, a chromosomal class C β-lactamase, among clinical *Aeromonas dhakensis* isolates: distribution and clinical significance. Int J Antimicrob Agents.

[44] Niumsup P, Simm AM, Nurmahomed K, Walsh TR, Bennett PM (2003). Genetic linkage of the penicillinase gene, amp, and blrAB, encoding the regulator of beta-lactamase expression in *Aeromonas* spp. J Antimicrob Chemother.

[45] Guo Z, Lin Y, Wang X, Fu Y, Lin W (2018). The protective efficacy of four iron-related recombinant proteins and their single-walled carbon nanotube encapsulated counterparts against *Aeromonas hydrophila* infection in zebrafish. Fish Shellfish Immunol.

[46] Awan F, Ali MM, Dong Y, Yu Y, Zeng Z (2021). In silico analysis of potential outer membrane beta-barrel proteins in *Aeromonas hydrophila* pangenome. Int J Pept Res Ther.

