# Supplementary material for: Genomic characterization of Aeromonas spp. isolates from striped catfish with motile Aeromonas septicemia and human bloodstream infections in Vietnam
Source: Microb Genom. 2024 May 13;10(5):001248. doi: 10.1099/mgen.0.001248 (PMC11165649; doi:10.1099/mgen.0.001248)
Supplement: Table S1. [file mgen-10-01248-s001.pdf]

Table S1. Isolate details including collection date and geographic origin, host species and statuses, species identification methods, ANI values, and sequence typing results.

Isolate	Year	Farm location	Diseased/healthy fish	Source	Identify by *aerA* PCR method	Identify by rpoD sequencing method	Identify by MALDI-TOF method	Identify by ANI method	ANI (%)	ST	gyrB	groL	gltA	metG	ppsA	recA
1-AD01	2017	An Giang	Diseased	Juvenile	*A. hydrophila*	*A. dhakensis*		*A. dhakensis*	99.0317	656	415	164	160	267	457	160
AD02	2017	Vinh Long	Diseased	fingerling	*A. hydrophila*	*A. dhakensis*		*A. dhakensis*	99.0459	656	415	164	160	267	457	160
3-AH03	2017	Vinh Long	Diseased	Juvenile	*A. hydrophila*	*A. hydrophila*		*A. hydrophila*	96.9577	251	210	214	122	211	221	217
4-AH04	2017	Vinh Long	Diseased	Juvenile	*A. hydrophila*	*A. hydrophila*		*A. hydrophila*	96.9072	251	210	214	122	211	221	217
5-AH05	2017	Vinh Long	Diseased	Juvenile	*A. hydrophila*	*A. hydrophila*		*A. hydrophila*	96.9292	251	210	214	122	211	221	217
24-AH06	2017	Vinh Long	Diseased	Juvenile	*A. hydrophila*	*A. hydrophila*		*A. hydrophila*	96.909	251	210	214	122	211	221	217
AH07	2017	Vinh Long	Diseased	Juvenile	*A. hydrophila*	*A. hydrophila*		*A. hydrophila*	96.9432	251	210	214	122	211	221	217
26-AD08	2017	Vinh Long	Diseased	Juvenile	*A. hydrophila*	*A. dhakensis*		*A. dhakensis*	99.044	656	415	164	160	267	457	160
6-AD9	2017	Vinh Long	Diseased	Juvenile	*A. hydrophila*	*A. dhakensis*		*A. dhakensis*	99.0391	656	415	164	160	267	457	160
8-AD10	2017	Dong Thap	Diseased	Juvenile	*A. hydrophila*	*A. dhakensis*		*A. dhakensis*	97.3982	NF*	444	379	644*	388	288	423
9-AD11	2017	Dong Thap	Diseased	Juvenile	*A. hydrophila*	*A. dhakensis*		*A. dhakensis*	97.2989	NF*	279	387*	812	913	421*	430
AH13	2017	An Giang	Diseased	fingerling	*A. hydrophila*	*A. hydrophila*		*A. hydrophila*	96.9394	251	210	214	122	211	221	217
7-AD16	2018	An Giang	Diseased	fingerling	*A. hydrophila*	*A. dhakensis*		*A. dhakensis*	99.0436	656	415	164	160	267	457	160
3-AH17	2018	Can Tho	Diseased	juvenile	*A. hydrophila*	*A. hydrophila*		*A. hydrophila*	96.9412	251	210	214	122	211	221	217
18-AD18	2018	Can Tho	Diseased	juvenile	*A. hydrophila*	*A. dhakensis*		*A. dhakensis*	99.0562	656	415	164	160	267	457	160
19-AD19	2018	Dong Thap	Healthy	fingerling	*A. hydrophila*	*A. dhakensis*		*A. dhakensis*	97.1887	NF*	496*	379*	391	383	1115*	162
AD21	2018	Dong Thap	Healthy	fingerling	*A. hydrophila*	*A. dhakensis*		*A. dhakensis*	99.0894	656	415	164	160	267	457	160
8-AH22	2018	Ben Tre	Diseased	juvenile	*A. hydrophila*	*A. hydrophila*		*A. hydrophila*	96.9249	251	210	214	122	211	221	217
8-AD23	2018	Tien Giang	Diseased	fingerling	*A. hydrophila*	*A. dhakensis*		*A. dhakensis*	99.0534	656	415	164	160	267	457	160
32-AD24	2018	Tien Giang	Diseased	fingerling	*A. hydrophila*	*A. dhakensis*		*A. dhakensis*	99.0249	656	415	164	160	267	457	160
4-AD25	2018	Tien Giang	Diseased	fingerling	*A. hydrophila*	*A. dhakensis*		*A. dhakensis*	99.0529	656	415	164	160	267	457	160
10-AD26	2018	Tien Giang	Diseased	Fingerling	*A. hydrophila*	*A. dhakensis*		*A. hydrophila*	96.8964	251	210	214	122	211	221	217
11-AD27	2018	Tien Giang	Diseased	fingerling	*A. hydrophila*	*A. dhakensis*		*A. dhakensis*	99.0683	656	415	164	160	267	457	160
9-AD29	2018	Tien Giang	Diseased	fingerling	*A. hydrophila*	*A. dhakensis*		*A. dhakensis*	99.0366	656	415	164	160	267	457	160
5-AD32	2019	Vinh Long	Diseased	fingerling	*A. hydrophila*	*A. dhakensis*		*A. hydrophila*	96.9087	251	210	214	122	211	221	217
37-AH33	2019	Vinh Long	Diseased	juvenile	*A. hydrophila*	*A. hydrophila*		*A. hydrophila*	96.9102	251	210	214	122	211	221	217
10-AH34	2019	Vinh Long	Diseased	Juvenile	*A. hydrophila*	*A. hydrophila*		*A. hydrophila*	96.8799	251	210	214	122	211	221	217
6-AH35	2019	Vinh Long	Diseased	juvenile	*A. hydrophila*	*A. hydrophila*		*A. veronii*	97.5708	1719	66	71	110	411	224	223
9-AH36	2020	Tien Giang	Diseased	juvenile	*A. hydrophila*			*A. dhakensis*	99.0436	656	415	164	160	267	457	160
10-AH37	2020	Tien Giang	Diseased	juvenile	*A. hydrophila*			*A. dhakensis*	99.0005	656	415	164	160	267	457	160
20-AH38	2020	Tien Giang	Diseased	juvenile	*A. hydrophila*			*A. dhakensis*	99.0565	656	415	164	160	267	457	160
11-AH39	2020	Tien Giang	Diseased	juvenile	*A. hydrophila*			*A. dhakensis*	99.0904	656	415	164	160	267	457	160
31-AH40	2020	Tien Giang	Diseased	juvenile	*A. hydrophila*			*A. hydrophila*	96.3295	NF*	251	260	272	264*	957*	1069
12-AH41	2020	Tien Giang	Diseased	juvenile	*A. hydrophila*			*A. dhakensis*	99.0244	656	415	164	160	267	457	160
21-AH42	2020	Tien Giang	Diseased	juvenile	*A. hydrophila*			*A. dhakensis*	99.0178	656	415	164	160	267	457	160
22-AH43	2020	Tien Giang	Diseased	juvenile	*A. hydrophila*			*A. dhakensis*	99.0012	656	415	164	160	267	457	160
23-AH44	2020	Tien Giang	Diseased	juvenile	*A. hydrophila*			*A. dhakensis*	99.0541	656	415	164	160	267	457	160
24-AH100	2020	An Giang	Diseased	Juvenile	*A. hydrophila*			*A. dhakensis*	99.0609	656	415	164	160	267	457	160
25-AH121	2022	An Giang	Diseased	Juvenile	*A. hydrophila*			*A. salmonicida*	97.2113	NF*	2	2*	284	22*	198*	184
26-AH122	2022	An Giang	Diseased	Juvenile	*A. hydrophila*			*A. veronii*	96.5802	NF	214	226	302	216	124	233
27-AH123	2022	An Giang	Diseased	Juvenile	*A. hydrophila*			*A. veronii*	96.5847	NF	214	226	302	216	124	233

28-AH124	2022	An Giang	Diseased	Juvenile	*A. hydrophila*			*A. veronii*	96.4429	NF	813	154	311	148	910	330
29-AH125	2022	An Giang	Diseased	Juvenile	*A. hydrophila*			*A. rivipollensis*	95.2553	NF*	790*	201*	831*	849	194*	187*
27-AH126	2022	An Giang	Diseased	Juvenile	*A. hydrophila*			*A. veronii*	96.5665	NF*	655*	456*	335	756	454*	87*
28-AH127	2022	An Giang	Diseased	Juvenile	*A. hydrophila*			*A. enteropelogenes*	97.4905	NF*	574	743	856	576	764*	761*
30-AH129	2022	An Giang	Diseased	Juvenile	*A. hydrophila*			*A. enteropelogenes*	97.4403	445	95	104	328	332	356	100
30-AH130	2022	An Giang	Diseased	Juvenile	*A. hydrophila*			*A. veronii*	96.4558	NF*	223*	392	617*	141*	343*	464*
31-AH132	2022	An Giang	Diseased	Juvenile	*A. hydrophila*			*A. hydrophila*	96.9362	251	210	214	122	211	221	217
33-AH133	2022	An Giang	Diseased	Juvenile	*A. hydrophila*			*A. dhakensis*	97.3507	NF*	386*	469*	317*	496	438	637
34-AH134	2022	An Giang	Diseased	Juvenile	*A. hydrophila*			*A. dhakensis*	97.3276	NF*	510*	262	317*	496	438	637
35-AH135	2022	An Giang	Diseased	Juvenile	*A. hydrophila*			*A. hydrophila*	96.367	NF*	251*	246	383*	264	283*	365
36-AH137	2022	Dong Thap	Diseased	Juvenile	*A. hydrophila*			*A. dhakensis*	99.03	656	415	164	160	267	457	160
12-AERO139	2012	Dong Thap	Healthy	Growth	*A. hydrophila*			*A. hydrophila*	96.2878	NF*	251	860*	417*	264	1114	445
40-AH140	2013	Can Tho	Healthy	Growth	*A. hydrophila*			*A. hydrophila*	96.9048	251	210	214	122	211	221	217
41-AH141	2013	An Giang	Healthy	Fingerling	*A. hydrophila*			*A. dhakensis*	99.0412	656	415	164	160	267	457	160
1-AH142	2013	An Giang		Growth	*A. hydrophila*			*A. dhakensis*	99.0509	656	415	164	160	267	457	160
2-AH144	2012	Vinh Long		Water	*A. hydrophila*			*A. dhakensis*	99.0561	656	415	164	160	267	457	160
11-AH145	2013	An Giang		Water	*A. hydrophila*			*A. salmonicida*	97.4951	NF*	123*	116	562	22*	1037	837*
3-AH146	2012	Vinh Long	Diseased	Growth	*A. hydrophila*			*A. hydrophila*	96.9365	251	210	214	122	211	221	217
4-AH147	2012	Can Tho	Diseased	Growth	*A. hydrophila*			*A. hydrophila*	96.8742	251	210	214	122	211	221	217
13-AERO149	2012	Vinh Long	Diseased	Growth	*A. hydrophila*			*A. dhakensis*	99.003	656	415	164	160	267	457	160
14-AERO150	2013	Can Tho	Diseased	Growth	*A. hydrophila*			*A. dhakensis*	99.0719	656	415	164	160	267	457	160
15-AERO153	2014	Tien Giang	Diseased	Growth	*A. hydrophila*			*A. dhakensis*	99.0468	656	415	164	160	267	457	160
16-AERO154	2014	Tien Giang	Diseased	Fingerling	*A. hydrophila*			*A. dhakensis*	99.0462	656	415	164	160	267	457	160
17-AERO155	2014	Tien Giang	Diseased	Fingerling	*A. hydrophila*			*A. dhakensis*	99.0707	656	415	164	160	267	457	160
5-AH157	2015	Can Tho	Healthy	Growth	*A. hydrophila*			*A. dhakensis*	99.029	656	415	164	160	267	457	160
12-AH160	2015	Vinh Long		Water	*A. hydrophila*			*A. caviae*	97.9121	NF*	237	234	690*	991	251	96
13-AH161	2015	Dong Thap	Diseased	Growth	*A. hydrophila*			*A. hydrophila*	96.8967	NF*	380*	710*	833*	842*	780	764*
18-AERO162	2017	An Giang	Healthy	Growth	*A. hydrophila*			*A. dhakensis*	99.0484	656	415	164	160	267	457	160
38-AH163	2018	An Giang	Healthy	Growth	*A. hydrophila*			*A. dhakensis*	99.0452	656	415	164	160	267	457	160
39-AH164	2014	An Giang	Healthy	Growth	*A. hydrophila*			*A. dhakensis*	99.0287	656	415	164	160	267	457	160
15-AH166	2016	Dong Thap	Healthy	Fingerling	*A. hydrophila*			*A. hydrophila*	96.9649	NF*	380*	710*	281*	842*	780	830*
17-AH168	2012	Vinh Long	Diseased	Growth	*A. hydrophila*			*A. dhakensis*	99.039	656	415	164	160	267	457	160
19-AERO170	2012	An Giang	Diseased	Growth	*A. hydrophila*			*A. dhakensis*	99.0644	656	415	164	160	267	457	160
20-AERO171	2018	Vinh Long	Diseased	Growth	*A. hydrophila*			*A. dhakensis*	99.0311	656	415	164	160	267	457	160
21-CXD172	2013	Ben Tre	Diseased	Growth	*A. hydrophila*			*A. dhakensis*	97.2467	NF*?	397	143*	889*	504	581*?	925*
22-AERO173	2013	Ben Tre	Diseased	Growth	*A. hydrophila*			*A. hydrophila*	96.9249	251	210	214	122	211	221	217
32-CXD174	2012	An Giang	Diseased	Fingerling	*A. hydrophila*			*A. veronii*	96.6347	NF*	48*	295*	578*	68*	797*	112*
33-CXD175	2013	Ben Tre		Water	*A. hydrophila*			*A. veronii*	94.4677	NF*?	754*?	545	834	579	720*	650
34-CXD176	2012	An Giang	Healthy	Growth	*A. hydrophila*			*A. caviae*	97.911	NF*	237	234	690*	991	251	96
35-CXD177	2016	An Giang		Water	*A. hydrophila*			*A. dhakensis*	97.2226	NF*	397*	398	654*	465*	284	449*
36-CXD178	2013	Ben Tre	Healthy	Growth	*A. hydrophila*			*A. dhakensis*	97.2293	NF*	675*	772	519	329	289*	579*
37-CXD179	2013	Dong Thap		Water	*A. hydrophila*			*A. veronii*	96.5824	NF	214	226	302	216	124	233
6-AH183	2012	An Giang		Water	*A. hydrophila*			*A. hydrophila*	96.9275	251	210	214	122	211	221	217
38-CXD184	2017	Dong Thap	Healthy	Fingerling	*A. hydrophila*			*A. caviae*	97.9309	NF*	914*	769	690*	664*	249	215*
23-AERO185	2013	Vinh Long		Water	*A. hydrophila*			*A. dhakensis*	99.0175	656	415	164	160	267	457	160
39-CXD186	2012	Vinh Long	Diseased	Growth	*A. hydrophila*			*A. dhakensis*	97.2217	NF*	871	469*	685	527*	1145	586*
9		TP.HCM		Human			*A. hydrophila*	*A. dhakensis*	97.4046	252	211	143	211	212	222	218

15		TP.HCM		Human			*A. hydrophila*	*A. dhakensis*	97.2557	337	259	254	258	260	279	274
10		TP.HCM		Human			*A. hydrophila*	*A. dhakensis*	97.2559	407	305	294	160	306	328	322
21		TP.HCM		Human			*A. hydrophila*	*A. hydrophila*	96.9032	517	381	370	384	377	416	408
4		TP.HCM		Human			*A. hydrophila*	*A. dhakensis*	97.1727	540	258	107	388	266	100	282
13		TP.HCM		Human			*A. hydrophila*	*A. veronii*	96.6218	NF*	223	576	48*	312*	235	87*
14		TP.HCM		Human			*A. hydrophila*	*A. dhakensis*	97.4443	NF*?	400	545*?	293*	259	863*?	898
20		TP.HCM		Human			*A. hydrophila*	*A. dhakensis*	97.2544	NF*?	241*	143	160*	327*	771*	223*?
24		TP.HCM		Human			*A. hydrophila*	*A. dhakensis*	97.3659	NF*?	257*?	93	812*	266	413	586
17		TP.HCM		Human			*A. hydrophila*	*A. hydrophila*	96.9484	NF*?	497	473	511	506	876*?	666*
16		TP.HCM		Human			*A. hydrophila*	*A. hydrophila*	96.9459	NF*?	771*	425*?	458*	367*	817*	793
12		TP.HCM		Human			*A. hydrophila*	*A. veronii*	96.4805	NF*?	68	154	311	148	335*?	330
25		TP.HCM		Human			*A. hydrophila*	*A. dhakensis*	97.4266	1330?	94	103	347	875	946?	913?
11		TP.HCM		Human			*A. hydrophila*	*A. dhakensis*	97.4164	1628*	391	315	238	714	766*	279
1		TP.HCM		Human			*A. hydrophila*	*A. dhakensis*	97.3916	2068?	442	143	967	989	1115?	969
5		TP.HCM		Human			*A. hydrophila*	*A. dhakensis*	97.3854	NF	132	143	236	504	428	242
22		TP.HCM		Human			*A. hydrophila*	*A. dhakensis*	97.3574	NF	241	483	534	400	415	732
2		TP.HCM		Human			*A. hydrophila*	*A. dhakensis*	97.2831	NF	840	254	389	236	1066	969
18		TP.HCM		Human			*A. hydrophila*	*A. dhakensis*	97.3533	NF*?	132	302	347*	378	1038*?	969
6		TP.HCM		Human			*A. hydrophila*	*A. dhakensis*	97.2962	NF*?	132	143	391	381	422	956*?
3		TP.HCM		Human			*A. hydrophila*	*A. dhakensis*	97.2971	NF*?	241	143	431	498*	1151	919*?
8		TP.HCM		Human			*A. hydrophila*	*A. dhakensis*	97.2955	NF*?	384	263	326*?	394*	1006	336*
19		TP.HCM		Human			*A. hydrophila*	*A. dhakensis*	97.4171	NF*	391	484*	405	714*	550*	279
23		TP.HCM		Human			*A. hydrophila*	*A. dhakensis*	97.424	NF*	241*	294	261	695	817*	732
7		TP.HCM		Human			*A. hydrophila*	*A. dhakensis*	97.3718	1330	94	103	347	875	946	913

Table S2. *Aeromonas* assembly accessions used in the paper and the corresponding Project accession.

Sequencing ID	Read Accession number
1	ERS16336231
2	ERS16336232
3	ERS16336233
4	ERS16336234
5	ERS16336235
6	ERS16336236
7	ERS16336237
8	ERS16336238
9	ERS16336239
10	ERS16336240
11	ERS16336241
12	ERS16336242
13	ERS16336243
14	ERS16336244
15	ERS16336245
16	ERS16336246
17	ERS16336247
18	ERS16336248
19	ERS16336249
20	ERS16336250
21	ERS16336251
22	ERS16336252
23	ERS16336253
24	ERS16336254
25	ERS16336255
1-AD01	ERS16336256
1-AH142	ERS16336257
10-AD26	ERS16336258
10-AH34	ERS16336259
10-AH37	ERS16336260
11-AD27	ERS16336261
11-AH145	ERS16336262
11-AH39	ERS16336263
12-AERO139	ERS16336264
12-AH160	ERS16336265
12-AH41	ERS16336266
13-AERO149	ERS16336267
13-AH161	ERS16336268
14-AERO150	ERS16336269

15-AERO153	ERS16336270
15-AH166	ERS16336271
16-AERO154	ERS16336272
17-AERO155	ERS16336273
17-AH168	ERS16336274
18-AD18	ERS16336275
18-AERO162	ERS16336276
19-AD19	ERS16336277
19-AERO170	ERS16336278
2-AH144	ERS16336279
20-AERO171	ERS16336280
20-AH38	ERS16336281
21-AH42	ERS16336282
21-CXD172	ERS16336283
22-AERO173	ERS16336284
22-AH43	ERS16336285
23-AERO185	ERS16336286
23-AH44	ERS16336287
24-AH06	ERS16336288
24-AH100	ERS16336289
25-AH121	ERS16336290
26-AD08	ERS16336291
26-AH122	ERS16336292
27-AH123	ERS16336293
27-AH126	ERS16336294
28-AH124	ERS16336295
28-AH127	ERS16336296
29-AH125	ERS16336297
3-AH03	ERS16336298
3-AH146	ERS16336299
3-AH17	ERS16336300
30-AH129	ERS16336301
30-AH130	ERS16336302
31-AH132	ERS16336303
31-AH40	ERS16336304
32-AD24	ERS16336305
32-CXD174	ERS16336306
33-AH133	ERS16336307
33-CXD175	ERS16336308
34-AH134	ERS16336309
34-CXD176	ERS16336310
35-AH135	ERS16336311
35-CXD177	ERS16336312

36-AH137	ERS16336313
36-CXD178	ERS16336314
37-AH33	ERS16336315
37-CXD179	ERS16336316
38-AH163	ERS16336317
38-CXD184	ERS16336318
39-AH164	ERS16336319
39-CXD186	ERS16336320
4-AD25	ERS16336321
4-AH04	ERS16336322
4-AH147	ERS16336323
40-AH140	ERS16336324
41-AH141	ERS16336325
5-AD32	ERS16336326
5-AH05	ERS16336327
5-AH157	ERS16336328
6-AD9	ERS16336329
6-AH183	ERS16336330
6-AH35	ERS16336331
7-AD16	ERS16336332
8-AD10	ERS16336333
8-AD23	ERS16336334
8-AH22	ERS16336335
9-AD11	ERS16336336
9-AD29	ERS16336337
9-AH36	ERS16336338
AD02	ERS16336339
AD21	ERS16336340
AH07	ERS16336341
AH13	ERS16336342
**Project accession number**	PRJEB65955

Table S3. antibiotic-resistance genes and virulence factors result of *A. hydrophila* and *A. dhakensis*

			Antibiotic resistant genes															Polar/lateral flagella														Type IV pilus										T2SS	T6SS					Toxin and hemolysin						Enzyme								
Sample_ID	ST	Species	aadA16	strAB	blaAQU-1	blaAQU-2	blaAQU-3	blaTEM-105	cepH	imiH	qnrS2	floR	arr2	sull/II	tetA	tetE	dfrA	flaAB	flaG	flaHJ	fleQRS	flgA-N	flhAB	fliA	fliE	fliF-Q	fliH	fliK	fliR	flmDH	maf-2	mshII1JKLMEGF	mshABCDNOPQ	flp1A-L	tapA	tapCDF	tapBM-P	tapQVY1	tapTUW	Tap	Tap A*	exeA-N	qseBC	AhyIR	vasH	vasK	vgrG2	aerA/act	ahh1	dam	ast	alt	lip	rtxA-H	AHA0617	ahpB	eprA1	AHA1741	exu1	vacB	gidA	tagA
1	ST2068	A. dhakensis	0	0	1	0	0	0	0	1	0	0	0	0	0	1	0	0	0	0	0	0	0	0	1	1	0	0	0	1	1	0	0	0	0	0	0	0	1	0	0	0	1	0	0	1	1	0	1	1	0	1	0	0	0	0	0	0	0	1	1	1
2	STNF	A. dhakensis	0	0	1	0	0	0	0	1	0	0	0	0	0	0	0	0	0	0	0	0	0	1	1	1	1	0	0	0	1	0	0	0	0	0	0	0	1	0	0	0	1	1	1	1	1	0	1	1	0	1	0	1	0	0	0	1	0	1	1	1
3	STNF	A. dhakensis	0	0	0	0	1	0	0	1	0	0	0	0	0	0	0	0	0	0	0	0	1	1	1	1	1	0	0	1	1	0	0	0	0	1	0	0	1	0	0	0	1	1	1	1	1	0	1	1	0	1	0	1	1	0	0	1	0	1	1	1
4	ST540	A. dhakensis	0	0	0	1	0	0	0	0	0	0	0	0	0	0	0	0	0	0	1	0	1	1	1	1	1	0	1	1	1	0	0	0	0	1	0	0	1	0	0	0	1	1	1	1	1	0	0	1	0	1	0	0	0	0	0	1	0	1	1	1
5	STNF	A. dhakensis	0	0	1	0	0	0	0	0	0	0	0	1	1	0	1	0	0	0	1	0	1	1	1	1	0	0	1	1	1	0	0	0	0	1	0	0	1	0	0	1	1	1	1	1	1	0	1	1	0	1	0	1	1	0	0	0	0	1	1	1
6	STNF	A. dhakensis	0	0	0	0	1	0	0	1	0	0	0	0	0	0	0	0	0	0	1	0	1	1	1	1	1	0	1	1	1	0	0	0	1	1	0	0	1	0	0	0	1	1	1	1	1	1	1	1	1	1	0	0	1	0	0	1	0	1	1	1
7	ST1330	A. dhakensis	0	0	0	1	0	0	0	1	0	0	0	0	0	1	0	0	0	0	0	0	1	1	1	1	0	0	1	1	1	0	0	0	0	1	0	0	1	0	0	0	1	1	0	0	0	0	1	1	0	1	0	1	0	0	0	1	0	1	1	1
8	STNF	A. dhakensis	0	0	1	0	0	0	0	1	0	0	0	0	0	0	0	0	0	0	0	0	0	1	1	1	0	0	0	1	1	0	0	0	0	1	0	0	1	0	0	0	1	1	1	1	1	0	1	1	0	1	0	1	1	0	0	1	0	0	1	1
9	ST252	A. dhakensis	0	0	0	0	1	0	0	1	0	0	0	0	0	0	0	0	0	0	1	0	1	1	1	1	1	0	1	1	1	0	0	0	0	1	0	0	1	0	0	0	1	1	1	1	1	0	1	1	0	1	0	1	1	0	0	1	0	1	1	1
10	ST407	A. dhakensis	0	0	0	0	1	0	0	1	0	0	0	0	0	0	0	0	0	0	1	0	1	0	1	1	1	0	1	1	1	0	0	0	1	1	0	0	1	0	0	1	1	1	0	1	1	1	1	1	0	1	0	1	1	0	0	1	0	1	1	1
11	ST1628	A. dhakensis	0	0	1	0	0	0	0	1	0	0	0	0	0	0	0	0	0	0	1	0	1	1	1	1	1	0	1	1	1	0	0	0	0	1	0	0	1	0	0	1	1	1	1	1	1	0	1	1	0	1	0	1	1	0	0	1	0	1	1	1
14	STNF	A. dhakensis	0	0	0	0	1	0	1	0	0	0	0	0	0	0	0	0	0	0	1	0	1	1	1	1	0	0	1	1	1	0	0	0	0	1	0	0	1	0	0	1	1	1	1	1	1	0	1	1	0	1	0	1	1	0	0	1	0	1	1	1
15	ST337	A. dhakensis	0	0	1	0	0	0	0	1	0	0	0	0	0	1	0	0	0	0	0	0	1	1	0	1	1	0	1	1	1	0	0	0	0	1	0	0	1	0	0	1	1	1	1	1	1	0	1	1	0	1	0	1	1	0	1	1	0	1	1	1
16	STNF	A. hydrophila	0	0	0	0	0	0	1	1	0	0	0	0	0	0	0	0	1	0	1	1	1	1	1	1	1	0	1	1	0	1	0	0	0	1	1	1	1	0	1	0	0	1	0	1	0	0	1	0	1	0	0	0	1	0	0	1	0	0	0	0
17	STNF	A. hydrophila	1	1	0	0	0	1	0	1	0	1	1	1	1	0	1	0	1	0	1	1	1	1	1	1	1	1	1	1	0	0	0	0	0	1	1	1	1	0	1	1	1	1	1	1	1	0	1	1	1	1	0	0	1	0	0	1	1	1	1	1
18	STNF	A. dhakensis	0	0	0	0	1	0	0	1	0	0	0	0	0	0	0	0	0	0	1	0	1	1	1	1	1	0	1	1	1	0	0	0	1	1	0	0	1	0	0	0	1	1	1	1	1	1	1	1	0	1	0	1	1	0	0	1	0	1	1	1
19	STNF	A. dhakensis	0	0	0	0	1	0	0	1	0	0	0	0	0	0	0	0	0	0	1	1	1	1	1	1	0	0	0	1	1	0	0	0	0	1	0	0	1	0	0	0	1	1	1	1	1	0	1	1	0	1	0	1	0	0	0	1	0	1	1	1
20	STNF	A. dhakensis	0	0	1	0	0	0	0	1	0	0	0	0	0	0	0	0	0	0	0	0	1	0	1	1	1	0	1	1	1	0	0	0	0	1	0	0	1	0	0	0	1	1	1	1	1	1	1	1	0	1	0	1	0	0	0	1	0	1	1	1
21	ST517	A. hydrophila	0	0	0	0	0	0	0	1	1	0	0	0	1	0	0	0	1	1	1	1	1	1	1	1	1	1	1	1	0	1	1	0	0	1	1	1	1	0	1	1	1	1	0	0	1	0	1	1	1	1	0	0	1	0	0	1	0	1	1	1
22	STNF	A. dhakensis	0	0	1	0	0	0	0	1	0	0	0	0	0	0	0	0	0	0	0	0	1	1	0	1	1	0	1	1	1	0	0	0	1	1	0	0	1	0	0	0	1	1	0	1	1	1	1	1	0	1	0	1	1	0	0	1	0	1	1	1
23	STNF	A. dhakensis	0	0	0	0	1	0	0	0	0	0	0	0	0	0	0	0	0	0	0	0	1	1	1	1	1	0	1	1	1	0	0	0	0	1	0	0	1	0	0	1	1	1	1	1	1	1	1	1	0	1	0	1	0	0	0	1	0	1	1	1
24	STNF	A. dhakensis	0	0	0	0	1	0	0	1	0	0	0	0	0	0	0	0	0	0	0	0	0	1	1	1	0	0	0	1	1	0	0	0	0	1	0	0	1	0	0	0	1	1	1	1	1	1	1	1	0	1	0	1	1	0	0	1	0	1	1	1
25	ST1330	A. dhakensis	0	0	0	1	0	0	0	1	0	0	0	0	0	0	0	0	0	0	0	0	0	0	1	1	0	0	1	0	1	0	0	0	0	0	0	0	1	0	0	0	1	1	0	1	0	0	1	1	0	1	0	1	0	0	0	0	0	1	1	1
10-AD26	ST251	A. hydrophila	0	0	0	0	0	0	1	0	0	0	1	1	1	0	1	1	1	1	1	1	1	1	1	1	1	1	1	1	0	1	1	1	1	1	1	1	1	1	1	1	1	1	1	1	1	1	1	1	1	1	1	1	1	1	1	1	1	1	1	1
10-AH34	ST251	A. hydrophila	0	0	0	0	0	0	1	0	0	0	1	1	0	0	1	1	1	1	1	1	1	1	1	1	1	1	1	1	0	1	1	1	1	1	1	1	1	1	1	1	1	1	1	1	1	1	1	1	1	1	1	1	1	1	1	1	1	1	1	1
10-AH37	ST656	A. dhakensis	0	0	0	0	1	0	0	1	0	0	0	0	0	0	0	0	0	0	1	1	1	1	1	1	1	0	1	1	1	0	0	0	1	1	0	1	1	0	0	1	1	1	1	1	1	1	1	1	0	1	1	1	1	1	1	1	1	1	1	1
11-AD27	ST656	A. dhakensis	0	0	0	0	1	0	0	1	1	1	0	1	1	0	1	0	0	0	1	1	1	1	1	1	1	0	1	1	1	0	0	0	1	1	0	1	1	0	0	1	1	1	1	1	1	1	1	1	0	1	1	1	1	1	1	1	1	1	1	1
11-AH39	ST656	A. dhakensis	0	0	0	0	1	0	0	1	0	0	0	0	0	0	0	0	0	0	1	1	1	1	1	1	1	0	1	1	1	0	0	0	1	1	0	1	1	0	0	1	1	1	1	1	1	1	1	1	0	1	1	1	1	1	1	1	1	1	1	1
12-AERO139	STNF	A. hydrophila	0	0	0	0	0	0	1	1	0	0	0	0	0	0	0	0	1	1	1	1	1	1	1	1	1	1	1	1	1	1	0	1	0	1	1	1	1	0	1	1	1	1	1	1	1	1	1	1	1	1	1	1	1	1	1	1	1	1	1	1
12-AH41	ST656	A. dhakensis	0	0	0	0	1	0	0	1	0	0	0	0	0	0	0	0	0	0	1	1	1	1	1	1	1	0	1	1	1	0	0	0	1	1	0	1	1	0	0	1	1	1	1	1	1	1	1	1	0	1	1	1	1	1	1	1	1	1	1	1
13-AERO149	ST656	A. dhakensis	0	0	0	0	1	0	0	1	0	0	0	0	0	0	0	0	0	0	1	1	1	1	1	1	1	0	1	1	1	0	0	0	1	1	0	1	1	0	0	1	1	1	1	1	1	1	1	1	0	1	1	1	1	1	1	1	1	1	1	1
13-AH161	Novel ST	A. hydrophila	0	1	0	0	0	0	1	1	0	1	0	1	1	0	0	0	1	1	1	1	1	1	1	1	1	1	1	1	1	1	0	0	0	1	1	1	1	0	1	1	1	1	1	1	1	0	1	1	0	1	1	1	1	1	1	1	1	1	1	1
14-AERO150	ST656	A. dhakensis	0	0	0	0	1	0	0	1	1	0	0	1	1	0	1	0	0	0	1	1	1	1	1	1	1	0	1	1	1	0	0	0	1	1	0	1	1	0	0	1	1	1	1	1	1	1	1	1	0	1	1	1	1	1	1	1	1	1	1	1
15-AERO153	ST656	A. dhakensis	0	0	0	0	1	0	0	1	1	0	0	1	1	0	1	0	0	0	1	1	1	1	1	1	1	0	1	1	1	0	0	0	1	1	0	1	1	0	0	1	1	1	1	1	1	1	1	1	0	1	1	1	1	1	1	1	1	1	1	1
15-AH166	Novel ST	A. hydrophila	0	1	0	0	0	0	1	1	0	1	0	1	1	0	0	0	1	1	1	1	1	1	1	1	1	1	1	1	1	1	0	0	0	1	1	1	1	0	1	1	1	1	1	1	1	0	1	1	0	1	1	1	1	1	1	1	1	1	1	1
16-AERO154	ST656	A. dhakensis	0	0	0	0	1	0	0	1	1	0	0	1	1	0	1	0	0	0	1	1	1	1	1	1	1	0	1	1	1	0	0	0	1	1	0	1	1	0	0	1	1	1	1	1	1	1	1	1	0	1	1	1	1	1	1	1	1	1	1	1
17-AERO155	ST656	A. dhakensis	0	0	0	0	1	0	0	1	1	0	0	1	1	0	1	0	0	0	1	1	1	1	1	1	1	0	1	1	1	0	0	0	1	1	0	1	1	0	0	1	1	1	1	1	1	1	1	1	0	1	1	1	1	1	1	1	1	1	1	1
17-AH168	ST656	A. dhakensis	0	0	0	0	1	0	0	1	1	0	0	1	1	0	1	0	0	0	1	1	1	1	1	1	1	0	1	1	1	0	0	0	1	1	0	1	1	0	0	1	1	1	1	1	1	1	1	1	0	1	1	1	1	1	1	1	1	1	1	1
18-AD18	ST656	A. dhakensis	0	0	0	0	1	0	0	1	0	0	1	1	0	0	1	0	0	0	1	1	1	1	1	1	1	0	1	1	1	0	0	0	1	1	0	1	1	0	0	1	1	1	1	1	1	1	1	1	0	1	1	1	1	1	1	1	1	1	1	1
18-AERO162	ST656	A. dhakensis	0	0	0	0	1	0	0	1	1	0	0	0	0	0	0	0	0	0	1	1	1	1	1	1	1	0	1	1	1	0	0	0	1	1	0	1	1	0	0	1	1	1	1	1	1	1	1	1	0	1	1	1	1	1	1	1	1	1	1	1
19-AD19	STNF	A. dhakensis	0	0	1	0	0	0	0	1	0	0	0	0	0	0	0	0	1	0	1	1	1	1	1	1	1	0	1	1	1	0	0	0	0	1	0	1	1	0	0	1	1	1	1	1	1	0	1	1	0	1	1	1	1	1	1	1	1	1	1	1
19-AERO170	ST656	A. dhakensis	0	0	0	0	1	0	0	1	1	1	0	1	1	0	1	0	0	0	1	1	1	1	1	1	1	0	1	1	1	0	0	0	1	1	0	1	1	0	0	1	1	1	1	1	1	1	1	1	0	1	1	1	1	1	1	1	1	1	1	1
1-AD01	ST656	**A. dhakensis**	0	0	0	0	1	0	0	1	1	0	0	0	0	0	0	0	1	0	1	1	1	1	1	1	1	0	1	1	1	0	0	0	1	1	0	1	1	0	0	1	1	1	1	1	1	1	1	1	0	1	1	1	1	1	1	1	1	1	1	1
1-AH142	ST656	A. dhakensis	0	0	0	0	1	0	0	1	1	0	0	0	0	0	0	0	0	0	1	1	1	1	1	1	1	0	1	1	1	0	0	0	1	1	0	1	1	0	0	1	1	1	1	1	1	1	1	1	0	1	1	1	1	1	1	1	1	1	1	1
20-AERO171	ST656	A. dhakensis	0	0	0	0	1	0	0	1	1	1	0	1	1	0	1	0	0	0	1	1	1	1	1	1	1	0	1	1	1	0	0	0	1	1	0	1	1	0	0	1	1	1	1	1	1	1	1	1	0	1	1	1	1	1	1	1	1	1	1	1
20-AH38	ST656	A. dhakensis	0	0	0	0	1	0	0	1	0	0	0	0	0	0	0	0	0	0	1	1	1	1	1	1	1	0	1	1	1	0	0	0	1	1	0	1	1	0	0	1	1	1	1	1	1	1	1	1	0	1	1	1	1	1	1	1	1	1	1	1
21-AH42	ST656	A. dhakensis	0	0	0	0	1	0	0	1	0	0	0	0	0	0	0	0	0	0	1	1	1	1	1	1	1	0	1	1	1	0	0	0	1	1	0	1	1	0	0	1	1	1	1	1	1	1	1	1	0	1	1	1	1	1	1	1	1	1	1	1
21-CXD172	STNF	A. dhakensis	0	0	1	0	0	0	0	1	0	0	0	0	0	0	0	0	1	0	1	1	1	1	0	1	1	1	1	1	1	0	0	0	0	1	0	1	1	0	0	1	1	1	1	1	1	0	1	1	0	1	1	1	1	1	1	1	1	1	1	1
22-AERO173	ST251	A. hydrophila	0	0	0	0	0	0	1	0	0	0	1	1	0	0	1	1	1	1	1	1	1	1	1	1	1	1	1	1	0	1	1	1	1	1	1	1	1	1	1	1	1	1	1	1	1	1	1	1	1	1	1	1	1	1	1	1	1	1	1	1
22-AH43	ST656	A. dhakensis	0	0	0	0	1	0	0	1	0	0	0	0	0	0	0	0	0	0	1	1	1	1	1	1	1	0	1	1	1	0	0	0	1	1	0	1	1	0	0	1	1	1	1	1	1	1	1	1	0	1	1	1	1	1	1	1	1	1	1	1
23-AERO185	ST656	A. dhakensis	0	0	0	0	1	0	0	1	0	0	0	0	0	0	0	0	0	0	1	1	1	1	1	1	1	0	1	1	1	0	0	0	1	1	0	1	1	0	0	1	1	1	1	1	1	1	1	1	0	1	1	1	1	1	1	1	1	1	1	1
23-AH44	ST656	A. dhakensis	0	0	0	0	1	0	0	1	0	0	0	0	0	0	0	0	0	0	1	1	1	1	1	1	1	0	1	1	1	0	0	0	1	1	0	1	1	0	0	1	1	1	1	1	1	1	1	1	0	1	1	1	1	1	1	1	1	1	1	1
24-AH06	ST251	A. hydrophila	0	0	0	0	0	0	1	0	0	0	1	1	0	0	1	1	1	1	1	1	1	1	1	1	1	1	1	1	0	1	1	1	1	1	1	1	1	1	1	1	1	1	1	1	1	1	1	1	1	1	1	1	1	0	1	1	1	1	1	1
24-AH100	ST656	A. dhakensis	0	0	0	0	1	0	0	1	1	0	0	0	0	0	0	0	0	0	1	1	1	1	1	1	1	0	1	1	1	0	0	0	1	1	0	1	1	0	0	1	1	1	1	1	1	1	1	1	0	1	1	1	1	1	1	1	1	1	1	1
26-AD08	ST656	A. dhakensis	0	0	0	0	1	0	0	1	1	1	0	1	1	0	1	0	0	0	1	1	1	1	1	1	1	0	1	1	1	0	0	0	1	1	0	1	1	0	0	1	1	1	1	1	1	1	1	1	0	1	1	1	1	1	1	1	1	1	1	1
2-AH144	ST656	A. dhakensis	0	0	0	0	1	0	0	1	1	1	0	1	1	0	1	0	0	0	1	1	1	1	1	1	1	0	1	1	1	0	0	0	1	1	0	1	1	0	0	1	1	1	1	1	1	1	1	1	0	1	1	1	1	1	1	1	1	1	1	1
31-AH132	ST251	A. hydrophila	0	0	0	0	0	0	1	0	1	0	0	0	0	0	0	1	1	1	1	1	1	1	1	1	1	1	1	1	0	1	1	1	1	1	1	1	1	1	1	1	1	1	1	1	1	1	1	1	1	1	1	1	1	1	1	1	1	1	1	1
31-AH40	STNF	A. hydrophila	0	0	0	0	0	0	1	0	0	0	0	0	0	1	0	0	1	1	1	1	1	1	1	1	1	1	1	1	1	1	0	1	0	1	1	1	1	0	1	1	1	1	1	1	1	1	1	1	1	1	1	1	1	0	1	1	1	1	1	1
32-AD24	ST656	A. dhakensis	0	0	0	0	1	0	0	1	1	0	0	0	0	0	0	0	0	0	1	1	1	1	1	1	1	0	1	1	1	0	0	0	1	1	0	1	1	0	0	1	1	1	1	1	1	1	1	1	0	1	1	1	1	1	1	1	1	1	1	1
33-AH133	STNF	A. dhakensis	1	0	0	1	0	0	0	1	0	1	0	0	0	0	1	0	0	0	1	1	1	1	1	1	1	0	1	1	1	0	0	0	0	1	0	1	1	0	0	1	1	1	0	0	1	1	1	1	0	1	1	1	1	1	1	1	1	1	1	1
34-AH134	STNF	A. dhakensis	0	0	0	1	0	0	0	1	0	0	0	0	0	1	0	0	1	0	1	1	1	1	1	1	1	0	1	1	1	0	0	0	0	1	0	1	1	0	0	1	1	1	0	0	1	1	1	1	0	1	1	1	1	1	1	1	1	1	1	1
35-AH135	STNF	A. hydrophila	0	0	0	0	0	0	1	0	0	0	0	0	0	0	0	0	1	1	1	1	1	1	1	1	1	1	1	1	1	1	0	1	0	1	1	1	1	0	1	1	1	1	0	0	1	0	1	1	1	1	1	1	1	1	1	1	1	1	1	1
35-CXD177	STNF	A. dhakensis	0	0	1	0	0	0	0	1	0	0	0	0	0	0	0	0	1	0	1	1	1	1	1	1	1	0	1	1	1	0	0	0	0	1	0	1	1	0	0	1	1	1	1	1	1	0	1	1	1	1	1	0	1	1	1	1	1	1	1	1
36-AH137	ST656	A. dhakensis	0	0	0	0	1	0	0	1	0	0	0	0	0	0	0	0	0	0	1	1	1	1	1	1	1	0	1	1	1	0	0	0	1	1	0	1	1	0	0	1	1	1	1	1	1	1	1	1	0	1	1	1	1	1	1	1	1	1	1	1
36-CXD178	STNF	A. dhakensis	0	0	1	0	0	0	0	1	0	0	0	0	0	0	0	0	1	0	1	1	1	1	1	1	1	0	1	1	1	0	0	0	0	1	0	1	1	0	0	1	1	1	1	1	1	0	1	1	0	1	1	1	1	1	1	1	1	1	1	1
37-AH33	ST251	A. hydrophila	0	0	0	0	0	0	1	0	0	0	1	1	1	0	1	1	1	1	1	1	1	1	1	1	1	1	1	1	0	1	1	1	1	1	1	1	1	1	1	1	1	1	1	1	1	1	1	1	1	1	1	1	1	1	1	1	1	1	1	1
38-AH163	ST656	A. dhakensis	0	0	0	0	1	0	0	1	1	0	0	0	0	0	0	0	0	0	1	1	1	1	1	1	1	0	1	1	1	0	0	0	1	1	0	1	1	0	0	1	1	1	1	1	1	1	1	1	0	1	1	1	1	1	1	1	1	1	1	1
39-AH164	ST656	A. dhakensis	0	0	0	0	1	0	0	1	1	0	0	0	0	0	0	0	0	0	1	1	1	1	1	1	1	0	1	1	1	0	0	0	1	1	0	1	1	0	0	1	1	1	1	1	1	1	1	1	0	1	1	1	1	1	1	1	1	1	1	1
39-CXD186	STNF	A. dhakensis	0	0	1	0	0	0	0	1	0	0	0	0	0	0	0	0	1	0	1	1	1	1	1	1	1	0	1	1	1	0	0	0	0	1	0	1	1	0	0	1	1	1	1	1	1	0	1	1	0	1	1	1	1	1	1	1	1	1	1	1
3-AH03	ST251	A. hydrophila	0	0	0	0	0	0	1	0	0	0	1	1	0	0	1	1	1	1	1	1	1	1	1	1	1	1	1	1	0	1	1	1	1	1	1	1	1	1	1	1	1	1	1	1	1	1	1	1	1	1	1	1	1	1	1	1	1	1	1	1
3-AH146	ST251	A. hydrophila	0	0	0	0	0	0	1	0	0	0	1	1	1	0	1	1	1	1	1	1	1	1	1	1	1	1	1	1	0	1	1	1	1	1	1	1	1	1	1	1	1	1	1	1	1	1	1	1	1	1	1	1	1	1	1	1	1	1	1	1
3-AH17	ST251	A. hydrophila	0	0	0	0	0	0	1	0	0	0	1	1	0	0	1	1	1	1	1	1	1	1	1	1	1	1	1	1	0	1	1	1	1	1	1	1	1	1	1	1	1	1	1	1	1	1	1	1	1	1	1	1	1	1	1	1	1	1	1	1
40-AH140	ST251	A. hydrophila	0	0	0	0	0	0	1	0	0	0	1	1	0	0	1	1	1	1	1	1	1	1	1	1	1	1	1	1	0	1	1	1	1	1	1	1	1	1	1	1	1	1	1	1	1	1	1	1	1	1	1	1	1	1	1	1	1	1	1	1
41-AH141	ST656	A. dhakensis	0	0	0	0	1	0	0	1	1	0	0	0	0	0	0	0	1	0	1	1	1	1	1	1	1	0	1	1	1	0	0	0	1	1	0	1	1	0	0	1	1	1	1	1	1	1	1	1	0	1	1	1	1	1	1	1	1	1	1	1
4-AD25	ST656	A. dhakensis	0	0	0	0	1	0	0	1	1	0	0	0	0	0	0	0	0	0	1	1	1	1	1	1	1	0	1	1	1	0	0	0	1	1	0	1	1	0	0	1	1	1	1	1	1	1	1	1	0	1	1	1	1	1	1	1	1	1	1	1
4-AH04	ST251	A. hydrophila	0	0	0	0	0	0	1	0	0	0	1	1	0	0	1	1	1	1	1	1	1	1	1	1	1	1	1	1	0	1	1	1	1	1	1	1	1	1	1	1	1	1	1	1	1	1	1	1	1	1	1	1	1	1	1	1	1	1	1	1
4-AH147	ST251	A. hydrophila	0	0	0	0	0	0	1	0	0	0	1	1	1	0	1	1	1	1	1	1	1	1	1	1	1	1	1	1	0	1	1	1	1	1	1	1	1	1	1	1	1	1	1	1	1	1	1	1	1	1	1	1	1	1	1	1	1	1	1	1
5-AD32	ST251	A. hydrophila	0	0	0	0	0	0	1	0	0	0	1	1	1	0	1	1	1	1	1	1	1	1	1	1	1	1	1	1	0	1	1	1	1	1	1	1	1	1	1	1	1	1	1	1	1	1	1	1	1	1	1	1	1	1	1	1	1	1	1	1
5-AH05	ST251	A. hydrophila	0	0	0	0	0	0	1	0	0	0	1	1	0	0	1	1	1	1	1	1	1	1	1	1	1	1	1	1	0	1	1	1	1	1	1	1	1	1	1	1	1	1	1	1	1	1	1	1	1	1	1	1	1	1	1	1	1	1	1	1
5-AH157	ST656	A. dhakensis	0	0	0	0	1	0	0	1	0	0	1	1	0	0	1	0	0	0	1	1	1	1	1	1	1	0	1	1	1	0	0	0	1	1	0	1	1	0	0	1	1	1	1	1	1	1	1	1	0	1	1	1	1	1	1	1	1	1	1	1
6-AD9	ST656	A. dhakensis	0	0	0	0	1	0	0	1	1	1	0	1	1	0	1	0	0	0	1	1	1	1	1	1	1	0	1	1	1	0	0	0	1	1	0	1	1	0	0	1	1	1	1	1	1	1	1	1	0	1	1	1	1	1	1	1	1	1	1	1
6-AH183	ST251	A. hydrophila	0	0	0	0	0	0	1	0	0	0	0	0	0	0	0	1	1	1	1	1	1	1	1	1	1	1	1	1	0	1	1	1	1	1	1	1	1	1	1	1	1	1	1	1	1	1	1	1	1	1	1	1	1	1	1	1	1	1	1	1
7-AD16	ST656	A. dhakensis	0	0	0	0	1	0	0	1	1	1	0	1	1	0	1	0	0	0	1	1	1	1	1	1	1	0	1	1	1	0	0	0	1	1	0	1	1	0	0	1	1	1	1	1	1	1	1	1	0	1	1	1	1	1	1	1	1	1	1	1
8-AD10	STNF	A. dhakensis	0	0	0	0	0	0	0	1	0	0	0	0	0	0	0	0	0	0	1	1	1	1	1	1	1	0	1	1	1	0	0	0	1	1	0	1	1	0	0	1	1	1	1	1	1	1	1	1	0	1	1	1	1	1	1	1	1	1	1	1
8-AD23	ST656	A. dhakensis	0	0	0	0	1	0	0	1	1	1	0	1	1	0	1	0	1	0	1	1	1	1	1	1	1	0	1	1	1	0	0	0	1	1	0	1	1	0	0	1	1	1	1	1	1	1	1	1	0	1	1	1	1	1	1	1	1	1	1	1
8-AH22	ST251	A. hydrophila	0	0	0	0	0	0	1	0	0	0	1	1	0	0	1	1	1	1	1	1	1	1	1	1	1	1	1	1	0	1	1	1	1	1	1	1	1	1	1	1	1	1	1	1	1	1	1	1	1	1	1	1	1	1	1	1	1	1	1	1
9-AD11	STNF	A. dhakensis	0	0	0	1	0	0	0	1	0	0	0	0	0	0	0	0	1	0	1	1	1	1	1	1	1	0	1	1	1	0	0	0	0	1	0	1	1	0	0	1	1	1	1	1	1	0	1	1	0	1	1	1	1	1	1	1	1	1	1	1
9-AD29	ST656	A. dhakensis	0	0	0	0	1	0	0	1	1	0	0	1	1	0	1	0	0	0	1	1	1	1	1	1	1	0	1	1	1	0	0	0	1	1	0	1	1	0	0	1	1	1	1	1	1	1	1	1	0	1	1	1	1	1	1	1	1	1	1	1
9-AH36	ST656	A. dhakensis	0	0	0	0	1	0	0	1	0	0	0	0	0	0	0	0	0	0	1	1	1	1	1	1	1	0	1	1	1	0	0	0	1	1	0	1	1	0	0	1	1	1	1	1	1	1	1	1	0	1	1	1	1	1	1	1	1	1	1	1
AD02	ST656	A. dhakensis	0	0	0	0	1	0	0	1	1	1	0	1	1	0	1	0	0	0	1	1	1	1	1	1	1	0	1	1	1	0	0	0	1	1	0	1	1	0	0	1	1	1	1	1	1	1	1	1	0	1	1	1	1	1	1	1	1	1	1	1
AD21	ST656	A. dhakensis	0	0	0	0	1	0	0	1	1	0	0	1	1	0	1	0	0	0	1	1	1	1	1	1	1	0	1	1	1	0	0	0	1	1	0	1	1	0	0	1	1	1	1	1	1	1	1	1	0	1	1	1	1	1	1	1	1	1	1	1
AH07	ST251	A. hydrophila	0	0	0	0	0	0	1	0	0	0	0	0	0	0	0	1	1	1	1	1	1	1	1	1	1	1	1	1	0	1	1	1	1	1	1	1	1	1	1	1	1	1	1	1	1	1	1	1	1	1	1	1	1	1	1	1	1	1	1	1
AH13	ST251	A. hydrophila	0	0	0	0	0	0	1	0	0	0	0	0	0	0	0	1	1	1	1	1	1	1	1	1	1	1	1	1	0	1	1	1	1	1	1	1	1	1	1	1	1	1	1	1	1	1	1	1	1	1	1	1	1	1	1	1	1	1	1	1

Table S4. Functional annotation and cellular localization predictions of core protein between *A. hydrophila* ST251 and *A. dhakensis* ST656

Gene	COG_DESCRIPTION	CELLO_location	Discarded by DeepTMHMM	Min len	Max len
group_6501	Transcription	OuterMembrane	Cytoplasm	1070	1070
flgB	Cell motility	Extracellular	Cytoplasm	398	398
cheR-3	Signal transduction mechanisms	OuterMembrane	Cytoplasm	824	824
flgM	Transcription	Extracellular	Cytoplasm	320	320
ATP-dependent RNA helicase	Replication, recombination and repair	OuterMembrane	Cytoplasm	1376	1376
group_1326	Secondary metabolites biosynthesis, transport and catabolism	Extracellular	Periplasm	488	10688
msrB	Posttranslational modification, protein turnover, chaperones	Extracellular	Cytoplasm	392	392
Putative glucose-6-phosphate 1-epimerase	Carbohydrate transport and metabolism	Extracellular	Cytoplasm	866	866
peptide-methionine (S)-S-oxide reductase MsrA	Posttranslational modification, protein turnover, chaperones	Extracellular	Cytoplasm	632	632
Nitroreductase domain-containing protein	Energy production and conversion	OuterMembrane	Cytoplasm	725	725
fbp	Carbohydrate transport and metabolism	OuterMembrane	Cytoplasm	1034	1034
Prolyl endopeptidase	Amino acid transport and metabolism	OuterMembrane		2147	2147
Sucrose porin	Carbohydrate transport and metabolism	OuterMembrane		1484	1484
Alpha-amylase	Carbohydrate transport and metabolism	Extracellular		1397	1406
group_6692	Carbohydrate transport and metabolism	OuterMembrane		2381	2381
group_903	Cell cycle control, cell division, chromosome partitioning	OuterMembrane		1340	1349
fliF	Cell motility	OuterMembrane		1712	1712
tppA	Cell motility	Extracellular		401	401
tapY1	Cell motility	OuterMembrane		3353	3356
Pilus assembly protein	Cell motility	OuterMembrane		476	476
Putative type IV pilin	Cell motility	OuterMembrane		1094	1094
flgL	Cell motility	Extracellular		1208	1208
flgK	Cell motility	Extracellular		845	2000
flgI	Cell motility	OuterMembrane		1097	1097
flgH	Cell motility	Extracellular		671	671
flgG	Cell motility	Extracellular		788	788
flgE	Cell motility	Extracellular		1334	1334
flgD	Cell motility	Extracellular		728	728
Flagella basal body P-ring formation protein FlgA	Cell motility	OuterMembrane		683	683
fimC	Cell motility	OuterMembrane		2534	2534
mshA	Cell motility	OuterMembrane		671	671
Transgly domain-containing protein	Cell wall/membrane/envelope biogenesis	OuterMembrane		3083	3083
TPR_REGION domain-containing protein	Cell wall/membrane/envelope biogenesis	OuterMembrane		1106	1106
adeA	Cell wall/membrane/envelope biogenesis	OuterMembrane		1193	1193

bamB	Cell wall/membrane/envelope biogenesis	OuterMembrane		1184	1184
D-alanyl-D-alanine endopeptidase	Cell wall/membrane/envelope biogenesis	OuterMembrane		920	920
Outer membrane protein	Cell wall/membrane/envelope biogenesis	OuterMembrane		1991	1991
Type I secretion outer membrane protein	Cell wall/membrane/envelope biogenesis	OuterMembrane		1328	1328
bamA	Cell wall/membrane/envelope biogenesis	OuterMembrane		2423	2423
porin	Cell wall/membrane/envelope biogenesis	OuterMembrane		1067	1067
group_5747	Cell wall/membrane/envelope biogenesis	Extracellular		476	476
lptD	Cell wall/membrane/envelope biogenesis	OuterMembrane		2432	2432
group_6167	Cell wall/membrane/envelope biogenesis	OuterMembrane		1049	1049
Protease LasA	Cell wall/membrane/envelope biogenesis	Extracellular		347	1163
group_1412	Cell wall/membrane/envelope biogenesis	Extracellular		656	656
motY	Cell wall/membrane/envelope biogenesis	OuterMembrane		875	875
Outer membrane protein OmpK	Cell wall/membrane/envelope biogenesis	OuterMembrane		842	842
Peptidase P60	Cell wall/membrane/envelope biogenesis	OuterMembrane		449	449
group_6384	Cell wall/membrane/envelope biogenesis	Extracellular		1103	1103
Autotransporter assembly factor TamA	Cell wall/membrane/envelope biogenesis	OuterMembrane		1658	1658
Porin	Cell wall/membrane/envelope biogenesis	OuterMembrane		1064	1064
LemA family protein	Cell wall/membrane/envelope biogenesis	OuterMembrane		590	590
Outer membrane efflux protein	Cell wall/membrane/envelope biogenesis	OuterMembrane		1406	1406
TIGR02099 family protein	Function unknown	OuterMembrane		3863	3872
DUF1425 domain-containing protein	Function unknown	OuterMembrane		398	398
group_6414	Function unknown	OuterMembrane		1421	1433
Serine protease	General function prediction only	OuterMembrane		2258	2258
Superoxide dismutase	Inorganic ion transport and metabolism	Extracellular		581	584
Ligand-gated channel protein	Inorganic ion transport and metabolism	OuterMembrane		2126	2162
Ferrichrome-iron receptor	Inorganic ion transport and metabolism	OuterMembrane		2144	2144
group_1518	Inorganic ion transport and metabolism	Extracellular		1394	1406
TonB-dependent copper receptor	Inorganic ion transport and metabolism	OuterMembrane		1967	1967
TonB-dependent siderophore receptor	Inorganic ion transport and metabolism	OuterMembrane		2042	2042
group_1164	Inorganic ion transport and metabolism	OuterMembrane		2093	2093
siderophore amonabactin TonB-dependent receptor	Inorganic ion transport and metabolism	OuterMembrane		1973	1973
tapQ	Intracellular trafficking, secretion, and vesicular transport	OuterMembrane		2201	2201
exeD	Intracellular trafficking, secretion, and vesicular transport	OuterMembrane		2036	2036
Domain of Unknown Function (DUF748)	Intracellular trafficking, secretion, and vesicular transport	OuterMembrane		2051	2900
rtxA	Lipid transport and metabolism	OuterMembrane		13319	13979
Long-chain fatty acid transporter	Lipid transport and metabolism	OuterMembrane		1298	1298

Long-chain fatty acid transport protein	Lipid transport and metabolism	OuterMembrane		1256	1256
degS	Posttranslational modification, protein turnover, chaperones	OuterMembrane		1121	1121
Tricorn protease homolog	Posttranslational modification, protein turnover, chaperones	OuterMembrane		1817	3170
Microbial serine proteinase	Posttranslational modification, protein turnover, chaperones	OuterMembrane		1874	1874
Aculeacin A acylase	Secondary metabolites biosynthesis, transport and catabolism	OuterMembrane		365	2504
group_1450	Unknown protein	Extracellular		476	476
group_219	Unknown protein	OuterMembrane		335	902
Beta/gamma crystallin 'Greek key' domain-containing protein	Unknown protein	OuterMembrane		542	542
group_873	Unknown protein	OuterMembrane		506	506
AsmA_2 domain-containing protein	Unknown protein	OuterMembrane		2066	2066
Gly-zipper_Omp domain-containing protein	Unknown protein	Extracellular		467	467
DUF2860 family protein	Unknown protein	OuterMembrane		998	998
terminal transmembrane domain-containing protein	Unknown protein	OuterMembrane		1637	1637
Extracellular lipase	Unknown protein	OuterMembrane		2414	2417
ahh1	Unknown protein	OuterMembrane		1865	1865
Bacillus haemolytic enterotoxin (HBL)	Unknown protein	Extracellular		1079	1079
group_449	Unknown protein	OuterMembrane		800	800
aerA/act	Unknown protein	Extracellular		1481	1481
ToxR-regulated lipoprotein	Unknown protein	OuterMembrane		2378	2378
UPF0265 protein ASA_0299	Function unknown	Cytoplasmic		317	317
UPF0307 protein ASA_0301	Translation, ribosomal structure and biogenesis	Cytoplasmic		524	524
Protein PmbA	General function prediction only	Cytoplasmic		1343	1343
murA	Cell wall/membrane/envelope biogenesis	Cytoplasmic		1256	1256
ibaG	Signal transduction mechanisms	Cytoplasmic		257	257
Organic solvent ABC transporter substrate-binding protein	Cell wall/membrane/envelope biogenesis	Periplasmic		632	632
mlaD	Cell wall/membrane/envelope biogenesis	Periplasmic		491	491
Intermembrane phospholipid transport system permease protein MlaE	Cell wall/membrane/envelope biogenesis	InnerMembrane		779	779
ABC transporter ATP-binding protein	Cell wall/membrane/envelope biogenesis	Cytoplasmic		800	800
Arabinose 5-phosphate isomerase	Carbohydrate transport and metabolism	Cytoplasmic		995	995
3-deoxy-D-manno-octulosonate 8-phosphate phosphatase KdsC	Cell wall/membrane/envelope biogenesis	Cytoplasmic		554	554
lptC	Cell wall/membrane/envelope biogenesis	Cytoplasmic		560	560
lptA	Cell wall/membrane/envelope biogenesis	Periplasmic		542	542
lptB	Cell wall/membrane/envelope biogenesis	Cytoplasmic		725	725
rpoN	Transcription	Cytoplasmic		1439	1439
raiA	Translation, ribosomal structure and biogenesis	Cytoplasmic		287	287
ptsN	Carbohydrate transport and metabolism	Cytoplasmic		446	446

Nucleotide-binding protein ASA_0318	Signal transduction mechanisms	Cytoplasmic		866	866
phosphotransferase component of N-regulated PTS system (Npr)	Signal transduction mechanisms	Cytoplasmic		272	272
Putative PTS system EIIA component	Transcription	Cytoplasmic		1904	1904
2-dehydro-3-deoxy-phosphogluconate aldolase	Unknown protein	Cytoplasmic		740	740
D-glucosaminate-6-phosphate ammonia-lyase	Translation, ribosomal structure and biogenesis	Cytoplasmic		1115	1115
Phosphotransferase system	Unknown protein	InnerMembrane		650	650
DUF4311 domain-containing protein	Unknown protein	InnerMembrane		776	776
group_1224	Unknown protein	Cytoplasmic		365	365
Periplasmic serine endoprotease DegP-like	Posttranslational modification, protein turnover, chaperones	Periplasmic		1361	1361
Putative cytochrome d ubiquinol oxidase subunit 3	Function unknown	Cytoplasmic		380	380
Cell division protein ZapE	Cell cycle control, cell division, chromosome partitioning	Cytoplasmic		1094	1094
rplM	Translation, ribosomal structure and biogenesis	Cytoplasmic		428	428
rpsI	Translation, ribosomal structure and biogenesis	Cytoplasmic		392	392
petA	Energy production and conversion	Periplasmic		590	590
Cytochrome b	Energy production and conversion	InnerMembrane		1217	1217
Cytochrome c1	Energy production and conversion	Periplasmic		734	734
sspA	Posttranslational modification, protein turnover, chaperones	Cytoplasmic		629	629
ClpXP protease specificity-enhancing factor	Posttranslational modification, protein turnover, chaperones	Cytoplasmic		425	425
BON domain-containing protein	Cell wall/membrane/envelope biogenesis	Periplasmic		578	578
gmhA	Carbohydrate transport and metabolism	Cytoplasmic		590	590
UPF0102 protein CW748_01405	Replication, recombination and repair	Cytoplasmic		293	371
group_1230	Cell wall/membrane/envelope biogenesis	Periplasmic		1787	1934
rsmI	Translation, ribosomal structure and biogenesis	Cytoplasmic		833	833
mraZ	Translation, ribosomal structure and biogenesis	Cytoplasmic		458	458
rsmH	Translation, ribosomal structure and biogenesis	Cytoplasmic		938	938
ftsL	Cell cycle control, cell division, chromosome partitioning	InnerMembrane		314	314
Peptidoglycan D	Cell cycle control, cell division, chromosome partitioning	InnerMembrane		1754	1754
murE	Cell wall/membrane/envelope biogenesis	Cytoplasmic		1484	1484
murF	Cell wall/membrane/envelope biogenesis	Cytoplasmic		1355	1355
mraY	Cell wall/membrane/envelope biogenesis	InnerMembrane		1082	1082
murD	Cell wall/membrane/envelope biogenesis	Cytoplasmic		1283	1283
ftsW	Cell cycle control, cell division, chromosome partitioning	InnerMembrane		1181	1181
murG	Cell wall/membrane/envelope biogenesis	Cytoplasmic		1079	1085
murC	Cell wall/membrane/envelope biogenesis	Cytoplasmic		1454	1460
Cell division protein FtsQ	Cell cycle control, cell division, chromosome partitioning	Cytoplasmic		752	752
ftsA	Cell cycle control, cell division, chromosome partitioning	Cytoplasmic		1259	1259

ftsZ	Cell cycle control, cell division, chromosome partitioning	Cytoplasmic		1151	1151
lpxC	Cell wall/membrane/envelope biogenesis	Cytoplasmic		848	848
Peptidase_M23 domain-containing protein	Cell wall/membrane/envelope biogenesis	Periplasmic		905	905
secA	Intracellular trafficking, secretion, and vesicular transport	Cytoplasmic		2717	2720
Mutator MutT protein	Nucleotide transport and metabolism	Cytoplasmic		413	413
Cell division protein ZapD	Cell cycle control, cell division, chromosome partitioning	Cytoplasmic		722	722
tapD	Cell motility	InnerMembrane		872	872
tapC	Cell motility	InnerMembrane		1241	1241
tapB	Cell motility	Cytoplasmic		1706	1706
pdhR	Transcription	Cytoplasmic		764	764
aceE	Energy production and conversion	Cytoplasmic		2660	2660
aceF	Energy production and conversion	Cytoplasmic		1889	1901
group_6110	Energy production and conversion	Cytoplasmic		1427	1427
group_1239	Unknown protein	Periplasmic		182	182
acnB	Energy production and conversion	Cytoplasmic		2597	2597
group_1483	Amino acid transport and metabolism	InnerMembrane		617	617
FAD-binding PCMH-type domain-containing protein	Energy production and conversion	Cytoplasmic		1013	1394
CobW C-terminal domain-containing protein	General function prediction only	Cytoplasmic		1127	1127
Major facilitator transporter	Carbohydrate transport and metabolism	InnerMembrane		1388	1388
group_5895	Translation, ribosomal structure and biogenesis	Cytoplasmic		482	482
folB	Coenzyme transport and metabolism	Cytoplasmic		371	371
plsY	Lipid transport and metabolism	InnerMembrane		662	710
D-alanyl-D-alanine carboxypeptidase	Cell wall/membrane/envelope biogenesis	Periplasmic		827	875
group_5938	Cell wall/membrane/envelope biogenesis	InnerMembrane		665	665
CorC_HlyC domain-containing protein	General function prediction only	InnerMembrane		1292	1292
valS	Translation, ribosomal structure and biogenesis	Cytoplasmic		2852	2852
DNA polymerase 3 chi subunit	Replication, recombination and repair	Periplasmic		455	455
pepA	Amino acid transport and metabolism	Cytoplasmic		1508	1508
lptF	Cell wall/membrane/envelope biogenesis	InnerMembrane		1112	1112
lptG	Cell wall/membrane/envelope biogenesis	InnerMembrane		1070	1070
group_1259	Unknown protein	Periplasmic		293	293
DUF2517 family protein	Unknown protein	Cytoplasmic		200	200
DUF2282 domain-containing protein	Function unknown	Periplasmic		263	263
ABC-type multidrug transport system	Unknown protein	Periplasmic		557	557
Inner membrane protein YeeA	Function unknown	InnerMembrane		1058	1082
mobA	Coenzyme transport and metabolism	Cytoplasmic		638	638

group_5915	Inorganic ion transport and metabolism	Cytoplasmic		695	695
Molybdenum transport system permease protein ModB	Inorganic ion transport and metabolism	InnerMembrane		701	701
PBP_domain domain-containing protein	Inorganic ion transport and metabolism	Periplasmic		842	842
Acetoacetate metabolism regulatory protein AtoC	Signal transduction mechanisms	Cytoplasmic		1355	1355
Histidine kinase	Signal transduction mechanisms	InnerMembrane		2093	2093
Excisionase family DNA binding domain-containing protein	Inorganic ion transport and metabolism	Cytoplasmic		878	878
group_1251	Energy production and conversion	Cytoplasmic		857	857
Transcriptional initiation protein Tat	Unknown protein	Cytoplasmic		203	203
4Fe-4S Mo/W bis-MGD-type domain-containing protein	Energy production and conversion	Periplasmic		2726	2900
Malate dehydrogenase	Energy production and conversion	Cytoplasmic		1715	1715
Uracil permease	Nucleotide transport and metabolism	InnerMembrane		1238	1238
group_1249	Unknown protein	Cytoplasmic		833	833
pyk	Carbohydrate transport and metabolism	Cytoplasmic		1424	1424
M20/M25/M40 family metallo-hydrolase	Posttranslational modification, protein turnover, chaperones	Periplasmic		1181	1181
Putative monovalent cation:proton antiporter (CPA2 family)	Inorganic ion transport and metabolism	InnerMembrane		1643	1643
ggt	Amino acid transport and metabolism	Periplasmic		1376	1778
rdgC	Replication, recombination and repair	Cytoplasmic		911	911
Acetyltransferase	Translation, ribosomal structure and biogenesis	Cytoplasmic		428	428
sbcD	Replication, recombination and repair	Cytoplasmic		1226	1226
group_195	Replication, recombination and repair	Cytoplasmic		3584	3755
Cytochrome B	Energy production and conversion	InnerMembrane		545	548
PTS glucose transporter subunit IIA	Carbohydrate transport and metabolism	Cytoplasmic		509	509
group_6428	Carbohydrate transport and metabolism	Cytoplasmic		1727	1727
group_5832	Signal transduction mechanisms	Cytoplasmic		257	257
cysK	Amino acid transport and metabolism	Cytoplasmic		968	968
group_194	General function prediction only	Cytoplasmic		794	842
VWA domain-containing protein	Function unknown	Cytoplasmic		1163	1163
3-oxoacyl-ACP synthase	Lipid transport and metabolism	Cytoplasmic		1121	1121
tsaA	Translation, ribosomal structure and biogenesis	Cytoplasmic		707	707
DUF488 domain-containing protein	Function unknown	Periplasmic		392	392
group_5889	Unknown protein	Periplasmic		425	425
cydC	Energy production and conversion	InnerMembrane		1745	1745
cydD	Energy production and conversion	InnerMembrane		1769	1769
4-hydroxy-4-methyl-2-oxoglutarate aldolase	Translation, ribosomal structure and biogenesis	Cytoplasmic		479	479
group_1449	Posttranslational modification, protein turnover, chaperones	Periplasmic		398	398
aceK	Signal transduction mechanisms	Cytoplasmic		1727	1727

group_1448	Translation, ribosomal structure and biogenesis	Cytoplasmic		692	692
thrA	Amino acid transport and metabolism	Cytoplasmic		2459	2492
thrB	Amino acid transport and metabolism	Cytoplasmic		980	980
thrC	Amino acid transport and metabolism	Cytoplasmic		1274	1274
DUF4440 domain-containing protein	General function prediction only	Cytoplasmic		416	416
group_1445	Unknown protein	Periplasmic		380	380
deoD	Nucleotide transport and metabolism	Cytoplasmic		701	701
MFS transporter TsgA	Carbohydrate transport and metabolism	InnerMembrane		1163	1163
Cyclic nucleotide-binding domain-containing protein	Signal transduction mechanisms	Cytoplasmic		677	677
group_1444	Signal transduction mechanisms	InnerMembrane		3269	3269
LigB domain-containing protein	Secondary metabolites biosynthesis, transport and catabolism	Periplasmic		767	767
group_6483	Posttranslational modification, protein turnover, chaperones	Cytoplasmic		470	470
Sodium dependent phosphate pump	Inorganic ion transport and metabolism	InnerMembrane		1196	1196
group_5943	Unknown protein	Periplasmic		380	380
Putrescine importer PuuP	Amino acid transport and metabolism	InnerMembrane		1343	1343
group_5693	Unknown protein	Periplasmic		149	149
APC family permease	Amino acid transport and metabolism	InnerMembrane		1343	1343
group_5790	Unknown protein	Cytoplasmic		125	125
gabT	Amino acid transport and metabolism	Cytoplasmic		1289	1289
gabD	Lipid transport and metabolism	Cytoplasmic		1463	1463
PuuR family transcription regulator PuuR	Transcription	Periplasmic		611	611
DAO domain-containing protein	Amino acid transport and metabolism	Cytoplasmic		1277	1277
SRPBCC domain-containing protein	Lipid transport and metabolism	Cytoplasmic		500	500
group_6101	Translation, ribosomal structure and biogenesis	Cytoplasmic		422	422
group_6420	Transcription	Cytoplasmic		410	410
group_1154	Amino acid transport and metabolism	Cytoplasmic		1106	1106
FAD-binding FR-type domain-containing protein	Inorganic ion transport and metabolism	Cytoplasmic		806	806
Usp domain-containing protein	Unknown protein	Cytoplasmic		440	440
Pyrophosphate phospho-hydrolase	Energy production and conversion	Cytoplasmic		893	893
recN	Replication, recombination and repair	Cytoplasmic		1337	1664
ppnK	Coenzyme transport and metabolism	Cytoplasmic		884	884
grpE	Posttranslational modification, protein turnover, chaperones	Cytoplasmic		575	575
dnaK	Posttranslational modification, protein turnover, chaperones	Cytoplasmic		1928	1928
Chaperone protein DnaJ	Posttranslational modification, protein turnover, chaperones	Cytoplasmic		1142	1142
Pts system	Carbohydrate transport and metabolism	InnerMembrane		1364	1388
Sucrose operon repressor	Transcription	Cytoplasmic		1025	1025

Protein MgtC	Inorganic ion transport and metabolism	InnerMembrane		728	728
group_1151	Unknown protein	Cytoplasmic		374	374
DUF2892 domain-containing protein	Unknown protein	Cytoplasmic		185	185
HTH arsR-type domain-containing protein	Transcription	Cytoplasmic		314	317
Sulf_transp domain-containing protein	General function prediction only	InnerMembrane		410	410
YeeE/YedE family protein	General function prediction only	InnerMembrane		446	446
Antibiotic efflux pump membrane transporter ArpB	Defense mechanisms	InnerMembrane		3197	3197
PAS:GGDEF domain protein	Signal transduction mechanisms	Cytoplasmic		2429	2432
lplA	Coenzyme transport and metabolism	Cytoplasmic		1016	1016
Decarboxylase	Amino acid transport and metabolism	Cytoplasmic		1694	1694
group_1144	Unknown protein	Cytoplasmic		788	788
Heavy metal efflux pump CzcA	Inorganic ion transport and metabolism	InnerMembrane		3128	3128
Efflux transporter	Cell wall/membrane/envelope biogenesis	Periplasmic		1517	1532
group_1142	Unknown protein	Periplasmic		437	437
Efflux pump membrane transporter	Defense mechanisms	InnerMembrane		3128	3128
Acriflavin resistance protein A	Cell wall/membrane/envelope biogenesis	Periplasmic		1199	1199
xth	Replication, recombination and repair	Cytoplasmic		806	806
Ion_trans_2 domain-containing protein	Function unknown	Cytoplasmic		962	962
TPR repeat-containing response regulator	Signal transduction mechanisms	Cytoplasmic		1718	1718
group_6361	Carbohydrate transport and metabolism	InnerMembrane		1205	1205
group_6635	Coenzyme transport and metabolism	Periplasmic		1040	1040
azu	Energy production and conversion	Periplasmic		440	440
HutD family protein	Function unknown	Cytoplasmic		557	560
IU_nuc_hydro domain-containing protein	Nucleotide transport and metabolism	Cytoplasmic		968	968
DUF3634 family protein	Unknown protein	Periplasmic		335	335
Cytochrome c family protein	Energy production and conversion	Periplasmic		1199	1199
Twin-arginine translocation pathway signal	Secondary metabolites biosynthesis, transport and catabolism	Periplasmic		1925	1925
cyoA	Energy production and conversion	InnerMembrane		1070	1070
cyoB	Energy production and conversion	InnerMembrane		1976	1976
cyoC	Energy production and conversion	InnerMembrane		611	611
cyoD	Energy production and conversion	InnerMembrane		329	332
cyoE	Coenzyme transport and metabolism	InnerMembrane		896	896
group_6021	Carbohydrate transport and metabolism	InnerMembrane		1361	1361
group_5941	Nucleotide transport and metabolism	InnerMembrane		1211	1211
Inhibitor of vertebrate lysozyme	Unknown protein	Periplasmic		443	443
DUF3297 family protein	Unknown protein	Periplasmic		242	242

trpA	Amino acid transport and metabolism	Cytoplasmic		806	806
trpB	Amino acid transport and metabolism	Cytoplasmic		1193	1193
Multifunctional fusion protein	Amino acid transport and metabolism	Cytoplasmic		1496	1496
trpD	Amino acid transport and metabolism	Cytoplasmic		1013	1028
Anthranilate synthase component II	Amino acid transport and metabolism	Cytoplasmic		599	599
trpE	Amino acid transport and metabolism	Cytoplasmic		1634	1634
POLIIIAc domain-containing protein	Nucleotide transport and metabolism	Cytoplasmic		881	881
YrdC-like domain-containing protein	Translation, ribosomal structure and biogenesis	Cytoplasmic		620	620
Contig_52	Replication, recombination and repair	Cytoplasmic		869	869
scpB	Transcription	Cytoplasmic		596	596
group_5860	Translation, ribosomal structure and biogenesis	Cytoplasmic		923	923
tesB	Lipid transport and metabolism	Cytoplasmic		863	863
Proton/sodium-glutamate symport protein	Amino acid transport and metabolism	InnerMembrane		1178	1178
group_466	Transcription	Cytoplasmic		686	686
adeB	Defense mechanisms	InnerMembrane		3149	3149
Outer membrane protein assembly factor BamE	Cell wall/membrane/envelope biogenesis	Periplasmic		338	338
Ribosome association toxin RatA	Translation, ribosomal structure and biogenesis	Cytoplasmic		434	434
smpB	Posttranslational modification, protein turnover, chaperones	Cytoplasmic		488	488
dsdA	Amino acid transport and metabolism	Cytoplasmic		1331	1364
icd	Energy production and conversion	Cytoplasmic		1253	1253
HTH-type transcriptional regulator YijO	Transcription	Cytoplasmic		875	875
manA	Carbohydrate transport and metabolism	Cytoplasmic		1199	1199
group_400	Unknown protein	Periplasmic		506	506
PTS fructose transporter subunit EIIC	Carbohydrate transport and metabolism	InnerMembrane		1886	1889
malX	Carbohydrate transport and metabolism	InnerMembrane		1571	1586
group_6439	Carbohydrate transport and metabolism	InnerMembrane		1907	1907
uhpT	Carbohydrate transport and metabolism	InnerMembrane		1406	1406
Phosphotransferase system enzyme II	Carbohydrate transport and metabolism	Cytoplasmic		305	305
Phosphocarrier protein HPr	Carbohydrate transport and metabolism	Cytoplasmic		767	767
mnmA	Translation, ribosomal structure and biogenesis	Cytoplasmic		1106	1106
hflD	Mobilome: prophages, transposons	InnerMembrane		611	614
purB	Nucleotide transport and metabolism	Cytoplasmic		1370	1370
JmjC domain-containing protein	Translation, ribosomal structure and biogenesis	Cytoplasmic		1130	1130
group_396	General function prediction only	Cytoplasmic		509	515
pdxH	Coenzyme transport and metabolism	Cytoplasmic		638	638
grxD	Posttranslational modification, protein turnover, chaperones	Cytoplasmic		341	344

Lipoprotein VacJ	Cell wall/membrane/envelope biogenesis	Periplasmic		812	812
Cytochrome c-type biogenesis protein	Energy production and conversion	InnerMembrane		479	479
DsbE family thiol:disulfide interchange protein	Posttranslational modification, protein turnover, chaperones	InnerMembrane		530	530
nrfE	Energy production and conversion	InnerMembrane		1955	1955
ccmE	Energy production and conversion	Periplasmic		491	491
ccmD	Intracellular trafficking, secretion, and vesicular transport	Cytoplasmic		206	206
ccmC	Posttranslational modification, protein turnover, chaperones	InnerMembrane		740	740
ccmB	Posttranslational modification, protein turnover, chaperones	InnerMembrane		668	668
ccmA	Posttranslational modification, protein turnover, chaperones	Cytoplasmic		635	635
group_391	Unknown protein	Cytoplasmic		392	392
cheW-2	Signal transduction mechanisms	Cytoplasmic		488	488
AHA_1389	Cell cycle control, cell division, chromosome partitioning	InnerMembrane		794	794
motA	Cell motility	InnerMembrane		737	737
cheB-2	Signal transduction mechanisms	Cytoplasmic		1109	1115
cheZ	Cell motility	Cytoplasmic		740	740
cheY	Signal transduction mechanisms	Cytoplasmic		371	371
fliA	Transcription	Cytoplasmic		719	719
fleN	Cell cycle control, cell division, chromosome partitioning	Cytoplasmic		884	884
flhF	Cell motility	Cytoplasmic		1412	1421
flhA	Cell motility	InnerMembrane		2102	2102
flhB	Cell motility	InnerMembrane		1130	1130
fliR	Cell motility	InnerMembrane		791	791
fliQ	Cell motility	InnerMembrane		269	269
fliP	Cell motility	InnerMembrane		779	779
fliO	Cell motility	InnerMembrane		383	383
fliN	Cell motility	Cytoplasmic		392	392
fliM	Cell motility	Cytoplasmic		1070	1070
fliL	Cell motility	Periplasmic		518	518
fliJ	Cell motility	Periplasmic		440	440
fliI	Cell motility	Cytoplasmic		1331	1331
fliH	Cell motility	Cytoplasmic		806	806
fliG	Cell motility	Cytoplasmic		1061	1061
fliE	Cell motility	Periplasmic		317	317
rimO	Translation, ribosomal structure and biogenesis	Cytoplasmic		1328	1328
Cytolysin-activating lysine-acyltransferase RtxC	Posttranslational modification, protein turnover, chaperones	Cytoplasmic		395	395
group_379	Unknown protein	Periplasmic		329	329

rtxB	Defense mechanisms	InnerMembrane		2063	2087
Membrane fusion protein (MFP) family protein	Defense mechanisms	InnerMembrane		1364	1364
rtxE	Defense mechanisms	InnerMembrane		2159	2168
group_6577	Signal transduction mechanisms	Cytoplasmic		689	689
group_375	Signal transduction mechanisms	InnerMembrane		1382	1382
glyA	Amino acid transport and metabolism	Cytoplasmic		1253	1253
nrdR	Transcription	Cytoplasmic		449	449
ribD	Coenzyme transport and metabolism	Cytoplasmic		1109	1109
ribB	Coenzyme transport and metabolism	Cytoplasmic		1109	1109
nusB	Transcription	Cytoplasmic		413	413
thiL	Coenzyme transport and metabolism	Cytoplasmic		959	977
group_6549	Posttranslational modification, protein turnover, chaperones	Cytoplasmic		1724	1724
group_6715	Amino acid transport and metabolism	Periplasmic		1556	1556
ABC transporter permease	Amino acid transport and metabolism	InnerMembrane		974	974
group_6522	Amino acid transport and metabolism	InnerMembrane		941	941
dxs	Coenzyme transport and metabolism	Cytoplasmic		1865	1865
(2E	Coenzyme transport and metabolism	Cytoplasmic		890	890
xseB	Replication, recombination and repair	Cytoplasmic		251	251
greA	Transcription	Cytoplasmic		476	476
23S rRNA methyltransferase J	Translation, ribosomal structure and biogenesis	Cytoplasmic		296	296
rlmE	Translation, ribosomal structure and biogenesis	Cytoplasmic		629	629
hflB	Posttranslational modification, protein turnover, chaperones	InnerMembrane		1949	1949
folP	Coenzyme transport and metabolism	Cytoplasmic		845	845
glmM	Carbohydrate transport and metabolism	Cytoplasmic		1334	1334
secG	Intracellular trafficking, secretion, and vesicular transport	Periplasmic		326	326
rimP	Translation, ribosomal structure and biogenesis	Cytoplasmic		458	458
nusA	Transcription	Cytoplasmic		1502	1502
infB	Translation, ribosomal structure and biogenesis	Cytoplasmic		2693	2702
rbfA	Translation, ribosomal structure and biogenesis	Cytoplasmic		434	434
truB	Translation, ribosomal structure and biogenesis	Cytoplasmic		950	959
rpsO	Translation, ribosomal structure and biogenesis	Cytoplasmic		269	269
pnp	Translation, ribosomal structure and biogenesis	Cytoplasmic		2138	2138
Protein of uncharacterized function (DUF1090)	Unknown protein	Cytoplasmic		392	395
fumC	Energy production and conversion	Cytoplasmic		1394	1394
Dipeptidase E	Amino acid transport and metabolism	Cytoplasmic		728	728
group_6220	Unknown protein	Periplasmic		1361	1364

group_756	Lipid transport and metabolism	Cytoplasmic		836	848
Pyoverdine chromophore biosynthetic protein PvcA	Secondary metabolites biosynthesis, transport and catabolism	Cytoplasmic		974	974
TauD domain-containing protein	Secondary metabolites biosynthesis, transport and catabolism	Cytoplasmic		875	875
group_759	Cell wall/membrane/envelope biogenesis	Periplasmic		926	926
Putative glyoxalase	General function prediction only	Cytoplasmic		374	374
Diacylglycerol O-acyltransferase	General function prediction only	Cytoplasmic		656	1424
Putative polyhydroxyalkanoic acid system protein	Unknown protein	Cytoplasmic		332	332
group_264	Unknown protein	Cytoplasmic		743	743
prpD	Carbohydrate transport and metabolism	Cytoplasmic		1445	1445
Citrate synthase	Energy production and conversion	Cytoplasmic		1127	1127
prpB	Carbohydrate transport and metabolism	Cytoplasmic		887	887
group_266	Cell wall/membrane/envelope biogenesis	InnerMembrane		632	632
Paraquat-inducible protein A	Cell wall/membrane/envelope biogenesis	InnerMembrane		608	608
Paraquat-inducible protein B	Cell wall/membrane/envelope biogenesis	InnerMembrane		2552	2552
rsmF	Translation, ribosomal structure and biogenesis	Cytoplasmic		1427	1427
Phage integrase family protein	Replication, recombination and repair	Cytoplasmic		989	1028
Deoxyribonuclease YcfH	Cell motility	Cytoplasmic		779	779
tmk	Nucleotide transport and metabolism	Cytoplasmic		641	641
Endolytic murein transglycosylase	Cell wall/membrane/envelope biogenesis	Cytoplasmic		1001	1001
fabF	Lipid transport and metabolism	Cytoplasmic		1241	1241
acpP	Lipid transport and metabolism	Cytoplasmic		236	236
flmH	Lipid transport and metabolism	Cytoplasmic		734	734
fabD	Lipid transport and metabolism	Cytoplasmic		935	935
fabH	Lipid transport and metabolism	Cytoplasmic		959	959
plsX	Lipid transport and metabolism	Cytoplasmic		809	1019
rpmF	Translation, ribosomal structure and biogenesis	Cytoplasmic		167	167
23S rRNA accumulation protein YceD	Translation, ribosomal structure and biogenesis	Cytoplasmic		431	521
group_274	Translation, ribosomal structure and biogenesis	Cytoplasmic		983	983
Phosphate transporter	Inorganic ion transport and metabolism	InnerMembrane		1469	1469
dcd	Nucleotide transport and metabolism	Cytoplasmic		581	581
udk	Nucleotide transport and metabolism	Cytoplasmic		644	644
Iron-sulfur cluster carrier protein	Cell cycle control, cell division, chromosome partitioning	Cytoplasmic		1082	1082
group_277	Translation, ribosomal structure and biogenesis	Cytoplasmic		2012	2012
group_5698	Posttranslational modification, protein turnover, chaperones	Cytoplasmic		635	635
acyP	Energy production and conversion	Cytoplasmic		272	272
group_278	Signal transduction mechanisms	InnerMembrane		1430	1430

rlmI	Translation, ribosomal structure and biogenesis	Cytoplasmic		1193	1280
Glutathione S-transferase	Posttranslational modification, protein turnover, chaperones	Cytoplasmic		644	644
Acyl_transf_3 domain-containing protein	Cell wall/membrane/envelope biogenesis	InnerMembrane		1184	1184
selD	Amino acid transport and metabolism	Cytoplasmic		1037	1037
Two-component system response regulator	Signal transduction mechanisms	Cytoplasmic		1001	1001
rplY	Translation, ribosomal structure and biogenesis	Cytoplasmic		287	287
hrpA	Translation, ribosomal structure and biogenesis	Cytoplasmic		3899	3914
CBS domain protein	Signal transduction mechanisms	Cytoplasmic		422	422
Nucleoid-associated protein G113_08675	Transcription	Cytoplasmic		329	329
apt	Nucleotide transport and metabolism	Cytoplasmic		545	545
tRNA-dihydrouridine(16) synthase	Translation, ribosomal structure and biogenesis	Cytoplasmic		944	944
rnd	Translation, ribosomal structure and biogenesis	Cytoplasmic		1112	1112
Long-chain-acyl--CoA ligase	Lipid transport and metabolism	Cytoplasmic		1766	1766
Outer membrane lipoprotein Slp family	Cell wall/membrane/envelope biogenesis	Periplasmic		515	515
Helicase ATP-binding domain-containing protein	Replication, recombination and repair	Cytoplasmic		1910	1922
group_290	Unknown protein	Cytoplasmic		242	242
hisG	Amino acid transport and metabolism	Cytoplasmic		896	896
hisD	Amino acid transport and metabolism	Cytoplasmic		1325	1325
Histidinol-phosphate aminotransferase	Amino acid transport and metabolism	Cytoplasmic		1070	1070
hisB	Amino acid transport and metabolism	Cytoplasmic		1130	1151
hisA	Amino acid transport and metabolism	Cytoplasmic		743	749
hisF	Amino acid transport and metabolism	Cytoplasmic		773	773
hisE	Amino acid transport and metabolism	Cytoplasmic		635	635
AMP-binding protein	Lipid transport and metabolism	Cytoplasmic		1622	1625
group_6133	General function prediction only	InnerMembrane		1526	1526
group_5737	Unknown protein	Cytoplasmic		188	188
group_6024	Function unknown	Periplasmic		545	545
LTD domain-containing protein	Unknown protein	Periplasmic		338	3218
DUF1456 domain-containing protein	Function unknown	Cytoplasmic		479	479
group_1279	Signal transduction mechanisms	InnerMembrane		2711	2711
rlmD	Translation, ribosomal structure and biogenesis	Cytoplasmic		1322	1322
relA	Signal transduction mechanisms	Cytoplasmic		2210	2213
mazG	General function prediction only	Cytoplasmic		797	797
pyrG	Nucleotide transport and metabolism	Cytoplasmic		1637	1637
eno	Carbohydrate transport and metabolism	Cytoplasmic		1301	1301
Cell division protein FtsB	Cell cycle control, cell division, chromosome partitioning	Cytoplasmic		317	317

purL	Nucleotide transport and metabolism	Cytoplasmic		3905	3905
mltF	Cell wall/membrane/envelope biogenesis	Cytoplasmic		1457	1457
der	Translation, ribosomal structure and biogenesis	Cytoplasmic		1499	1499
TPR_21 domain-containing protein	Signal transduction mechanisms	Cytoplasmic		650	650
hisS	Translation, ribosomal structure and biogenesis	Cytoplasmic		1277	1277
ispG	Lipid transport and metabolism	Cytoplasmic		1067	1067
tapF	Cell motility	Periplasmic		776	776
rlmN	Translation, ribosomal structure and biogenesis	Cytoplasmic		1103	1103
ndk	Nucleotide transport and metabolism	Cytoplasmic		428	428
Aminopeptidase PepB	Amino acid transport and metabolism	Cytoplasmic		1283	1283
CYTOSOL_AP domain-containing protein	Amino acid transport and metabolism	Cytoplasmic		1043	1304
fdx	Energy production and conversion	Cytoplasmic		338	338
hscA	Posttranslational modification, protein turnover, chaperones	Cytoplasmic		1847	1847
sufA	Posttranslational modification, protein turnover, chaperones	Cytoplasmic		323	323
iscU	Posttranslational modification, protein turnover, chaperones	Periplasmic		383	383
iscS	Amino acid transport and metabolism	Cytoplasmic		1214	1214
iscR	Transcription	Periplasmic		473	473
Ubiquinone biosynthesis protein UbiU	Translation, ribosomal structure and biogenesis	Cytoplasmic		995	995
Ubiquinone biosynthesis protein UbiV	Translation, ribosomal structure and biogenesis	Cytoplasmic		872	872
Lipoprotein NlpI	Cell wall/membrane/envelope biogenesis	Cytoplasmic		947	947
Rhodanese domain-containing protein	Inorganic ion transport and metabolism	Cytoplasmic		368	368
secF	Intracellular trafficking, secretion, and vesicular transport	InnerMembrane		947	947
secD	Intracellular trafficking, secretion, and vesicular transport	InnerMembrane		1853	1853
yajC	Intracellular trafficking, secretion, and vesicular transport	InnerMembrane		335	335
tgt	Translation, ribosomal structure and biogenesis	Cytoplasmic		1136	1136
queA	Translation, ribosomal structure and biogenesis	Cytoplasmic		1067	1067
ahpC	Defense mechanisms	Cytoplasmic		566	566
ahpF	Defense mechanisms	Cytoplasmic		1580	1586
gcvP	Amino acid transport and metabolism	Cytoplasmic		1994	2876
Protein VisC	Coenzyme transport and metabolism	Cytoplasmic		1235	1235
ubiH	Coenzyme transport and metabolism	Cytoplasmic		1217	1217
UPF0149 family protein	Function unknown	Cytoplasmic		569	569
Cell division protein ZapA	Cell cycle control, cell division, chromosome partitioning	Cytoplasmic		332	332
group_493	Carbohydrate transport and metabolism	InnerMembrane		1199	1199
Disulfide-bond oxidoreductase YfcG	Posttranslational modification, protein turnover, chaperones	Cytoplasmic		662	662
Inositol-1-monophosphatase	Carbohydrate transport and metabolism	Cytoplasmic		803	803

group_492	Signal transduction mechanisms	InnerMembrane		1631	1631
GufA protein	Inorganic ion transport and metabolism	InnerMembrane		932	932
mtnN	Nucleotide transport and metabolism	Cytoplasmic		692	692
yadS	Function unknown	InnerMembrane		632	632
group_6129	Unknown protein	Periplasmic		392	392
thiI	Coenzyme transport and metabolism	Cytoplasmic		1448	1448
group_6601	Unknown protein	Cytoplasmic		959	959
pbpC	Cell wall/membrane/envelope biogenesis	InnerMembrane		2390	2399
Alpha-2-macroglobulin	General function prediction only	Periplasmic		4889	4889
group_488	Lipid transport and metabolism	Cytoplasmic		1688	1688
Diguanylate cyclase / EAL-type diguanylate phosphodiesterase	Signal transduction mechanisms	Cytoplasmic		2939	2939
pflA	Posttranslational modification, protein turnover, chaperones	Cytoplasmic		818	818
energy-coupling factor ABC transporter permease	Function unknown	InnerMembrane		641	641
group_6040	Amino acid transport and metabolism	InnerMembrane		692	692
Histidine/lysine/arginine/ornithine ABC transporter permease	Amino acid transport and metabolism	InnerMembrane		740	740
Arginine and ornithine binding protein	Amino acid transport and metabolism	Periplasmic		773	773
group_6618	Amino acid transport and metabolism	Cytoplasmic		770	770
Putative transporter	Amino acid transport and metabolism	InnerMembrane		902	902
iadA	Nucleotide transport and metabolism	Cytoplasmic		1130	1130
Putative beta-barrel assembly-enhancing protease	Cell wall/membrane/envelope biogenesis	Cytoplasmic		1433	1433
ppk2	Energy production and conversion	Cytoplasmic		815	815
Maltose/maltodextrin import ATP-binding protein MalK	Carbohydrate transport and metabolism	Cytoplasmic		1136	1136
malG	Carbohydrate transport and metabolism	InnerMembrane		890	890
malF	Carbohydrate transport and metabolism	InnerMembrane		1652	1652
malE	Carbohydrate transport and metabolism	Periplasmic		1181	1181
Nucleoid-associated protein NdpA	Replication, recombination and repair	Cytoplasmic		1049	1061
UPF0352 protein AHA_1665	Function unknown	Cytoplasmic		227	227
Inner membrane protein YejM	Cell wall/membrane/envelope biogenesis	InnerMembrane		1850	1850
Protease II	Amino acid transport and metabolism	Periplasmic		2066	2078
DUF1566 domain-containing protein	Unknown protein	Periplasmic		1343	1373
group_476	Unknown protein	Periplasmic		557	557
Ecotin	Posttranslational modification, protein turnover, chaperones	Periplasmic		482	482
UPF0438 protein YifE	Function unknown	Periplasmic		458	482
DNA-binding transcriptional regulator	Transcription	Cytoplasmic		875	875
tyrA	Amino acid transport and metabolism	Cytoplasmic		1139	1139
group_6590	Amino acid transport and metabolism	Cytoplasmic		1073	1073

GP-PDE domain-containing protein	Lipid transport and metabolism	Periplasmic		1079	1079
Ribonuclease	Translation, ribosomal structure and biogenesis	Periplasmic		647	647
Carbonate dehydratase	General function prediction only	Cytoplasmic		560	560
group_1561	Unknown protein	Periplasmic		293	293
Cytochrome-c peroxidase	Posttranslational modification, protein turnover, chaperones	Periplasmic		3008	3149
DNA-directed RNA polymerase subunit delta	Coenzyme transport and metabolism	Periplasmic		908	908
group_6480	Unknown protein	Cytoplasmic		455	455
group_6513	Carbohydrate transport and metabolism	InnerMembrane		1229	1232
group_6274	Transcription	Cytoplasmic		869	869
group_1559	Replication, recombination and repair	Cytoplasmic		1247	1247
Amidase	Translation, ribosomal structure and biogenesis	Periplasmic		1703	1703
aguA	Amino acid transport and metabolism	Cytoplasmic		1097	1097
group_1556	Transcription	Cytoplasmic		953	953
group_6684	Transcription	Cytoplasmic		566	566
group_6387	Energy production and conversion	Cytoplasmic		1037	1037
group_649	Transcription	Cytoplasmic		923	938
prfC	Translation, ribosomal structure and biogenesis	Cytoplasmic		1586	1586
VWFA domain-containing protein	Function unknown	Cytoplasmic		1454	1454
AAA domain-containing protein	General function prediction only	Cytoplasmic		1607	1607
rimI	Translation, ribosomal structure and biogenesis	Cytoplasmic		449	449
group_651	Unknown protein	Periplasmic		545	545
group_652	Lipid transport and metabolism	Cytoplasmic		1790	1790
gshA	Coenzyme transport and metabolism	Cytoplasmic		1598	1598
Peptidase family M16	General function prediction only	Periplasmic		2813	2813
group_655	Function unknown	InnerMembrane		437	437
group_5668	Unknown protein	InnerMembrane		167	167
group_656	Energy production and conversion	Cytoplasmic		740	740
rplI	Translation, ribosomal structure and biogenesis	Cytoplasmic		446	446
rpsR	Translation, ribosomal structure and biogenesis	Cytoplasmic		230	230
rpsF	Translation, ribosomal structure and biogenesis	Cytoplasmic		386	386
mntH	Inorganic ion transport and metabolism	InnerMembrane		1223	1223
EamA domain-containing protein	Carbohydrate transport and metabolism	InnerMembrane		836	836
group_659	Carbohydrate transport and metabolism	InnerMembrane		860	860
group_6263	General function prediction only	Cytoplasmic		449	449
Crp/Fnr family transcriptional regulator	Signal transduction mechanisms	Cytoplasmic		566	566
rnr	Transcription	Cytoplasmic		2393	2393

Diguanylate cyclase VdcA	Signal transduction mechanisms	Cytoplasmic		1022	1031
luxS	Signal transduction mechanisms	Cytoplasmic		509	509
Transcriptional regulatory protein	General function prediction only	Periplasmic		602	602
Nitrogen regulatory protein PII	Signal transduction mechanisms	Cytoplasmic		338	338
nadE	Coenzyme transport and metabolism	Cytoplasmic		1622	1622
tppB	Cell motility	Periplasmic		413	413
ispH	Lipid transport and metabolism	Cytoplasmic		935	935
group_665	Posttranslational modification, protein turnover, chaperones	Cytoplasmic		452	452
lspA	Cell wall/membrane/envelope biogenesis	InnerMembrane		503	503
ileS	Translation, ribosomal structure and biogenesis	Cytoplasmic		2861	2861
ribF	Coenzyme transport and metabolism	Cytoplasmic		974	974
mviN	Cell wall/membrane/envelope biogenesis	InnerMembrane		1565	1565
rpsT	Translation, ribosomal structure and biogenesis	Periplasmic		263	263
group_6117	Transcription	Cytoplasmic		302	302
nhaR	Transcription	Cytoplasmic		932	932
nhaA	Energy production and conversion	InnerMembrane		1190	1190
thyA	Nucleotide transport and metabolism	Cytoplasmic		794	794
lgt	Cell wall/membrane/envelope biogenesis	InnerMembrane		740	740
ptsP	Signal transduction mechanisms	Cytoplasmic		2279	2279
group_6059	Defense mechanisms	Cytoplasmic		380	380
mutH	Replication, recombination and repair	Cytoplasmic		674	674
group_672	Carbohydrate transport and metabolism	Cytoplasmic		329	329
rplS	Translation, ribosomal structure and biogenesis	Cytoplasmic		347	347
trmD	Translation, ribosomal structure and biogenesis	Cytoplasmic		749	749
rimM	Translation, ribosomal structure and biogenesis	Cytoplasmic		521	521
rpsP	Translation, ribosomal structure and biogenesis	Cytoplasmic		248	248
ffh	Intracellular trafficking, secretion, and vesicular transport	Cytoplasmic		1376	1376
Chromosome partitioning protein ParB	General function prediction only	InnerMembrane		791	791
DUF21 domain-containing protein	Inorganic ion transport and metabolism	InnerMembrane		1280	1280
mdh	Energy production and conversion	Cytoplasmic		935	935
artM	Amino acid transport and metabolism	InnerMembrane		647	662
ABC-type arginine transporter	Amino acid transport and metabolism	InnerMembrane		620	650
Lysine-arginine-ornithine-binding periplasmic protein	Amino acid transport and metabolism	Periplasmic		737	737
ABC transporter domain-containing protein	Amino acid transport and metabolism	InnerMembrane		740	740
argR	Transcription	Cytoplasmic		470	470
TyrA protein	Inorganic ion transport and metabolism	Periplasmic		905	905

Iron(III) ABC transporter ATP-binding protein	Amino acid transport and metabolism	Cytoplasmic		1034	1034
Iron(III) ABC transporter	Inorganic ion transport and metabolism	InnerMembrane		1625	1625
ABC-type iron(III) transporter	Inorganic ion transport and metabolism	Periplasmic		1007	1007
Nitrogen regulatory protein P-II 1	Signal transduction mechanisms	Cytoplasmic		338	338
Aminoacyl-tRNA hydrolase	Translation, ribosomal structure and biogenesis	Periplasmic		416	416
Nucleoside permease	Nucleotide transport and metabolism	InnerMembrane		1196	1196
group_6373	Transcription	Cytoplasmic		935	935
Glycerate kinase	Carbohydrate transport and metabolism	Cytoplasmic		1133	1133
H+/gluconate symporter	Carbohydrate transport and metabolism	InnerMembrane		1262	1262
Carbohydrate diacid regulator	Transcription	Cytoplasmic		1115	1115
Uncharacterised protein family (UPF0231)	Function unknown	Cytoplasmic		374	374
group_686	General function prediction only	Periplasmic		1316	1316
pulA	Carbohydrate transport and metabolism	Periplasmic		4085	4088
group_688	Inorganic ion transport and metabolism	Cytoplasmic		950	950
Metal-sensing transcriptional repressor	Transcription	Cytoplasmic		275	275
Aminotran_1_2 domain-containing protein	Amino acid transport and metabolism	Cytoplasmic		1196	1196
group_690	Transcription	InnerMembrane		923	923
DUF1820 family protein	Function unknown	Cytoplasmic		323	323
yjjl	Unknown protein	Cytoplasmic		1526	1526
Branched-subunit amino acid transport protein	Amino acid transport and metabolism	InnerMembrane		341	341
Azaleucine resistance protein AzlC	Amino acid transport and metabolism	InnerMembrane		719	719
YkuD domain-containing protein	Cell wall/membrane/envelope biogenesis	Periplasmic		476	476
nrfD	Inorganic ion transport and metabolism	InnerMembrane		599	953
group_1377	Energy production and conversion	InnerMembrane		1961	1961
nrfF	Energy production and conversion	InnerMembrane		755	1190
tnaA	Amino acid transport and metabolism	Cytoplasmic		1385	1385
dadA	Amino acid transport and metabolism	Cytoplasmic		1253	1253
HPP family	Signal transduction mechanisms	InnerMembrane		1127	1127
metE	Amino acid transport and metabolism	Cytoplasmic		2264	2264
group_6710	Transcription	Cytoplasmic		920	920
group_1373	Transcription	Periplasmic		899	899
phbB	Lipid transport and metabolism	Cytoplasmic		740	740
group_6108	Transcription	Cytoplasmic		905	905
NAD(P)-bd_dom domain-containing protein	General function prediction only	Cytoplasmic		638	638
megL	Amino acid transport and metabolism	Cytoplasmic		611	1163
asd	Amino acid transport and metabolism	Cytoplasmic		1115	1115

group_6685	Defense mechanisms	InnerMembrane		314	314
YcaO domain-containing protein	Translation, ribosomal structure and biogenesis	Cytoplasmic		353	1760
focA	Inorganic ion transport and metabolism	InnerMembrane		848	848
pflB	Energy production and conversion	Cytoplasmic		2282	2282
phnX	Carbohydrate transport and metabolism	Cytoplasmic		839	839
Acetylornithine aminotransferase	Amino acid transport and metabolism	Cytoplasmic		1424	1439
phnW	Amino acid transport and metabolism	Cytoplasmic		1142	1142
phnR	Transcription	Cytoplasmic		707	707
ABC transporter	Inorganic ion transport and metabolism	Periplasmic		1019	1019
Fe(3+) ions import ATP-binding protein FbpC	Amino acid transport and metabolism	Cytoplasmic		1094	1094
Putative 2-aminoethylphosphonate ABC transporter permease subunit	Inorganic ion transport and metabolism	InnerMembrane		1736	1736
UPF0260 protein AHA_1932	General function prediction only	Periplasmic		455	467
sucD	Energy production and conversion	Cytoplasmic		872	872
sucC	Energy production and conversion	Cytoplasmic		1166	1166
sucB	Energy production and conversion	Cytoplasmic		1187	1187
sucA	Energy production and conversion	Cytoplasmic		2810	2810
sdhB	Energy production and conversion	Cytoplasmic		716	716
sdhA	Energy production and conversion	Cytoplasmic		1709	1766
sdhD	Energy production and conversion	InnerMembrane		344	344
sdhC	Energy production and conversion	InnerMembrane		389	389
gltA	Energy production and conversion	Cytoplasmic		1286	1286
dcuC	Energy production and conversion	InnerMembrane		1352	1352
group_866	Unknown protein	InnerMembrane		1643	1643
Putative metal dependent hydrolase	General function prediction only	Cytoplasmic		503	503
DUF2927 domain-containing protein	Unknown protein	InnerMembrane		788	821
DinI family protein	Unknown protein	Cytoplasmic		248	248
rpmE2	Translation, ribosomal structure and biogenesis	Periplasmic		269	269
rpmJ1	Translation, ribosomal structure and biogenesis	Cytoplasmic		125	125
Quercetin 2	General function prediction only	Cytoplasmic		866	866
DNA deoxyribophosphodiesterase	Replication, recombination and repair	Cytoplasmic		1427	1427
Alpha-galactosidase	Carbohydrate transport and metabolism	Cytoplasmic		1709	1709
cdd	Nucleotide transport and metabolism	Periplasmic		881	881
YfcL family protein	Unknown protein	Cytoplasmic		278	278
UPF0053 protein HI_0056	Inorganic ion transport and metabolism	InnerMembrane		734	734
OmpA-like domain-containing protein	Cell wall/membrane/envelope biogenesis	Cytoplasmic		488	488
Transcriptional regulatory protein TyrR	Transcription	Cytoplasmic		1547	1547

Putative pterin-4-alpha-carbinolamine dehydratase	Coenzyme transport and metabolism	Cytoplasmic		338	338
phhA	Amino acid transport and metabolism	Cytoplasmic		794	794
TIGR01620 family protein	Function unknown	Cytoplasmic		1031	1031
Uncharacterized protein YcjX	Signal transduction mechanisms	Cytoplasmic		1406	1406
pspC	Transcription	Cytoplasmic		404	404
pspB	Unknown protein	Cytoplasmic		236	236
pspA	Transcription	Cytoplasmic		680	680
group_881	Amino acid transport and metabolism	Periplasmic		1595	1601
group_6210	Defense mechanisms	InnerMembrane		959	959
group_6323	Defense mechanisms	InnerMembrane		893	893
Peptide ABC transporter ATP-binding protein SapD	Defense mechanisms	Cytoplasmic		998	998
group_6168	Defense mechanisms	Cytoplasmic		785	785
tRNA-(Ms(2)io(6)A)-hydroxylase	Translation, ribosomal structure and biogenesis	Cytoplasmic		758	758
purF	Nucleotide transport and metabolism	Cytoplasmic		1520	1520
Bacteriocin production protein	Nucleotide transport and metabolism	InnerMembrane		488	488
serS	Translation, ribosomal structure and biogenesis	Cytoplasmic		1295	1295
Replication-associated recombination protein A	Replication, recombination and repair	Cytoplasmic		1337	1343
DNA translocase FtsK	Cell cycle control, cell division, chromosome partitioning	InnerMembrane		2522	2528
Leucine responsive regulatory protein	Transcription	Cytoplasmic		434	491
trxB	Posttranslational modification, protein turnover, chaperones	Cytoplasmic		950	950
Response regulator protein	Signal transduction mechanisms	Cytoplasmic		1247	1247
Rick_17kDa_Anti domain-containing protein	Cell wall/membrane/envelope biogenesis	Periplasmic		470	470
bpt	Posttranslational modification, protein turnover, chaperones	Cytoplasmic		716	716
infA	Translation, ribosomal structure and biogenesis	Cytoplasmic		218	218
clpA	Posttranslational modification, protein turnover, chaperones	Cytoplasmic		2252	2252
clpS	Posttranslational modification, protein turnover, chaperones	Cytoplasmic		317	317
cspD	Transcription	Cytoplasmic		218	218
folD	Coenzyme transport and metabolism	Cytoplasmic		863	863
rplQ	Translation, ribosomal structure and biogenesis	Cytoplasmic		383	383
rpoA	Transcription	Cytoplasmic		989	989
rpsD	Translation, ribosomal structure and biogenesis	Cytoplasmic		620	620
rpsK	Translation, ribosomal structure and biogenesis	Cytoplasmic		389	389
rpsM	Translation, ribosomal structure and biogenesis	Cytoplasmic		356	356
secY	Intracellular trafficking, secretion, and vesicular transport	InnerMembrane		1328	1328
rplO	Translation, ribosomal structure and biogenesis	Cytoplasmic		437	437
rpmD	Translation, ribosomal structure and biogenesis	Cytoplasmic		179	179

rpsE	Translation, ribosomal structure and biogenesis	Cytoplasmic		500	500
rplF	Translation, ribosomal structure and biogenesis	Cytoplasmic		533	533
rpsH	Translation, ribosomal structure and biogenesis	Cytoplasmic		392	392
rpsN	Translation, ribosomal structure and biogenesis	Cytoplasmic		305	305
rplE	Translation, ribosomal structure and biogenesis	Cytoplasmic		539	539
rplX	Translation, ribosomal structure and biogenesis	Cytoplasmic		317	317
rplN	Translation, ribosomal structure and biogenesis	Cytoplasmic		368	368
rpsQ	Translation, ribosomal structure and biogenesis	Cytoplasmic		248	248
rpmC	Translation, ribosomal structure and biogenesis	Cytoplasmic		191	191
rplP	Translation, ribosomal structure and biogenesis	Cytoplasmic		413	413
rpsC	Translation, ribosomal structure and biogenesis	Cytoplasmic		713	713
rplV	Translation, ribosomal structure and biogenesis	Cytoplasmic		347	347
rpsS	Translation, ribosomal structure and biogenesis	Cytoplasmic		278	278
rplB	Translation, ribosomal structure and biogenesis	Periplasmic		821	821
rplW	Translation, ribosomal structure and biogenesis	Cytoplasmic		302	302
rplD	Translation, ribosomal structure and biogenesis	Cytoplasmic		605	605
rplC	Translation, ribosomal structure and biogenesis	Cytoplasmic		632	632
rpsJ	Translation, ribosomal structure and biogenesis	Cytoplasmic		311	311
group_897	Carbohydrate transport and metabolism	InnerMembrane		914	914
group_898	Signal transduction mechanisms	Cytoplasmic		1310	1310
ompR	Signal transduction mechanisms	Cytoplasmic		719	719
greB	Transcription	Cytoplasmic		473	473
S1 motif domain-containing protein	Transcription	Cytoplasmic		2306	2309
group_900	Unknown protein	Cytoplasmic		278	278
Flagellar hook protein FlgE	Cell motility	Cytoplasmic		233	233
bioH	Coenzyme transport and metabolism	Cytoplasmic		764	764
nfuA	Posttranslational modification, protein turnover, chaperones	Cytoplasmic		578	578
gpsA	Energy production and conversion	Cytoplasmic		1004	1004
secB	Intracellular trafficking, secretion, and vesicular transport	Cytoplasmic		470	470
group_5763	Inorganic ion transport and metabolism	Periplasmic		431	431
gpmI	Carbohydrate transport and metabolism	Cytoplasmic		1529	1529
group_6487	Transcription	Cytoplasmic		1007	1007
eda	Carbohydrate transport and metabolism	Cytoplasmic		641	641
edd	Amino acid transport and metabolism	Cytoplasmic		1796	1796
idnK	Carbohydrate transport and metabolism	Cytoplasmic		509	509
Gluconate transporter	Carbohydrate transport and metabolism	InnerMembrane		1340	1340

pssA	Lipid transport and metabolism	Cytoplasmic		1349	1349
recG	Replication, recombination and repair	Cytoplasmic		2069	2069
group_906	General function prediction only	Cytoplasmic		908	908
Methyltransf_25 domain-containing protein	Amino acid transport and metabolism	Cytoplasmic		677	677
dtd	Translation, ribosomal structure and biogenesis	Cytoplasmic		437	437
UPF0761 membrane protein ASA_4118	Function unknown	InnerMembrane		872	872
typA	Signal transduction mechanisms	Cytoplasmic		1811	1811
glnA	Amino acid transport and metabolism	Cytoplasmic		1409	1409
glnL	Signal transduction mechanisms	Cytoplasmic		1055	1055
ntrC	Signal transduction mechanisms	Cytoplasmic		1418	1418
add	Nucleotide transport and metabolism	Cytoplasmic		1001	1001
hemN	Coenzyme transport and metabolism	Cytoplasmic		1373	1373
DUF2489 domain-containing protein	Unknown protein	Cytoplasmic		440	440
Cytochrome C	Energy production and conversion	Periplasmic		236	617
DUF1488 domain-containing protein	Unknown protein	Cytoplasmic		260	260
purE	Nucleotide transport and metabolism	Cytoplasmic		503	503
Topoisomerase DNA-binding C4 zinc finger domain-containing protein	Replication, recombination and repair	Periplasmic		557	557
smg	Function unknown	Cytoplasmic		473	473
LysM domain-containing protein	Inorganic ion transport and metabolism	Periplasmic		1088	1088
def	Translation, ribosomal structure and biogenesis	Cytoplasmic		512	512
fmt	Translation, ribosomal structure and biogenesis	Cytoplasmic		944	944
16S rRNA m5C967 methyltransferase	Translation, ribosomal structure and biogenesis	Cytoplasmic		1292	1292
trkA	Inorganic ion transport and metabolism	Cytoplasmic		1376	1376
group_918	Unknown protein	Cytoplasmic		215	215
Ferredoxin-type protein NapF	General function prediction only	Periplasmic		512	512
Rrf2 family protein	Transcription	Cytoplasmic		452	452
Carbohydrate kinase	Carbohydrate transport and metabolism	Cytoplasmic		839	839
ald	Amino acid transport and metabolism	Cytoplasmic		1115	1115
Glutamine amidotransferase type-1 domain-containing protein	Nucleotide transport and metabolism	Cytoplasmic		755	755
group_6484	Lipid transport and metabolism	Cytoplasmic		1496	1496
Beta alanine--pyruvate aminotransferase	Coenzyme transport and metabolism	Cytoplasmic		1331	1331
HTH cro/C1-type domain-containing protein	Transcription	Cytoplasmic		542	542
Aldehyde dehydrogenase	Lipid transport and metabolism	Cytoplasmic		1496	1496
rarD	General function prediction only	InnerMembrane		902	902
group_922	Transcription	Cytoplasmic		818	818
group_6762	Amino acid transport and metabolism	InnerMembrane		647	647

mgtA	Inorganic ion transport and metabolism	InnerMembrane		2762	2762
group_6668	General function prediction only	InnerMembrane		1109	1109
Lysine exporter protein	Amino acid transport and metabolism	InnerMembrane		620	620
group_924	Unknown protein	Periplasmic		674	674
Cupin_2 domain-containing protein	Function unknown	Cytoplasmic		512	512
Y1_Tnp domain-containing protein	Mobilome: prophages, transposons	Cytoplasmic		530	530
uvrD	Replication, recombination and repair	Cytoplasmic		2171	2171
Bmp domain-containing protein	Signal transduction mechanisms	Periplasmic		1103	1103
ABC transporter nucleotide binding/ATPase (Sugar/ribonucleotide)	Nucleotide transport and metabolism	Cytoplasmic		1580	1580
ABC-type sugar transporter	Nucleotide transport and metabolism	InnerMembrane		1115	1115
group_5869	Nucleotide transport and metabolism	InnerMembrane		965	965
group_6090	Transcription	Cytoplasmic		905	905
S-(hydroxymethyl)glutathione dehydrogenase	Energy production and conversion	Cytoplasmic		1127	1127
fghA	Defense mechanisms	Cytoplasmic		836	836
DUF2807 domain-containing protein	General function prediction only	Cytoplasmic		722	722
ovoA	Posttranslational modification, protein turnover, chaperones	Cytoplasmic		2150	2150
Putative sodium/bile acid symporter family (MazG-like)	General function prediction only	InnerMembrane		950	971
Elongation factor G	Amino acid transport and metabolism	InnerMembrane		1436	1445
metG	Translation, ribosomal structure and biogenesis	Cytoplasmic		338	338
DmsE family decaheme c-type cytochrome	Inorganic ion transport and metabolism	Periplasmic		971	971
DeCa-heme c-type cytochrome	Unknown protein	Periplasmic		2114	2114
Cytochrome c553	Energy production and conversion	Periplasmic		611	611
nrfC	Energy production and conversion	Periplasmic		668	668
group_1330	Unknown protein	InnerMembrane		1007	1007
DUF2157 domain-containing protein	Function unknown	InnerMembrane		938	938
GDT1 family protein	General function prediction only	InnerMembrane		557	557
yciH	Translation, ribosomal structure and biogenesis	Cytoplasmic		329	329
FeoA domain-containing protein	Inorganic ion transport and metabolism	Cytoplasmic		227	227
feoB	Inorganic ion transport and metabolism	InnerMembrane		2273	2273
FeoC domain-containing protein	Unknown protein	Cytoplasmic		242	242
Tryptophan synthase	Unknown protein	Cytoplasmic		479	479
Glucose-1-phosphate adenylyltransferase	Carbohydrate transport and metabolism	Cytoplasmic		1271	1271
tapW	Cell motility	Cytoplasmic		1139	1139
DMT family protein	Function unknown	InnerMembrane		356	356
Vitamin B12 ABC transporter permease BtuC	Coenzyme transport and metabolism	InnerMembrane		767	1007
group_1333	Inorganic ion transport and metabolism	InnerMembrane		785	785

Phospho-2-dehydro-3-deoxyheptonate aldolase	Amino acid transport and metabolism	Cytoplasmic		1061	1064
Diacylglycerol kinase	Lipid transport and metabolism	InnerMembrane		380	380
2-oxoglutarate reductase	Coenzyme transport and metabolism	Cytoplasmic		1232	1232
rpiA	Carbohydrate transport and metabolism	Cytoplasmic		653	653
5-formyltetrahydrofolate cyclo-ligase	Coenzyme transport and metabolism	Cytoplasmic		599	599
dapB	Amino acid transport and metabolism	Cytoplasmic		812	812
carA	Amino acid transport and metabolism	Cytoplasmic		1124	1136
carB	Amino acid transport and metabolism	Cytoplasmic		3236	3236
leuA	Amino acid transport and metabolism	Cytoplasmic		1658	1658
DedA family protein	Cell wall/membrane/envelope biogenesis	InnerMembrane		650	650
DEAD box family ATP-dependent RNA helicase	Replication, recombination and repair	Cytoplasmic		1295	1295
eprA1	Extracellular structures	Periplasmic		1040	1040
FAD-dependent oxidoreductase	Amino acid transport and metabolism	Cytoplasmic		1415	1415
gltB	Amino acid transport and metabolism	Cytoplasmic		4457	4457
Putative Fe-S oxidoreductase	General function prediction only	Cytoplasmic		935	935
Toxin secretion ATP-binding protein	Defense mechanisms	InnerMembrane		2144	2144
group_1345	Defense mechanisms	Cytoplasmic		1418	1418
Diguanylate phosphodiesterase	Signal transduction mechanisms	Cytoplasmic		452	1925
Putative PmbA-related protein	General function prediction only	Cytoplasmic		1340	1340
Putative TldD family protease	General function prediction only	Cytoplasmic		1400	1400
xerD	Replication, recombination and repair	Cytoplasmic		899	899
mscL	Cell wall/membrane/envelope biogenesis	InnerMembrane		407	410
recJ	Replication, recombination and repair	Cytoplasmic		1730	1730
nqrE	Energy production and conversion	InnerMembrane		599	599
lipA	Coenzyme transport and metabolism	Cytoplasmic		965	965
UPF0250 protein NCTC12917_01057	Signal transduction mechanisms	Cytoplasmic		263	263
Serine-type D-Ala-D-Ala carboxypeptidase	Cell wall/membrane/envelope biogenesis	Periplasmic		1190	1196
mltB	Cell wall/membrane/envelope biogenesis	Periplasmic		998	998
mrdA	Cell cycle control, cell division, chromosome partitioning	Periplasmic		1916	1916
rlmH	Translation, ribosomal structure and biogenesis	Cytoplasmic		467	467
rsfS	Translation, ribosomal structure and biogenesis	Cytoplasmic		341	341
holA	Replication, recombination and repair	Cytoplasmic		1031	1031
LPS-assembly lipoprotein LptE	Cell wall/membrane/envelope biogenesis	Periplasmic		482	482
leuS	Translation, ribosomal structure and biogenesis	Cytoplasmic		2579	2579
Zinc ribbon-containing protein	Cell wall/membrane/envelope biogenesis	Cytoplasmic		488	488
lnt	Cell wall/membrane/envelope biogenesis	InnerMembrane		1496	1544

Magnesium and cobalt efflux protein CorC	Inorganic ion transport and metabolism	Cytoplasmic		884	884
ybeY	Translation, ribosomal structure and biogenesis	Cytoplasmic		464	464
PhoH family protein	Signal transduction mechanisms	Cytoplasmic		1070	1070
miaB	Translation, ribosomal structure and biogenesis	Cytoplasmic		1433	1433
group_169	Coenzyme transport and metabolism	Cytoplasmic		146	566
group_951	General function prediction only	Periplasmic		602	602
Glycerate dehydrogenase	Energy production and conversion	Cytoplasmic		956	956
DUF3530 family protein	Unknown protein	Periplasmic		752	752
efp	Translation, ribosomal structure and biogenesis	Cytoplasmic		566	566
guaC	Nucleotide transport and metabolism	Cytoplasmic		1043	1043
ATP-dependent Zn proteases	Function unknown	Periplasmic		497	506
Sensor protein QseC	Signal transduction mechanisms	InnerMembrane		1439	1439
group_6122	Signal transduction mechanisms	Cytoplasmic		689	689
group_957	Cell motility	Periplasmic		638	638
Putative dehydrogenase	General function prediction only	Cytoplasmic		845	845
Biliverdin-producing heme oxygenase	Unknown protein	Cytoplasmic		659	659
AMP-binding domain-containing protein	Lipid transport and metabolism	Cytoplasmic		1466	1466
AHA_3217	Unknown protein	Cytoplasmic		644	644
DUF1006 family protein YcaQ	Replication, recombination and repair	Cytoplasmic		1220	1262
Fumarate reductase subunit D	Energy production and conversion	InnerMembrane		356	356
Fumarate reductase subunit C	Energy production and conversion	InnerMembrane		398	398
Fumarate reductase iron-sulfur subunit	Energy production and conversion	Cytoplasmic		734	734
frdA	Energy production and conversion	Cytoplasmic		1784	1784
group_962	Signal transduction mechanisms	InnerMembrane		1877	1877
Miniconductance mechanosensitive channel MscM	Cell wall/membrane/envelope biogenesis	InnerMembrane		3278	3278
Mate efflux family protein	Defense mechanisms	InnerMembrane		1331	1334
Exported peptidase	Cell wall/membrane/envelope biogenesis	Periplasmic		824	824
group_965	Transcription	Cytoplasmic		899	899
Lactamase_B domain-containing protein	General function prediction only	Periplasmic		860	860
group_967	Transcription	Cytoplasmic		896	896
DD-transpeptidase	Cell wall/membrane/envelope biogenesis	InnerMembrane		2489	2612
tapO	Cell motility	Cytoplasmic		599	599
tapP	Cell motility	Periplasmic		530	530
aroK	Amino acid transport and metabolism	Cytoplasmic		518	518
aroB	Amino acid transport and metabolism	Cytoplasmic		1082	1082
Site-specific DNA-methyltransferase (adenine-specific)	Replication, recombination and repair	Cytoplasmic		872	872

rpe	Carbohydrate transport and metabolism	Cytoplasmic		674	674
gph	Energy production and conversion	Cytoplasmic		668	668
trpS	Translation, ribosomal structure and biogenesis	Cytoplasmic		1004	1004
group_972	Unknown protein	Cytoplasmic		215	215
glutamine amidotransferase component II	Amino acid transport and metabolism	Cytoplasmic		581	581
astC	Amino acid transport and metabolism	Cytoplasmic		1217	1217
astA	Amino acid transport and metabolism	Cytoplasmic		1019	1019
astD	Lipid transport and metabolism	Cytoplasmic		1469	1469
Protein of unknown function (DUF1622)	Function unknown	Cytoplasmic		401	401
ASCH domain-containing protein	Function unknown	Cytoplasmic		317	338
group_977	Signal transduction mechanisms	Cytoplasmic		671	671
Sodium:alanine symporter	Amino acid transport and metabolism	InnerMembrane		1376	1376
Ornithine decarboxylase	Amino acid transport and metabolism	Cytoplasmic		1151	1151
Putative phenylacetate-CoA ligase	Coenzyme transport and metabolism	Cytoplasmic		926	926
group_6327	Unknown protein	Cytoplasmic		215	215
group_1012	Defense mechanisms	Cytoplasmic		881	881
Peptidyl-prolyl cis-trans isomerase	Posttranslational modification, protein turnover, chaperones	Cytoplasmic		611	611
Copper homeostasis protein CutC	Inorganic ion transport and metabolism	Cytoplasmic		725	725
N-acetyl-D-glucosamine kinase	Carbohydrate transport and metabolism	Cytoplasmic		902	902
group_1010	Amino acid transport and metabolism	Cytoplasmic		506	506
pstS	Inorganic ion transport and metabolism	Periplasmic		974	974
phoR	Signal transduction mechanisms	InnerMembrane		1295	1295
phoB	Signal transduction mechanisms	Cytoplasmic		689	689
VOC domain-containing protein	Secondary metabolites biosynthesis, transport and catabolism	Cytoplasmic		410	410
Caib/baif family protein	Lipid transport and metabolism	Cytoplasmic		1154	1154
Glutaryl-CoA dehydrogenase	Lipid transport and metabolism	Cytoplasmic		1175	1175
group_6656	Lipid transport and metabolism	Cytoplasmic		1517	1517
group_6207	Transcription	Cytoplasmic		692	692
group_1005	Signal transduction mechanisms	InnerMembrane		1568	1568
Malic enzyme (NAD)	Energy production and conversion	Cytoplasmic		1550	1550
Citrate-sodium symporter	Energy production and conversion	InnerMembrane		1307	1307
HTH marR-type domain-containing protein	Transcription	Cytoplasmic		473	473
HlyD family secretion protein	Defense mechanisms	Periplasmic		1055	1055
group_1002	Function unknown	InnerMembrane		1028	1028
MliC domain-containing protein	Cell wall/membrane/envelope biogenesis	Periplasmic		359	359
TonB_C domain-containing protein	Unknown protein	Periplasmic		392	392

Conserved domain protein	Energy production and conversion	InnerMembrane		170	170
nrdD	Nucleotide transport and metabolism	Cytoplasmic		2117	2117
ligA	Replication, recombination and repair	Cytoplasmic		2006	2006
Chromosome partition protein Smc	Cell cycle control, cell division, chromosome partitioning	Cytoplasmic		3374	3374
Sulfate transporter CysZ	Amino acid transport and metabolism	InnerMembrane		764	764
Inner membrane protein YadS	Function unknown	InnerMembrane		614	614
DUF3862 domain-containing protein	Unknown protein	Cytoplasmic		251	251
Nonpeptidase	Function unknown	Periplasmic		545	545
Murein L	Cell wall/membrane/envelope biogenesis	Cytoplasmic		1484	1484
group_6011	Unknown protein	Periplasmic		545	545
Bactoprenol glucosyl transferase	Cell wall/membrane/envelope biogenesis	Cytoplasmic		1061	1061
GtrA domain-containing protein	Lipid transport and metabolism	InnerMembrane		371	371
group_996	Carbohydrate transport and metabolism	InnerMembrane		1163	1163
group_995	Carbohydrate transport and metabolism	Periplasmic		2108	2108
GST N-terminal domain-containing protein	Posttranslational modification, protein turnover, chaperones	Cytoplasmic		626	626
Positive regulator AgmR	Signal transduction mechanisms	Cytoplasmic		644	644
HTH tetR-type domain-containing protein	Unknown protein	Cytoplasmic		581	581
group_993	Lipid transport and metabolism	Cytoplasmic		869	869
group_992	General function prediction only	Cytoplasmic		1145	1145
Kef-type K+ transport system	Inorganic ion transport and metabolism	InnerMembrane		1952	1952
GGDEF domain protein	Signal transduction mechanisms	Cytoplasmic		920	935
group_989	Unknown protein	Periplasmic		437	437
group_988	General function prediction only	Cytoplasmic		1733	1739
L-methionine/branched-chain amino acid transporter	Amino acid transport and metabolism	InnerMembrane		1280	1280
Serine transport-like protein asoA	Amino acid transport and metabolism	InnerMembrane		1295	1295
Pyridoxal kinase PdxY	Coenzyme transport and metabolism	Cytoplasmic		863	866
Lysine decarboxylase	Amino acid transport and metabolism	Cytoplasmic		2132	2132
cadB	Amino acid transport and metabolism	InnerMembrane		377	1334
accA	Lipid transport and metabolism	Cytoplasmic		950	950
dnaE	Replication, recombination and repair	Cytoplasmic		3479	3479
lpxB	Cell wall/membrane/envelope biogenesis	Cytoplasmic		1139	1139
lpxA	Cell wall/membrane/envelope biogenesis	Cytoplasmic		791	791
fabZ	Lipid transport and metabolism	Cytoplasmic		422	422
lpxD	Cell wall/membrane/envelope biogenesis	Cytoplasmic		1019	1019
Chaperone protein Skp	Cell wall/membrane/envelope biogenesis	Periplasmic		533	533
rseP	Posttranslational modification, protein turnover, chaperones	InnerMembrane		1352	1352

dxr	Lipid transport and metabolism	Cytoplasmic		1193	1193
Phosphatidate cytidylyltransferase	Lipid transport and metabolism	InnerMembrane		857	857
frr	Translation, ribosomal structure and biogenesis	Cytoplasmic		557	557
pyrH	Nucleotide transport and metabolism	Cytoplasmic		734	734
tsf	Translation, ribosomal structure and biogenesis	Cytoplasmic		881	881
rpsB	Translation, ribosomal structure and biogenesis	Cytoplasmic		728	728
map	Translation, ribosomal structure and biogenesis	Cytoplasmic		788	788
glnD	Posttranslational modification, protein turnover, chaperones	Cytoplasmic		2636	2654
dapD	Amino acid transport and metabolism	Cytoplasmic		827	827
Aminotransferase	Amino acid transport and metabolism	Cytoplasmic		1190	1190
group_1548	Unknown protein	Cytoplasmic		941	941
ATP-dependent DNA helicase DinG	Replication, recombination and repair	Cytoplasmic		2072	2072
DUF2057 domain-containing protein	Function unknown	Periplasmic		659	659
ylqF	Translation, ribosomal structure and biogenesis	Cytoplasmic		950	950
Alkaline phosphatase	Inorganic ion transport and metabolism	Periplasmic		1364	1391
Nucleoside diphosphate kinase regulator	Transcription	Cytoplasmic		395	395
group_5863	Signal transduction mechanisms	Cytoplasmic		590	590
Histidine kinase domain-containing protein	Signal transduction mechanisms	InnerMembrane		1520	1520
group_1512	Amino acid transport and metabolism	Cytoplasmic		791	794
Sterol desaturase	Lipid transport and metabolism	InnerMembrane		839	839
Arginine/ornithine antiporter	Amino acid transport and metabolism	InnerMembrane		1400	1400
group_1509	Unknown protein	Periplasmic		542	542
hlyA	General function prediction only	InnerMembrane		1319	1319
phoU	Inorganic ion transport and metabolism	Cytoplasmic		710	710
pstB	Inorganic ion transport and metabolism	Cytoplasmic		818	818
pstA	Inorganic ion transport and metabolism	InnerMembrane		1649	1649
group_1508	General function prediction only	InnerMembrane		2237	2237
aceB	Energy production and conversion	Cytoplasmic		1607	1607
aceA	Energy production and conversion	Cytoplasmic		1313	1313
hda	Replication, recombination and repair	Cytoplasmic		743	797
DUF2066 domain-containing protein	Function unknown	Periplasmic		1019	1019
upp	Nucleotide transport and metabolism	Cytoplasmic		626	626
purM	Nucleotide transport and metabolism	Cytoplasmic		1037	1037
Nif3-like dinuclear metal center hexameric protein	Coenzyme transport and metabolism	Cytoplasmic		761	761
fleR/flrC	Signal transduction mechanisms	Cytoplasmic		1343	1343
flrB	Signal transduction mechanisms	Periplasmic		1034	1034

flrA	Signal transduction mechanisms	Cytoplasmic		1418	1421
Glycine cleavage system transcriptional repressor	Amino acid transport and metabolism	Cytoplasmic		512	545
ppk1	Inorganic ion transport and metabolism	Cytoplasmic		2069	2069
mgtE	Inorganic ion transport and metabolism	InnerMembrane		1343	1343
flgJ	Cell wall/membrane/envelope biogenesis	Periplasmic		1091	1091
flgF	Cell motility	Periplasmic		746	746
flgC	Cell motility	Periplasmic		419	419
cheV	Signal transduction mechanisms	Cytoplasmic		911	911
flgN	Cell motility	Cytoplasmic		413	413
Phosphoesterase	General function prediction only	Cytoplasmic		542	542
group_6053	Amino acid transport and metabolism	Periplasmic		1637	1637
group_6197	Carbohydrate transport and metabolism	InnerMembrane		905	905
Pseudouridine synthase	Translation, ribosomal structure and biogenesis	Cytoplasmic		692	692
group_6239	Signal transduction mechanisms	Cytoplasmic		1196	1196
group_6699	Replication, recombination and repair	Cytoplasmic		188	188
fsa	Carbohydrate transport and metabolism	Cytoplasmic		650	650
Pyruvate formate-lyase 2-activating enzyme	Posttranslational modification, protein turnover, chaperones	Cytoplasmic		896	896
Formate acetyltransferase 3	Energy production and conversion	Cytoplasmic		2432	2432
HTH deoR-type domain-containing protein	Transcription	Cytoplasmic		746	746
group_6390	Carbohydrate transport and metabolism	InnerMembrane		890	890
group_130	Amino acid transport and metabolism	Periplasmic		1745	1850
DUF2750 domain-containing protein	Unknown protein	Cytoplasmic		392	392
group_461	Unknown protein	Cytoplasmic		557	557
TIGR02647 family protein	Unknown protein	Cytoplasmic		248	248
group_6030	Unknown protein	Periplasmic		305	305
Cold shock domain-containing protein	Transcription	Cytoplasmic		209	209
component of pyruvate dehydrogenase complex	Energy production and conversion	Cytoplasmic		1100	1100
Transket_pyr domain-containing protein	Energy production and conversion	Cytoplasmic		986	986
pdhA	Energy production and conversion	Cytoplasmic		1091	1091
astE	Amino acid transport and metabolism	Cytoplasmic		1025	1025
DUF1127 domain-containing protein	Function unknown	Cytoplasmic		209	209
DNA replication terminus site-binding protein	Unknown protein	Cytoplasmic		893	893
SNF2 family helicase	Transcription	Cytoplasmic		3842	3842
UPF0502 protein AHA_2872	Function unknown	Cytoplasmic		653	653
Ligase	Cell wall/membrane/envelope biogenesis	InnerMembrane		1721	1730
Xanthine dehydrogenase	Energy production and conversion	Periplasmic		485	485

group_298	Energy production and conversion	Cytoplasmic		878	878
group_6381	Energy production and conversion	Cytoplasmic		2318	2318
Permease	Nucleotide transport and metabolism	InnerMembrane		1403	1421
guaD	Nucleotide transport and metabolism	Cytoplasmic		1316	1316
group_5853	Nucleotide transport and metabolism	InnerMembrane		1517	1517
Ald_Xan_dh_C domain-containing protein	Energy production and conversion	Cytoplasmic		2897	2897
ssnA	Nucleotide transport and metabolism	Cytoplasmic		1328	1328
ygfK	Amino acid transport and metabolism	Cytoplasmic		3110	3122
NTP transferase domain-containing protein	Coenzyme transport and metabolism	Cytoplasmic		515	515
yqeC	Unknown protein	Cytoplasmic		848	848
Uncharacterized protein YqeB	Posttranslational modification, protein turnover, chaperones	Cytoplasmic		1592	1592
Sigma-54 factor interaction domain-containing protein	Transcription	Cytoplasmic		1934	1934
Auxin Efflux Carrier	General function prediction only	InnerMembrane		548	884
YaeQ family protein	Function unknown	Cytoplasmic		557	557
TIGR02808 family protein	Unknown protein	Cytoplasmic		140	140
fadJ	Lipid transport and metabolism	Cytoplasmic		2120	2147
fadI	Lipid transport and metabolism	Cytoplasmic		1310	1310
MoxR-like ATPase	General function prediction only	Cytoplasmic		950	950
DUF58 domain-containing protein	Function unknown	Cytoplasmic		890	890
group_308	Transcription	Cytoplasmic		590	590
Protein of unknown function (DUF3379)	Unknown protein	Cytoplasmic		743	746
MerR family DNA-binding transcriptional regulator	Transcription	Cytoplasmic		395	395
ppnP	Nucleotide transport and metabolism	Cytoplasmic		284	284
group_6765	Amino acid transport and metabolism	Cytoplasmic		1376	1382
L-serine dehydratase	Amino acid transport and metabolism	Cytoplasmic		1370	1370
YcgL domain-containing protein AHA_2135	Function unknown	Cytoplasmic		188	281
minC	Cell cycle control, cell division, chromosome partitioning	Cytoplasmic		710	722
minD	Cell cycle control, cell division, chromosome partitioning	Cytoplasmic		812	812
minE	Cell cycle control, cell division, chromosome partitioning	Cytoplasmic		266	266
YciU family protein	Function unknown	Cytoplasmic		326	326
mtgA	Cell wall/membrane/envelope biogenesis	InnerMembrane		707	782
group_6571	Function unknown	Cytoplasmic		323	323
group_315	Function unknown	Cytoplasmic		1214	1214
group_6382	Transcription	Cytoplasmic		470	470
DUF4250 domain-containing protein	Translation, ribosomal structure and biogenesis	Cytoplasmic		188	188
azoR	Energy production and conversion	Cytoplasmic		587	587

DMT superfamily transport protein (Probable substrate riboflavin)	Carbohydrate transport and metabolism	InnerMembrane		842	926
purT	Nucleotide transport and metabolism	Cytoplasmic		1178	1178
Hexitol phosphatase HxpB	Carbohydrate transport and metabolism	Cytoplasmic		659	659
CPXCG motif-containing cysteine-rich protein	Unknown protein	Cytoplasmic		203	203
ribE	Coenzyme transport and metabolism	Cytoplasmic		611	617
UPF0253 protein AHA_2115	Unknown protein	Cytoplasmic		206	206
DNA polymerase	Replication, recombination and repair	Cytoplasmic		2429	2585
tRNA_edit domain-containing protein	General function prediction only	Cytoplasmic		491	494
msbB	Lipid transport and metabolism	InnerMembrane		989	1022
Tellurite resistance protein	Defense mechanisms	InnerMembrane		980	983
group_323	Transcription	Cytoplasmic		908	908
nfo	Replication, recombination and repair	Cytoplasmic		845	845
Beta-glucosidase	Carbohydrate transport and metabolism	Cytoplasmic		1433	1433
group_230	Transcription	Cytoplasmic		179	968
group_1572	General function prediction only	Cytoplasmic		638	638
DLH domain-containing protein	Signal transduction mechanisms	Cytoplasmic		929	929
group_1570	Signal transduction mechanisms	Cytoplasmic		917	917
group_1569	Transcription	Cytoplasmic		920	920
Peptidase_M3 domain-containing protein	Amino acid transport and metabolism	Cytoplasmic		1847	1874
Lytic polysaccharide monooxygenase	General function prediction only	Periplasmic		1424	1424
CysB family HTH-type transcriptional regulator	Transcription	Cytoplasmic		947	947
cysA	Inorganic ion transport and metabolism	Cytoplasmic		1067	1067
cysW	Inorganic ion transport and metabolism	InnerMembrane		896	896
cysT	Inorganic ion transport and metabolism	InnerMembrane		839	839
Alpha/beta hydrolase	General function prediction only	Cytoplasmic		575	575
ABC-type sulfate transport system	Inorganic ion transport and metabolism	Periplasmic		1001	1001
DUF4010 domain-containing protein	Inorganic ion transport and metabolism	InnerMembrane		1262	1262
Mac domain-containing protein	General function prediction only	Cytoplasmic		590	590
group_697	Unknown protein	Cytoplasmic		212	212
argH	Amino acid transport and metabolism	Cytoplasmic		1382	1382
argG	Amino acid transport and metabolism	Cytoplasmic		1235	1235
argF	Amino acid transport and metabolism	Cytoplasmic		911	911
argB	Amino acid transport and metabolism	Cytoplasmic		779	779
argC	Amino acid transport and metabolism	Periplasmic		1007	1007
argE	Amino acid transport and metabolism	Cytoplasmic		1145	1145
ppc	Energy production and conversion	Cytoplasmic		2633	2633

aspartokinase/homoserine dehydrogenase	Amino acid transport and metabolism	Cytoplasmic		2468	2468
metB	Amino acid transport and metabolism	Cytoplasmic		1163	1172
Met regulon regulatory protein MetJ	Transcription	Cytoplasmic		323	323
csaA	Translation, ribosomal structure and biogenesis	Cytoplasmic		338	338
Serine transporter	Amino acid transport and metabolism	InnerMembrane		1271	1271
cysQ	Inorganic ion transport and metabolism	Cytoplasmic		812	812
group_6138	Defense mechanisms	Cytoplasmic		608	608
GMP/IMP nucleotidase	Coenzyme transport and metabolism	Cytoplasmic		668	731
HTH luxR-type domain-containing protein	Signal transduction mechanisms	InnerMembrane		455	815
exeN	Unknown protein	Periplasmic		755	818
exeM	Intracellular trafficking, secretion, and vesicular transport	Cytoplasmic		491	491
exeL	Intracellular trafficking, secretion, and vesicular transport	Cytoplasmic		1184	1202
exeK	Intracellular trafficking, secretion, and vesicular transport	Periplasmic		1013	1013
exeJ	Intracellular trafficking, secretion, and vesicular transport	InnerMembrane		623	701
exeI	Cell motility	Periplasmic		359	359
exeH	Cell motility	Cytoplasmic		551	563
exeG	Cell motility	Periplasmic		431	431
exeF	Cell motility	InnerMembrane		1220	1220
exeE	Cell motility	Cytoplasmic		1505	1505
exeC	Intracellular trafficking, secretion, and vesicular transport	Periplasmic		848	848
Heat shock protein 15	Translation, ribosomal structure and biogenesis	Periplasmic		398	398
hslO	Posttranslational modification, protein turnover, chaperones	Cytoplasmic		884	884
pckA	Energy production and conversion	Cytoplasmic		1625	1625
group_6141	Transcription	Cytoplasmic		665	665
Xaa-Pro aminopeptidase	Amino acid transport and metabolism	Cytoplasmic		1802	1802
Nitrous oxide-stimulated promoter	Unknown protein	Cytoplasmic		404	404
cpdB	Nucleotide transport and metabolism	Periplasmic		1967	1979
yggA	Amino acid transport and metabolism	InnerMembrane		620	620
asaR	Transcription	Cytoplasmic		782	782
asaI	Signal transduction mechanisms	Cytoplasmic		623	623
argP	Transcription	Cytoplasmic		899	899
rnb	Transcription	Cytoplasmic		2021	2021
group_1295	Transcription	Cytoplasmic		593	593
DUF418 domain-containing protein	Function unknown	InnerMembrane		1046	1046
mtlA	Carbohydrate transport and metabolism	InnerMembrane		1922	1958
mtlD	Carbohydrate transport and metabolism	Cytoplasmic		1145	1145

DNA-binding repressor	Transcription	Cytoplasmic		521	521
Sodium:proton exchanger	Inorganic ion transport and metabolism	InnerMembrane		1787	1787
Small-conductance mechanosensitive channel	Cell wall/membrane/envelope biogenesis	InnerMembrane		518	518
zntB	Inorganic ion transport and metabolism	InnerMembrane		965	965
UPF0178 protein AHA_0543	Function unknown	Cytoplasmic		452	452
group_1300	Function unknown	Periplasmic		269	269
Nucleoside permease NupC	Nucleotide transport and metabolism	InnerMembrane		1205	1205
PepSY domain-containing protein	Function unknown	InnerMembrane		1397	1397
Family of unknown function (DUF6500)	Unknown protein	Cytoplasmic		215	215
menA	Coenzyme transport and metabolism	InnerMembrane		905	905
Isochorismate synthase MenF	Coenzyme transport and metabolism	Cytoplasmic		1346	1346
menD	Coenzyme transport and metabolism	InnerMembrane		1730	1730
menB	Coenzyme transport and metabolism	Cytoplasmic		860	860
menC	Coenzyme transport and metabolism	Cytoplasmic		929	929
fimD	Extracellular structures	Periplasmic		731	731
AbiTii domain-containing protein	Unknown protein	Cytoplasmic		527	527
4HBT domain-containing protein	Secondary metabolites biosynthesis, transport and catabolism	Cytoplasmic		455	455
recQ	Replication, recombination and repair	Cytoplasmic		1835	1835
Cytochrome c domain-containing protein	Energy production and conversion	Periplasmic		299	299
selO	Posttranslational modification, protein turnover, chaperones	Cytoplasmic		1427	1427
Probable queuosine precursor transporter	Translation, ribosomal structure and biogenesis	InnerMembrane		674	674
rraA	Translation, ribosomal structure and biogenesis	Cytoplasmic		485	485
group_350	Signal transduction mechanisms	InnerMembrane		1853	1853
cysG3	Coenzyme transport and metabolism	Cytoplasmic		1406	1406
Nitrite transporter NirC	Inorganic ion transport and metabolism	InnerMembrane		809	812
nirB	Energy production and conversion	Cytoplasmic		2546	2546
hslU	Posttranslational modification, protein turnover, chaperones	Cytoplasmic		1328	1328
hslV	Posttranslational modification, protein turnover, chaperones	Cytoplasmic		533	533
SPOR domain-containing protein	Cell cycle control, cell division, chromosome partitioning	Periplasmic		839	839
argS	Translation, ribosomal structure and biogenesis	Cytoplasmic		1745	1745
Lon N-terminal domain-containing protein	Function unknown	Cytoplasmic		566	566
RNA polymerase sigma factor	Transcription	Cytoplasmic		569	569
Cupin_7 domain-containing protein	Signal transduction mechanisms	Cytoplasmic		665	665
uppP	Lipid transport and metabolism	InnerMembrane		815	815
Aldo_ket_red domain-containing protein	Coenzyme transport and metabolism	Periplasmic		1010	1010
galT	Carbohydrate transport and metabolism	Cytoplasmic		1046	1058

galE	Cell wall/membrane/envelope biogenesis	Cytoplasmic		1013	1013
group_338	Transcription	Cytoplasmic		1004	1082
D-galactose-binding periplasmic protein	Carbohydrate transport and metabolism	Periplasmic		986	986
Galactose/methyl galactoside import ATP-binding protein MglA	Carbohydrate transport and metabolism	Cytoplasmic		1475	1544
mglC	Carbohydrate transport and metabolism	InnerMembrane		1010	1010
group_336	Signal transduction mechanisms	InnerMembrane		1733	1733
CopG family transcriptional regulator	Unknown protein	Cytoplasmic		368	368
CbiA domain-containing protein	Cell cycle control, cell division, chromosome partitioning	Cytoplasmic		647	647
arcA	Amino acid transport and metabolism	Cytoplasmic		1220	1220
arcC	Amino acid transport and metabolism	Cytoplasmic		926	926
group_6140	Amino acid transport and metabolism	Cytoplasmic		1004	1004
YfcC family protein	General function prediction only	InnerMembrane		1403	1406
Arginine repressor	Transcription	Cytoplasmic		491	491
pyrI	Nucleotide transport and metabolism	Cytoplasmic		464	464
pyrB	Nucleotide transport and metabolism	Cytoplasmic		920	920
Methyl-accepting chemotaxis protein	Signal transduction mechanisms	InnerMembrane		383	1991
Outer membrane lipoprotein Blc	Cell wall/membrane/envelope biogenesis	Periplasmic		512	527
group_6248	Transcription	Cytoplasmic		902	902
Alcohol dehydrogenase	Energy production and conversion	Cytoplasmic		1163	1163
pntA	Energy production and conversion	InnerMembrane		1526	1526
pntB	Energy production and conversion	InnerMembrane		1484	1484
UPF0597 protein AHA_4077	Amino acid transport and metabolism	InnerMembrane		1292	1292
Sodium: dicarboxylate symporter	Amino acid transport and metabolism	InnerMembrane		1400	1400
cdr	Lipid transport and metabolism	Cytoplasmic		491	1328
bamD	Cell wall/membrane/envelope biogenesis	Cytoplasmic		755	755
rluD	Translation, ribosomal structure and biogenesis	Cytoplasmic		980	980
clpV	Posttranslational modification, protein turnover, chaperones	Cytoplasmic		2573	2573
asnA	Amino acid transport and metabolism	Cytoplasmic		992	992
group_329	Signal transduction mechanisms	InnerMembrane		1400	1400
group_6409	Unknown protein	Periplasmic		488	488
Protein-methionine-sulfoxide reductase catalytic subunit MsrP	Energy production and conversion	Cytoplasmic		1001	1001
Protein-methionine-sulfoxide reductase heme-binding subunit MsrQ	Energy production and conversion	InnerMembrane		632	632
group_5848	Posttranslational modification, protein turnover, chaperones	Cytoplasmic		239	239
group_5688	Posttranslational modification, protein turnover, chaperones	Cytoplasmic		332	332
group_6130	Signal transduction mechanisms	Cytoplasmic		656	656
group_5997	Signal transduction mechanisms	InnerMembrane		1349	1349

group_5784	Unknown protein	Cytoplasmic		461	461
torA	Energy production and conversion	Periplasmic		2480	2480
group_6473	Energy production and conversion	Periplasmic		1130	1130
topB	Replication, recombination and repair	Cytoplasmic		1967	2120
MC21 protein	Defense mechanisms	InnerMembrane		434	434
Alkylhydroperoxidase AhpD domain protein	Inorganic ion transport and metabolism	Periplasmic		533	533
group_1453	Transcription	InnerMembrane		827	827
TrkA family potassium uptake protein	Inorganic ion transport and metabolism	Cytoplasmic		653	653
Bifunctional ligase/repressor BirA	Coenzyme transport and metabolism	Cytoplasmic		965	965
coaA	Coenzyme transport and metabolism	Cytoplasmic		938	938
Pullulanase	Unknown protein	Periplasmic		449	452
PINc domain-containing protein	General function prediction only	Cytoplasmic		1370	1370
thiQ	Coenzyme transport and metabolism	InnerMembrane		707	707
leuD	Amino acid transport and metabolism	Cytoplasmic		599	599
leuC	Amino acid transport and metabolism	Cytoplasmic		1397	1397
leuB	Energy production and conversion	Cytoplasmic		1085	1085
group_6124	Amino acid transport and metabolism	Cytoplasmic		1574	1574
group_5876	Transcription	Cytoplasmic		623	623
acidPPc domain-containing protein	Lipid transport and metabolism	InnerMembrane		500	503
group_6719	Unknown protein	Cytoplasmic		152	152
ilvB	Amino acid transport and metabolism	Periplasmic		1718	1718
ilvN	Amino acid transport and metabolism	Cytoplasmic		494	494
pta	Energy production and conversion	Cytoplasmic		2138	2171
group_6224	Energy production and conversion	Cytoplasmic		1202	1202
UPF0208 membrane protein YfbV	Function unknown	InnerMembrane		440	440
YfbU domain-containing protein	Function unknown	Cytoplasmic		500	500
Thioredoxin peroxidase	Posttranslational modification, protein turnover, chaperones	Cytoplasmic		464	464
group_1394	Amino acid transport and metabolism	Cytoplasmic		533	626
4-hydroxy-tetrahydrodipicolinate synthase	Amino acid transport and metabolism	Cytoplasmic		887	887
Outer membrane protein assembly factor BamC	Cell wall/membrane/envelope biogenesis	Periplasmic		1025	1025
purC	Nucleotide transport and metabolism	Cytoplasmic		1103	1139
DUF3334 family protein	Unknown protein	Cytoplasmic		698	698
Aerobic respiration control sensor protein	Signal transduction mechanisms	Cytoplasmic		2312	2312
group_1389	Unknown protein	Cytoplasmic		203	203
Response regulator	Signal transduction mechanisms	Cytoplasmic		1211	1211
mutL	Replication, recombination and repair	Cytoplasmic		1868	1871

tRNA dimethylallyltransferase	Translation, ribosomal structure and biogenesis	Cytoplasmic		926	926
hfq	Signal transduction mechanisms	Cytoplasmic		263	263
hflX	Translation, ribosomal structure and biogenesis	Cytoplasmic		1286	1286
hflK	Posttranslational modification, protein turnover, chaperones	Cytoplasmic		1151	1151
hflC	Posttranslational modification, protein turnover, chaperones	Cytoplasmic		884	884
DUF2065 domain-containing protein	Function unknown	Cytoplasmic		188	188
Octaprenyl-diphosphate synthase	Coenzyme transport and metabolism	Cytoplasmic		971	971
rplU	Translation, ribosomal structure and biogenesis	Cytoplasmic		311	311
rpmA	Translation, ribosomal structure and biogenesis	Cytoplasmic		257	257
obg	Cell cycle control, cell division, chromosome partitioning	Cytoplasmic		1202	1202
Gate domain-containing protein	Cell cycle control, cell division, chromosome partitioning	InnerMembrane		1370	1370
Long-chain-fatty-acid--CoA ligase	Lipid transport and metabolism	Cytoplasmic		1694	1694
HD domain-containing protein	Signal transduction mechanisms	Cytoplasmic		2888	2888
Bac_luciferase domain-containing protein	Coenzyme transport and metabolism	Cytoplasmic		989	989
Helix-turn-helix domain protein	Translation, ribosomal structure and biogenesis	Cytoplasmic		500	500
apaH	Signal transduction mechanisms	Cytoplasmic		821	821
Co2+/Mg2+ efflux protein ApaG	Inorganic ion transport and metabolism	Cytoplasmic		362	362
rsmA	Translation, ribosomal structure and biogenesis	Cytoplasmic		812	827
4-hydroxythreonine-4-phosphate dehydrogenase	Coenzyme transport and metabolism	Cytoplasmic		995	995
Chaperone SurA	Posttranslational modification, protein turnover, chaperones	Periplasmic		1298	1298
Co-chaperone protein DjlA	Posttranslational modification, protein turnover, chaperones	Cytoplasmic		830	830
Adenine permease	Nucleotide transport and metabolism	InnerMembrane		1343	1343
Energy-dependent translational throttle protein EttA	General function prediction only	Cytoplasmic		1667	1667
group_190	Signal transduction mechanisms	Cytoplasmic		410	575
Inner membrane protein ygjV	Unknown protein	InnerMembrane		506	509
Lytic transglycosylase	Cell wall/membrane/envelope biogenesis	Periplasmic		854	854
Catabolite repressor/activator	Transcription	Cytoplasmic		983	986
fused PTS fructose transporter subunit IIA/HPr protein	Carbohydrate transport and metabolism	Cytoplasmic		1100	1100
PTS system	Carbohydrate transport and metabolism	InnerMembrane		1727	1736
Membrane-bound lytic murein transglycosylase A	Cell wall/membrane/envelope biogenesis	Periplasmic		1151	1151
Ribonucleoside-diphosphate reductase	Nucleotide transport and metabolism	Cytoplasmic		1133	1133
group_837	Energy production and conversion	Cytoplasmic		686	686
ubiG	Coenzyme transport and metabolism	Cytoplasmic		716	716
gyrA	Replication, recombination and repair	Cytoplasmic		2747	2747
group_838	General function prediction only	Cytoplasmic		680	1055
thrS	Translation, ribosomal structure and biogenesis	Cytoplasmic		1928	1928

Translation initiation factor IF-3	Translation, ribosomal structure and biogenesis	Cytoplasmic		407	407
rpmI	Translation, ribosomal structure and biogenesis	Cytoplasmic		197	197
rplT	Translation, ribosomal structure and biogenesis	Cytoplasmic		356	356
pheS	Translation, ribosomal structure and biogenesis	Cytoplasmic		983	983
pheT	Translation, ribosomal structure and biogenesis	Cytoplasmic		2387	2387
ihfA	Replication, recombination and repair	Cytoplasmic		296	296
Response regulatory domain-containing protein	Signal transduction mechanisms	InnerMembrane		1247	1247
NAD-dependent protein deacylase	Posttranslational modification, protein turnover, chaperones	Cytoplasmic		773	773
group_844	Transcription	Cytoplasmic		1004	1004
rbsK	Carbohydrate transport and metabolism	Cytoplasmic		932	932
Peripla_BP_4 domain-containing protein	Carbohydrate transport and metabolism	Periplasmic		875	878
rbsC	Carbohydrate transport and metabolism	InnerMembrane		965	965
Ribose import ATP-binding protein RbsA	Carbohydrate transport and metabolism	Cytoplasmic		1523	1532
rbsD	Carbohydrate transport and metabolism	Cytoplasmic		419	419
Glutathione S-transferase family protein	Posttranslational modification, protein turnover, chaperones	Cytoplasmic		617	617
group_849	Function unknown	InnerMembrane		737	737
DUF2987 domain-containing protein	Unknown protein	Periplasmic		677	677
ttcA	Translation, ribosomal structure and biogenesis	Cytoplasmic		908	908
Universal stress protein E	Signal transduction mechanisms	Cytoplasmic		944	944
FNR family transcription factor	Signal transduction mechanisms	Cytoplasmic		755	755
DsbD_2 domain-containing protein	Function unknown	InnerMembrane		686	686
ccoS	Posttranslational modification, protein turnover, chaperones	Cytoplasmic		191	191
cadA	Inorganic ion transport and metabolism	InnerMembrane		2399	2399
Cytochrome C oxidase Cbb3	Function unknown	Periplasmic		488	488
ccoP	Energy production and conversion	Periplasmic		989	989
cytochrome c oxidase assembly chaperone	Energy production and conversion	Periplasmic		179	179
ccoO	Energy production and conversion	Cytoplasmic		614	614
ccoN	Energy production and conversion	InnerMembrane		1424	1424
GAF domain-containing protein	Defense mechanisms	Cytoplasmic		458	458
proQ	Signal transduction mechanisms	Periplasmic		638	638
C-terminal processing peptidase	Posttranslational modification, protein turnover, chaperones	Cytoplasmic		2015	2015
DUF2835 domain-containing protein	Unknown protein	Cytoplasmic		221	221
Glutamate dehydrogenase	Amino acid transport and metabolism	Cytoplasmic		4841	4859
pyrD	Nucleotide transport and metabolism	Cytoplasmic		1010	1010
zapC	Unknown protein	Cytoplasmic		536	536
rlmL	Translation, ribosomal structure and biogenesis	Cytoplasmic		2168	2171

ATP-binding protein Uup	General function prediction only	Cytoplasmic		1922	1922
fabA	Lipid transport and metabolism	Cytoplasmic		560	560
Endopeptidase La	Posttranslational modification, protein turnover, chaperones	Cytoplasmic		1979	1979
3-oxoacyl-ACP reductase	Lipid transport and metabolism	Cytoplasmic		1970	1970
PNPLA domain-containing protein	General function prediction only	Cytoplasmic		1088	1184
group_643	Amino acid transport and metabolism	Periplasmic		2153	2153
group_642	Unknown protein	Cytoplasmic		152	152
Lysoplasmalogenase	Function unknown	InnerMembrane		626	626
Multidrug resistance protein B	Carbohydrate transport and metabolism	InnerMembrane		1547	1553
group_6092	Transcription	Cytoplasmic		902	902
DUF2007 domain-containing protein	Unknown protein	Cytoplasmic		314	353
Proton/glutamate symporter	Energy production and conversion	InnerMembrane		1283	1283
UPF0056 membrane protein	Amino acid transport and metabolism	InnerMembrane		575	1652
group_6459	Transcription	Cytoplasmic		968	968
tatA	Intracellular trafficking, secretion, and vesicular transport	Cytoplasmic		161	161
CHRD domain-containing protein	Function unknown	Cytoplasmic		302	302
group_638	Cell wall/membrane/envelope biogenesis	InnerMembrane		1424	1427
Phospholipase D family protein	Lipid transport and metabolism	InnerMembrane		1253	1253
FA_desaturase domain-containing protein	Lipid transport and metabolism	InnerMembrane		1118	1118
DNA-binding transcriptional regulator FabR	Transcription	Cytoplasmic		620	620
ATPase of the AAA+ class	Unknown protein	InnerMembrane		365	365
DUF1145 domain-containing protein	Function unknown	InnerMembrane		311	311
rsmD	Translation, ribosomal structure and biogenesis	Cytoplasmic		629	629
group_632	Intracellular trafficking, secretion, and vesicular transport	Cytoplasmic		1613	1883
ftsE	Cell cycle control, cell division, chromosome partitioning	Cytoplasmic		668	668
ftsX	Cell cycle control, cell division, chromosome partitioning	InnerMembrane		953	956
rpoH	Transcription	Cytoplasmic		854	854
Carbonic anhydrase	Inorganic ion transport and metabolism	Cytoplasmic		659	659
hutC	Transcription	Cytoplasmic		704	704
hutI	Secondary metabolites biosynthesis, transport and catabolism	Cytoplasmic		1235	1235
hutU	Amino acid transport and metabolism	Cytoplasmic		1709	1709
hutH	Amino acid transport and metabolism	Cytoplasmic		1532	1532
Calcium/proton antiporter	Inorganic ion transport and metabolism	InnerMembrane		1097	1097
Diguanylate cyclase / EAL-type diguanylate phosphodiesterase CsrD	Signal transduction mechanisms	Cytoplasmic		1928	1928
mshM	Intracellular trafficking, secretion, and vesicular transport	InnerMembrane		890	890
mshE	Cell motility	Cytoplasmic		1709	1709

mshG	Cell motility	InnerMembrane		1220	1238
Cell shape-determining protein MreB	Cell cycle control, cell division, chromosome partitioning	Cytoplasmic		1040	1040
mreC	Cell cycle control, cell division, chromosome partitioning	Periplasmic		893	905
mreD	Cell wall/membrane/envelope biogenesis	InnerMembrane		485	485
maf	Secondary metabolites biosynthesis, transport and catabolism	Cytoplasmic		587	587
Ribonuclease G	Translation, ribosomal structure and biogenesis	Cytoplasmic		1469	1469
tldD	General function prediction only	Periplasmic		1448	1448
group_5830	Unknown protein	Cytoplasmic		203	203
UPF0056 inner membrane protein	Amino acid transport and metabolism	InnerMembrane		716	716
group_626	Carbohydrate transport and metabolism	InnerMembrane		1316	1316
Diphthami_syn_2 domain-containing protein	Translation, ribosomal structure and biogenesis	Cytoplasmic		671	674
Response regulator / HD-GYP family diguanylate phosphodiesterase	Signal transduction mechanisms	Cytoplasmic		1130	1130
Class B acid phosphatase	Inorganic ion transport and metabolism	Periplasmic		710	893
Protein YicC	Function unknown	Cytoplasmic		863	863
rph	Translation, ribosomal structure and biogenesis	Cytoplasmic		716	716
pyrE	Nucleotide transport and metabolism	Cytoplasmic		653	653
cueR	Transcription	Cytoplasmic		389	389
group_372	Signal transduction mechanisms	InnerMembrane		1154	1154
Protein YihD	Function unknown	Cytoplasmic		263	263
ccoG	Energy production and conversion	InnerMembrane		1448	1448
Thiol:disulfide interchange protein	Posttranslational modification, protein turnover, chaperones	Periplasmic		608	608
phnD	Inorganic ion transport and metabolism	Periplasmic		845	845
pncB	Coenzyme transport and metabolism	Cytoplasmic		1178	1178
Nicotinamidase	Coenzyme transport and metabolism	Cytoplasmic		635	635
DNA mismatch repair protein MutT	Function unknown	Cytoplasmic		650	650
Acyltransferase	Lipid transport and metabolism	InnerMembrane		914	914
group_365	Amino acid transport and metabolism	Cytoplasmic		1646	1646
Acetolactate synthase	Amino acid transport and metabolism	Cytoplasmic		251	251
ilvE	Amino acid transport and metabolism	Periplasmic		944	944
ilvD	Amino acid transport and metabolism	Cytoplasmic		1841	1841
ilvA	Amino acid transport and metabolism	Cytoplasmic		1520	1520
priA	Replication, recombination and repair	Cytoplasmic		2207	2207
rpmE	Translation, ribosomal structure and biogenesis	Periplasmic		215	215
group_361	General function prediction only	InnerMembrane		1433	1457
Malic enzyme (NADP)	Energy production and conversion	Cytoplasmic		1256	1256
hypothetical protein	Unknown protein	Periplasmic		227	227

group_6607	Signal transduction mechanisms	InnerMembrane		1325	1325
Two-component system response regulator RstA	Signal transduction mechanisms	Cytoplasmic		758	758
trmA	Translation, ribosomal structure and biogenesis	Cytoplasmic		1133	1133
DUF5610 domain-containing protein	Unknown protein	Periplasmic		1142	1142
Cob_adeno_trans domain-containing protein	Coenzyme transport and metabolism	Cytoplasmic		533	533
Maltodextrin glucosidase	Carbohydrate transport and metabolism	Cytoplasmic		1829	1829
HDOD domain-containing protein	Signal transduction mechanisms	Cytoplasmic		830	830
META domain-containing protein	Posttranslational modification, protein turnover, chaperones	Cytoplasmic		419	419
Molecular chaperone	Posttranslational modification, protein turnover, chaperones	Cytoplasmic		1361	1379
2-hydroxyacid dehydrogenase	Energy production and conversion	Cytoplasmic		989	989
Protein CreA	Signal transduction mechanisms	Periplasmic		479	479
DUF962 domain-containing protein	Lipid transport and metabolism	InnerMembrane		446	446
group_354	Unknown protein	Cytoplasmic		857	857
RNA polymerase-binding protein DksA	Transcription	Cytoplasmic		374	374
Glutamine synthetase	Amino acid transport and metabolism	Cytoplasmic		1331	1331
group_6285	Coenzyme transport and metabolism	Cytoplasmic		1361	1361
Putrescine-binding periplasmic protein	Amino acid transport and metabolism	Periplasmic		1097	1097
Spermidine/putrescine import ATP-binding protein PotA	Amino acid transport and metabolism	Cytoplasmic		1154	1154
group_353	Amino acid transport and metabolism	InnerMembrane		905	905
group_6082	Amino acid transport and metabolism	InnerMembrane		842	845
BPL/LPL catalytic domain-containing protein	Coenzyme transport and metabolism	Cytoplasmic		701	701
pbuX	Nucleotide transport and metabolism	InnerMembrane		1397	1397
Diguanylate cyclase	Signal transduction mechanisms	InnerMembrane		1139	1139
Protein YciN	Unknown protein	Cytoplasmic		248	248
group_1348	Defense mechanisms	InnerMembrane		1379	1379
group_1349	Transcription	InnerMembrane		857	857
maeA	Energy production and conversion	Cytoplasmic		1694	1694
group_5772	Transcription	Cytoplasmic		266	266
Transporter	General function prediction only	InnerMembrane		1361	1361
group_1350	General function prediction only	Cytoplasmic		866	866
AcrB/AcrD/AcrF family protein	Defense mechanisms	InnerMembrane		3119	3119
group_1351	Unknown protein	InnerMembrane		368	371
DNA damage-inducible protein DinB	Secondary metabolites biosynthesis, transport and catabolism	Cytoplasmic		533	533
ruvC	Replication, recombination and repair	Cytoplasmic		521	521
Aquaporin Z	Carbohydrate transport and metabolism	InnerMembrane		662	686
SLT domain-containing protein	Cell wall/membrane/envelope biogenesis	Periplasmic		743	743

GNAT family acetyltransferase	Unknown protein	Cytoplasmic		479	479
DUF1439 domain-containing protein	Unknown protein	Periplasmic		542	542
cls	Lipid transport and metabolism	InnerMembrane		1481	1481
purU	Nucleotide transport and metabolism	Cytoplasmic		836	836
group_1356	Coenzyme transport and metabolism	Cytoplasmic		653	734
Protein NlpC	Cell wall/membrane/envelope biogenesis	Cytoplasmic		488	488
group_1357	Cell wall/membrane/envelope biogenesis	Cytoplasmic		917	917
katG	Inorganic ion transport and metabolism	Periplasmic		2186	2207
group_1491	Function unknown	Periplasmic		704	704
Gamma-aminobutyrate permease-like protein	Amino acid transport and metabolism	InnerMembrane		1376	1376
Putative phosphoenolpyruvate synthase regulatory protein	Signal transduction mechanisms	Cytoplasmic		812	812
ppsA	Carbohydrate transport and metabolism	Cytoplasmic		2372	2372
group_1490	Transcription	Cytoplasmic		1013	1013
group_6016	Unknown protein	Periplasmic		458	458
Isochorismatase domain-containing protein	Coenzyme transport and metabolism	Cytoplasmic		539	539
group_5657	Unknown protein	InnerMembrane		374	374
group_1488	Inorganic ion transport and metabolism	Periplasmic		830	830
Phosphatase PAP2 family protein	Lipid transport and metabolism	InnerMembrane		722	722
pdxB	Coenzyme transport and metabolism	Cytoplasmic		1130	1130
group_6351	Amino acid transport and metabolism	Cytoplasmic		1016	1016
accD	Lipid transport and metabolism	Cytoplasmic		863	863
Dihydrofolate synthase/folylpolyglutamate synthase	Coenzyme transport and metabolism	Cytoplasmic		1259	1259
Cell division protein DedD	Cell cycle control, cell division, chromosome partitioning	Periplasmic		800	800
tusE	Translation, ribosomal structure and biogenesis	Cytoplasmic		344	344
yccS	Function unknown	InnerMembrane		2153	2153
group_1693	Cell wall/membrane/envelope biogenesis	InnerMembrane		1616	1625
mprF	Lipid transport and metabolism	InnerMembrane		2549	2549
group_86	Signal transduction mechanisms	Cytoplasmic		386	1664
group_1188	Signal transduction mechanisms	InnerMembrane		1583	1583
Pseudouridine synthase Rlu family protein	Translation, ribosomal structure and biogenesis	Cytoplasmic		659	659
hppD	Amino acid transport and metabolism	Cytoplasmic		1097	1097
Oxidoreductase	Secondary metabolites biosynthesis, transport and catabolism	Cytoplasmic		1160	1160
Fumarylacetoacetase	Secondary metabolites biosynthesis, transport and catabolism	Periplasmic		998	998
asnB	Amino acid transport and metabolism	Cytoplasmic		1664	1664
Glutathione-regulated potassium-efflux system protein KefC	Inorganic ion transport and metabolism	InnerMembrane		1574	1574
HAD-IIA family hydrolase	Nucleotide transport and metabolism	Cytoplasmic		749	749

group_191	Nucleotide transport and metabolism	Cytoplasmic		1019	1064
Oxidored_FMN domain-containing protein	Energy production and conversion	Periplasmic		1121	1121
Putative membrane protein fused with conserved domain	Inorganic ion transport and metabolism	InnerMembrane		1535	1535
cation transporter	Inorganic ion transport and metabolism	InnerMembrane		926	926
group_1193	Energy production and conversion	Cytoplasmic		3056	3059
Esterase YdiI	Secondary metabolites biosynthesis, transport and catabolism	Cytoplasmic		461	461
ATP-dependent RNA helicase DeaD	Replication, recombination and repair	Cytoplasmic		1895	1895
Protoporphyrinogen oxidase	Coenzyme transport and metabolism	Periplasmic		524	524
Trk system potassium uptake protein	Inorganic ion transport and metabolism	InnerMembrane		1457	1457
YigZ family protein	General function prediction only	Cytoplasmic		617	617
pepQ	Amino acid transport and metabolism	Cytoplasmic		1322	1322
Acetoacetyl-CoA synthetase	Lipid transport and metabolism	Cytoplasmic		1937	1937
fadB	Lipid transport and metabolism	Cytoplasmic		2147	2147
fadA	Lipid transport and metabolism	Cytoplasmic		1163	1163
group_6437	Signal transduction mechanisms	Cytoplasmic		722	722
group_6510	Signal transduction mechanisms	InnerMembrane		1445	1445
ABC-type sugar transport system	Carbohydrate transport and metabolism	Periplasmic		1238	1238
group_5707	Unknown protein	Cytoplasmic		191	191
tusA	Translation, ribosomal structure and biogenesis	Cytoplasmic		176	245
Endoribonuclease L-PSP	Defense mechanisms	Cytoplasmic		347	347
tag	Replication, recombination and repair	Cytoplasmic		581	581
glyQ	Translation, ribosomal structure and biogenesis	Cytoplasmic		923	923
Glycine--tRNA ligase beta subunit	Translation, ribosomal structure and biogenesis	Cytoplasmic		2069	2069
group_1410	Unknown protein	Periplasmic		380	380
DM13 domain-containing protein	Unknown protein	Periplasmic		476	479
Low affinity tryptophan permease	Amino acid transport and metabolism	InnerMembrane		1220	1226
HTH gntR-type domain-containing protein	Transcription	Cytoplasmic		707	746
Pts system maltose-specific eiicb component	Carbohydrate transport and metabolism	InnerMembrane		1496	1586
Maltose-6'-phosphate glucosidase	Carbohydrate transport and metabolism	Cytoplasmic		1319	1319
HTH lacI-type domain-containing protein	Transcription	Cytoplasmic		1004	1004
Peripla_BP_6 domain-containing protein	Amino acid transport and metabolism	Periplasmic		1079	1121
Branched-chain amino acid ABC transporter permease	Amino acid transport and metabolism	InnerMembrane		926	926
livM	Amino acid transport and metabolism	InnerMembrane		1268	1271
livG	Amino acid transport and metabolism	Cytoplasmic		773	773
livF	Amino acid transport and metabolism	Cytoplasmic		701	701
group_1403	Transcription	Cytoplasmic		833	833

Lysine transporter LysE	Amino acid transport and metabolism	InnerMembrane		587	587
glpE	Inorganic ion transport and metabolism	Cytoplasmic		323	323
glpG	Posttranslational modification, protein turnover, chaperones	InnerMembrane		833	833
Ubiquinone biosynthesis accessory factor UbiK	Coenzyme transport and metabolism	Cytoplasmic		257	257
fre	Coenzyme transport and metabolism	Cytoplasmic		698	698
rho	Transcription	Cytoplasmic		1265	1265
trxA	Posttranslational modification, protein turnover, chaperones	Cytoplasmic		326	326
rhlB	Replication, recombination and repair	Cytoplasmic		1289	1289
gppA	Nucleotide transport and metabolism	Cytoplasmic		1490	1490
rsmJ	Translation, ribosomal structure and biogenesis	Cytoplasmic		746	746
group_1529	Signal transduction mechanisms	Cytoplasmic		2543	2543
Oligopeptidase A	Posttranslational modification, protein turnover, chaperones	Cytoplasmic		2042	2042
Delta-aminolevulinic acid dehydratase	Coenzyme transport and metabolism	Cytoplasmic		1019	1019
3'-5' ssDNA/RNA exonuclease TatD	Cell motility	Cytoplasmic		785	785
tatC	Intracellular trafficking, secretion, and vesicular transport	InnerMembrane		755	755
group_1534	Intracellular trafficking, secretion, and vesicular transport	Periplasmic		248	251
ubiB	Coenzyme transport and metabolism	InnerMembrane		1640	1640
Ubiquinone biosynthesis accessory factor UbiJ	Coenzyme transport and metabolism	Cytoplasmic		608	608
ubiE	Coenzyme transport and metabolism	Cytoplasmic		752	752
glpC	Energy production and conversion	Periplasmic		1202	1202
glpB	Amino acid transport and metabolism	Cytoplasmic		1280	1280
glpA	Energy production and conversion	Cytoplasmic		1619	1619
group_1324	Function unknown	Cytoplasmic		362	362
group_207	Signal transduction mechanisms	InnerMembrane		338	1667
Mlc protein	Carbohydrate transport and metabolism	Cytoplasmic		1211	1211
group_1323	Signal transduction mechanisms	InnerMembrane		1583	1583
Putative transport protein AHA_2450	General function prediction only	InnerMembrane		1685	1721
uvrB	Replication, recombination and repair	Cytoplasmic		2012	2012
DedA family protein DedA	Cell wall/membrane/envelope biogenesis	InnerMembrane		653	653
nhaB	Energy production and conversion	InnerMembrane		1580	1580
fadR	Transcription	Cytoplasmic		713	713
YcgB family protein YcgB	Cell cycle control, cell division, chromosome partitioning	Cytoplasmic		1517	1517
UPF0229 protein WL1483_230	General function prediction only	Cytoplasmic		1271	1271
AAA_PrkA domain-containing protein	Signal transduction mechanisms	Cytoplasmic		1922	1922
sstT	Amino acid transport and metabolism	InnerMembrane		1250	1250
Serine transporter family protein	Amino acid transport and metabolism	InnerMembrane		1298	1298

MutT/nudix family protein	Defense mechanisms	Cytoplasmic		566	566
Fumarate hydratase class I	Energy production and conversion	Cytoplasmic		332	1523
group_1604	Posttranslational modification, protein turnover, chaperones	Periplasmic		428	440
group_1603	Signal transduction mechanisms	InnerMembrane		2801	2801
group_1602	Defense mechanisms	InnerMembrane		1736	1838
ABC-type multidrug transporter ATP-binding protein	Defense mechanisms	InnerMembrane		1751	1751
DUF4442 domain-containing protein	Unknown protein	InnerMembrane		473	473
S4 RNA-binding domain-containing protein	Translation, ribosomal structure and biogenesis	Cytoplasmic		233	233
Oligoendopeptidase F	Amino acid transport and metabolism	Cytoplasmic		1808	1808
DUF808 domain-containing protein	Replication, recombination and repair	InnerMembrane		917	917
group_5919	Intracellular trafficking, secretion, and vesicular transport	InnerMembrane		914	914
group_6600	Intracellular trafficking, secretion, and vesicular transport	InnerMembrane		1802	1802
Family of unknown function (DUF6279)	Unknown protein	Periplasmic		857	857
Penicillin-binding protein activator LpoB	Cell wall/membrane/envelope biogenesis	Periplasmic		596	596
Histidine triad nucleotide-binding protein	Nucleotide transport and metabolism	Cytoplasmic		350	350
group_6737	Signal transduction mechanisms	InnerMembrane		2012	2012
6-carboxy-5	Unknown protein	Cytoplasmic		884	884
pyrC	Nucleotide transport and metabolism	Cytoplasmic		1343	1343
bsr	Cell wall/membrane/envelope biogenesis	Cytoplasmic		1226	1226
Transport protein Alx	Inorganic ion transport and metabolism	InnerMembrane		944	944
oppD	Amino acid transport and metabolism	Cytoplasmic		980	986
Oligopeptide ABC transporter permease OppC	Amino acid transport and metabolism	InnerMembrane		911	911
oppB	Amino acid transport and metabolism	InnerMembrane		920	920
SBP_bac_5 domain-containing protein	Amino acid transport and metabolism	Periplasmic		1619	1619
group_5883	Amino acid transport and metabolism	InnerMembrane		635	635
Aldehyde-alcohol dehydrogenase	Energy production and conversion	Cytoplasmic		2663	2663
DUF3391 domain protein / HD-GYP family diguanylate phosphodiesterase	Signal transduction mechanisms	Cytoplasmic		1235	1235
group_1208	Signal transduction mechanisms	InnerMembrane		1670	1670
CstA protein	Energy production and conversion	InnerMembrane		1421	1421
group_192	Transcription	Cytoplasmic		1760	1805
dacB	Cell wall/membrane/envelope biogenesis	Periplasmic		1436	1436
grxA	Posttranslational modification, protein turnover, chaperones	Cytoplasmic		263	263
group_1205	Amino acid transport and metabolism	Cytoplasmic		1214	1274
group_6739	General function prediction only	InnerMembrane		1475	1475
fabV	Lipid transport and metabolism	Cytoplasmic		1193	1193
rnfA	Energy production and conversion	InnerMembrane		581	581

rnfB	Energy production and conversion	Periplasmic		563	563
Ion-translocating oxidoreductase complex subunit C	Energy production and conversion	Periplasmic		2576	2678
rnfD	Energy production and conversion	InnerMembrane		1052	1052
nth	Replication, recombination and repair	Cytoplasmic		641	641
gloA	Secondary metabolites biosynthesis, transport and catabolism	Cytoplasmic		413	413
Tyrosine-specific transport protein	Amino acid transport and metabolism	InnerMembrane		1193	1202
aroC	Amino acid transport and metabolism	Cytoplasmic		1088	1088
prmB	Translation, ribosomal structure and biogenesis	Cytoplasmic		935	935
UPF0115 protein AHA_2357	Replication, recombination and repair	Cytoplasmic		524	524
Sensory histidine kinase	Signal transduction mechanisms	Cytoplasmic		1295	1295
CENP-V/GFA domain-containing protein	Function unknown	Cytoplasmic		398	398
Chitin-binding type-3 domain-containing protein	Carbohydrate transport and metabolism	Periplasmic		1448	1448
Methyltranfer_dom domain-containing protein	Unknown protein	Cytoplasmic		1229	1229
PhnA_Zn_Ribbon domain-containing protein	General function prediction only	Periplasmic		578	578
group_826	Unknown protein	Cytoplasmic		203	203
mukB	Cell cycle control, cell division, chromosome partitioning	Cytoplasmic		4427	4427
Chromosome partition protein MukE	Cell cycle control, cell division, chromosome partitioning	Cytoplasmic		719	737
Chromosome partition protein MukF	Cell cycle control, cell division, chromosome partitioning	Cytoplasmic		1319	1382
tRNA 5-carboxymethoxyuridine methyltransferase	Coenzyme transport and metabolism	Cytoplasmic		776	776
tpiA	Carbohydrate transport and metabolism	Cytoplasmic		755	755
pfkA	Carbohydrate transport and metabolism	Cytoplasmic		971	971
M20D family peptidase	General function prediction only	Cytoplasmic		1256	1256
Predicted ATP-dependent endonuclease of the OLD family	Replication, recombination and repair	Cytoplasmic		1637	1637
HTH araC/xylS-type domain-containing protein	Transcription	Cytoplasmic		887	887
Probable membrane transporter protein	Inorganic ion transport and metabolism	InnerMembrane		779	779
tRNA-uridine aminocarboxypropyltransferase	Translation, ribosomal structure and biogenesis	Periplasmic		761	761
ddl	Cell wall/membrane/envelope biogenesis	Cytoplasmic		989	989
Fe2OG dioxygenase domain-containing protein	Translation, ribosomal structure and biogenesis	Cytoplasmic		608	608
Putative ATP-dependent RNA helicase RhlE	Replication, recombination and repair	InnerMembrane		1379	1385
DEP domain-containing protein	Unknown protein	Cytoplasmic		845	845
Nudix hydrolase domain-containing protein	Defense mechanisms	Cytoplasmic		392	392
GNAT family N-acetyltransferase	Translation, ribosomal structure and biogenesis	Cytoplasmic		506	506
Fe-ADH domain-containing protein	Energy production and conversion	Cytoplasmic		1118	1118
Zn-ribbon-containing protein	General function prediction only	Cytoplasmic		785	785
syd	Unknown protein	Cytoplasmic		548	548
queF	Translation, ribosomal structure and biogenesis	Cytoplasmic		848	848

group_1460	Cell wall/membrane/envelope biogenesis	InnerMembrane		2270	2270
GGDEF domain-containing protein	Signal transduction mechanisms	Cytoplasmic		1607	1619
AMP nucleosidase	Nucleotide transport and metabolism	Cytoplasmic		1355	1355
xni	Replication, recombination and repair	Cytoplasmic		803	803
isocitrate dehydrogenase	Energy production and conversion	Cytoplasmic		1007	1007
DNA-binding transcriptional activator GcvA	Transcription	Cytoplasmic		920	920
group_220	Signal transduction mechanisms	InnerMembrane		389	2141
dinB	Replication, recombination and repair	Cytoplasmic		1052	1052
(Na+)-NQR maturation NqrM	Energy production and conversion	Cytoplasmic		224	224
FAD:protein FMN transferase	Coenzyme transport and metabolism	Cytoplasmic		1040	1040
nqrF	Energy production and conversion	Cytoplasmic		1223	1223
group_5908	Energy production and conversion	InnerMembrane		596	596
nqrD	Energy production and conversion	InnerMembrane		632	632
nqrB	Energy production and conversion	InnerMembrane		1229	1229
nqrA	Energy production and conversion	Cytoplasmic		1343	1343
BolA family transcriptional regulator	Transcription	Cytoplasmic		308	314
group_1468	Function unknown	Periplasmic		575	575
group_5874	Posttranslational modification, protein turnover, chaperones	Periplasmic		551	551
group_1469	Unknown protein	Cytoplasmic		329	329
AmpG protein	Unknown protein	InnerMembrane		1379	1379
UPF0234 protein AHA_1129	Signal transduction mechanisms	Cytoplasmic		482	482
AHA_1846	Intracellular trafficking, secretion, and vesicular transport	Cytoplasmic		1430	1430
vasK/icmF	Intracellular trafficking, secretion, and vesicular transport	InnerMembrane		3485	3485
AHA_1844	Intracellular trafficking, secretion, and vesicular transport	Cytoplasmic		1439	1445
vasH/clpV	Signal transduction mechanisms	Cytoplasmic		1133	1538
hrpB	Translation, ribosomal structure and biogenesis	InnerMembrane		2447	2447
sfsA	Carbohydrate transport and metabolism	Cytoplasmic		743	743
dksA	Transcription	Cytoplasmic		449	449
pcnB	Translation, ribosomal structure and biogenesis	Cytoplasmic		1499	1499
panB	Coenzyme transport and metabolism	Cytoplasmic		794	794
panC	Coenzyme transport and metabolism	Cytoplasmic		857	857
group_1108	Inorganic ion transport and metabolism	Periplasmic		524	524
DUF3343 domain-containing protein	Unknown protein	Cytoplasmic		260	260
BcrAD_BadFG domain-containing protein	Lipid transport and metabolism	Cytoplasmic		782	782
2-hydroxyglutaryl-CoA dehydratase	Amino acid transport and metabolism	Cytoplasmic		1151	1151
group_5736	Defense mechanisms	InnerMembrane		773	773

group_6752	Defense mechanisms	Cytoplasmic		917	917
hpt	Nucleotide transport and metabolism	Cytoplasmic		530	530
LitR	Transcription	Cytoplasmic		614	614
Flavin prenyltransferase UbiX	Coenzyme transport and metabolism	Cytoplasmic		611	611
mpl	Cell wall/membrane/envelope biogenesis	Cytoplasmic		1370	1370
Inorganic pyrophosphatase	Energy production and conversion	Cytoplasmic		530	530
group_1104	Unknown protein	Periplasmic		878	878
YtfJ family protein	General function prediction only	Periplasmic		542	542
group_180	Cell wall/membrane/envelope biogenesis	Cytoplasmic		290	689
ABC-type antimicrobial peptide transport system	Defense mechanisms	InnerMembrane		1247	1247
DUF3299 domain-containing protein	Function unknown	Periplasmic		476	476
TIGR03899 family protein	Unknown protein	Cytoplasmic		866	866
Divalent anion:sodium symporter family protein	Carbohydrate transport and metabolism	InnerMembrane		1724	1724
cysN	Inorganic ion transport and metabolism	Cytoplasmic		1427	1442
cysD	Amino acid transport and metabolism	Cytoplasmic		908	908
Membrane protease	Posttranslational modification, protein turnover, chaperones	Cytoplasmic		920	920
NfeD domain-containing protein	Posttranslational modification, protein turnover, chaperones	InnerMembrane		455	455
CBS domain-containing protein	Signal transduction mechanisms	Cytoplasmic		410	413
ABC transporter ATPase	General function prediction only	Cytoplasmic		1910	1910
Ribosomal RNA large subunit methyltransferase J	Translation, ribosomal structure and biogenesis	Cytoplasmic		854	854
DUF2061 domain-containing protein	Function unknown	InnerMembrane		362	362
group_1094	Secondary metabolites biosynthesis, transport and catabolism	Cytoplasmic		371	428
putP	Amino acid transport and metabolism	InnerMembrane		1517	1517
group_6034	Cell wall/membrane/envelope biogenesis	Periplasmic		926	926
Miniconductance mechanosensitive channel	Cell wall/membrane/envelope biogenesis	InnerMembrane		1241	1241
Acetate kinase	Energy production and conversion	Cytoplasmic		1199	1199
PlsC domain-containing protein	Lipid transport and metabolism	Cytoplasmic		1106	1106
Aspartate racemase	Cell wall/membrane/envelope biogenesis	Cytoplasmic		692	692
group_6260	Amino acid transport and metabolism	InnerMembrane		590	590
group_1091	Transcription	Cytoplasmic		1376	1376
group_1090	Unknown protein	Periplasmic		464	464
Putative exported protein	Unknown protein	Periplasmic		2060	2066
group_1088	General function prediction only	InnerMembrane		1313	1313
group_6455	Secondary metabolites biosynthesis, transport and catabolism	Cytoplasmic		839	839
group_1087	Unknown protein	Cytoplasmic		563	563
Phosphatase YbhA	Coenzyme transport and metabolism	Cytoplasmic		821	821

group_1085	Carbohydrate transport and metabolism	InnerMembrane		1241	1241
AsnC family transcriptional regulator	Transcription	Cytoplasmic		476	476
group_5872	Unknown protein	Periplasmic		317	317
DNA-binding MarR family transcriptional regulator	Transcription	Cytoplasmic		425	425
PKS_ER domain-containing protein	Secondary metabolites biosynthesis, transport and catabolism	Cytoplasmic		1004	1004
group_1082	Nucleotide transport and metabolism	Cytoplasmic		443	443
glgX	Carbohydrate transport and metabolism	Cytoplasmic		2141	2141
1	Carbohydrate transport and metabolism	Periplasmic		2168	2177
malQ	Carbohydrate transport and metabolism	Cytoplasmic		1082	2189
gap	Carbohydrate transport and metabolism	Cytoplasmic		995	995
nadA	Coenzyme transport and metabolism	Cytoplasmic		1073	1073
group_810	Signal transduction mechanisms	InnerMembrane		1841	2105
MATE family efflux transporter	Defense mechanisms	InnerMembrane		1346	1346
group_417	Unknown protein	Periplasmic		590	590
DUF465 domain-containing protein	Function unknown	Cytoplasmic		215	215
nifJ	Energy production and conversion	Cytoplasmic		3575	3575
Pyridine nucleotide-disulphide oxidoreductase family protein	Amino acid transport and metabolism	Cytoplasmic		1646	1646
Methyl-accepting transducer domain-containing protein	Signal transduction mechanisms	InnerMembrane		1712	1712
htpX	Posttranslational modification, protein turnover, chaperones	InnerMembrane		872	872
bioD	Coenzyme transport and metabolism	Cytoplasmic		680	680
bioB	Coenzyme transport and metabolism	Cytoplasmic		1088	1088
bioA	Coenzyme transport and metabolism	Cytoplasmic		1274	1274
asnS	Translation, ribosomal structure and biogenesis	Cytoplasmic		1400	1400
group_411	Carbohydrate transport and metabolism	InnerMembrane		1343	1343
Anaerobic C4-dicarboxylate transporter	Energy production and conversion	InnerMembrane		1376	1376
group_6527	Unknown protein	Cytoplasmic		314	314
group_5862	Transcription	Cytoplasmic		911	911
YffB family protein YffB	Inorganic ion transport and metabolism	Cytoplasmic		350	350
dapE	Amino acid transport and metabolism	Cytoplasmic		1127	1127
VanY domain-containing protein	Cell wall/membrane/envelope biogenesis	Cytoplasmic		689	689
group_5840	Unknown protein	Cytoplasmic		146	146
nhaP2	Energy production and conversion	InnerMembrane		1727	1727
Hyaluronidase	Unknown protein	Periplasmic		1103	1103
Amino acid transporter LysE	Amino acid transport and metabolism	InnerMembrane		632	632
DUF1523 domain-containing protein	Unknown protein	Periplasmic		530	530
Winged helix-turn helix	Function unknown	Cytoplasmic		296	296

RihA product	Nucleotide transport and metabolism	Cytoplasmic		935	935
udp	Nucleotide transport and metabolism	Cytoplasmic		755	755
fucO	Energy production and conversion	Cytoplasmic		1151	1151
group_6324	General function prediction only	Cytoplasmic		257	257
cysM	Amino acid transport and metabolism	Cytoplasmic		893	893
group_420	Energy production and conversion	Cytoplasmic		1184	1184
group_1616	Lipid transport and metabolism	Cytoplasmic		1421	1430
group_5664	Unknown protein	Cytoplasmic		293	293
group_6128	Transcription	Cytoplasmic		908	908
Alkyl hydroperoxide reductase C	Defense mechanisms	Cytoplasmic		602	602
ATPgrasp_ST domain-containing protein	Lipid transport and metabolism	Cytoplasmic		965	965
Gonadoliberin III	Unknown protein	InnerMembrane		1538	1538
Zn_protease domain-containing protein	Function unknown	Periplasmic		788	788
cmoB	Secondary metabolites biosynthesis, transport and catabolism	Cytoplasmic		977	977
aspS	Translation, ribosomal structure and biogenesis	Cytoplasmic		1766	1766
Probable transcriptional regulatory protein AHA_1522	Transcription	Cytoplasmic		740	740
mcr-3	Cell wall/membrane/envelope biogenesis	InnerMembrane		1616	1616
N-acetylglucosamine repressor	Carbohydrate transport and metabolism	Cytoplasmic		1214	1214
nagA	Carbohydrate transport and metabolism	Cytoplasmic		1145	1145
nagB	Carbohydrate transport and metabolism	Cytoplasmic		800	800
Beta-N-acetylhexosaminidase	Carbohydrate transport and metabolism	Periplasmic		2663	2663
glnS	Translation, ribosomal structure and biogenesis	Cytoplasmic		1661	1661
fur	Inorganic ion transport and metabolism	Cytoplasmic		428	428
Translation elongation factor P (EF-P)	Unknown protein	Periplasmic		482	482
Flavodoxin	Energy production and conversion	Cytoplasmic		524	524
LexA family transcriptional regulator	Unknown protein	Cytoplasmic		296	296
DUF2788 domain-containing protein	Unknown protein	Cytoplasmic		221	221
pgm	Carbohydrate transport and metabolism	Periplasmic		1649	1649
Conserved integral membrane protein	Carbohydrate transport and metabolism	InnerMembrane		881	881
group_6552	Transcription	Cytoplasmic		482	482
Phosphatidylglycerophosphate synthase	Lipid transport and metabolism	InnerMembrane		602	602
lpdA	Energy production and conversion	InnerMembrane		2150	2153
4-carboxymuconolactone decarboxylase domain protein	Inorganic ion transport and metabolism	Cytoplasmic		581	581
Peptide-methionine (S)-S-oxide reductase	Unknown protein	Periplasmic		560	560
PpiC domain-containing protein	Posttranslational modification, protein turnover, chaperones	Cytoplasmic		278	278
group_433	Unknown protein	InnerMembrane		374	374

translocation/assembly module TamB	Intracellular trafficking, secretion, and vesicular transport	Periplasmic		3752	3767
glpX	Carbohydrate transport and metabolism	Cytoplasmic		1010	1010
Proteinase inhibitor	General function prediction only	Cytoplasmic		251	251
hemY	Function unknown	InnerMembrane		1163	1163
Heme biosynthesis operon protein HemX	General function prediction only	Periplasmic		1073	1076
hemC	Coenzyme transport and metabolism	Cytoplasmic		929	992
cya	Signal transduction mechanisms	Cytoplasmic		2534	2534
Abhydrolase_2 domain-containing protein	General function prediction only	Cytoplasmic		653	671
lysA	Amino acid transport and metabolism	Cytoplasmic		1250	1250
dapF	Amino acid transport and metabolism	Cytoplasmic		830	830
DUF484 family protein	Function unknown	Cytoplasmic		680	680
group_602	Cell wall/membrane/envelope biogenesis	InnerMembrane		1526	1526
group_600	Amino acid transport and metabolism	Cytoplasmic		770	770
Haloacid dehalogenase	Coenzyme transport and metabolism	Cytoplasmic		809	809
group_598	Defense mechanisms	Cytoplasmic		929	929
Transport permease protein	Defense mechanisms	InnerMembrane		1094	1094
HD-GYP domain-containing protein	Signal transduction mechanisms	Cytoplasmic		1238	1238
metAS	Amino acid transport and metabolism	Cytoplasmic		947	953
ArsR family transcriptional regulator	Transcription	Cytoplasmic		470	470
Phosphoglycerol transferase I	Cell wall/membrane/envelope biogenesis	InnerMembrane		1964	1964
DUF2442 domain-containing protein	Unknown protein	Cytoplasmic		257	257
Aamy domain-containing protein	Carbohydrate transport and metabolism	Periplasmic		1616	1619
group_591	Inorganic ion transport and metabolism	InnerMembrane		533	533
ATP-grasp domain-containing protein	Amino acid transport and metabolism	Cytoplasmic		1442	1442
group_590	Translation, ribosomal structure and biogenesis	Cytoplasmic		458	479
3-hydroxy acid dehydrogenase	Energy production and conversion	Cytoplasmic		746	746
Dipeptidase	Amino acid transport and metabolism	Periplasmic		1472	1673
Isochorismatase	Secondary metabolites biosynthesis, transport and catabolism	Cytoplasmic		923	926
entE	Secondary metabolites biosynthesis, transport and catabolism	Cytoplasmic		1007	1667
group_564	Unknown protein	Periplasmic		551	551
group_565	General function prediction only	Periplasmic		2000	2000
glgC	Carbohydrate transport and metabolism	Cytoplasmic		1274	1274
group_566	Unknown protein	Cytoplasmic		494	494
group_5939	Unknown protein	Cytoplasmic		362	362
Inosine/guanosine kinase	Carbohydrate transport and metabolism	Cytoplasmic		1304	1304
hemH	Coenzyme transport and metabolism	Cytoplasmic		974	974

adk	Nucleotide transport and metabolism	Cytoplasmic		644	644
htpG	Posttranslational modification, protein turnover, chaperones	Cytoplasmic		1913	1946
recR	Replication, recombination and repair	Cytoplasmic		602	602
Sulfur carrier protein FdhD	Energy production and conversion	Cytoplasmic		857	857
group_568	Unknown protein	InnerMembrane		398	398
group_6196	General function prediction only	Periplasmic		1676	1676
Formate dehydrogenase	General function prediction only	Cytoplasmic		419	419
Electron transport protein HydN	Energy production and conversion	Cytoplasmic		599	599
kup2	Inorganic ion transport and metabolism	InnerMembrane		1868	1868
Hydrogenase-4 component A	Energy production and conversion	Periplasmic		665	665
Hydrogenase-4 component B	Energy production and conversion	InnerMembrane		1883	1883
hycD	Energy production and conversion	InnerMembrane		953	953
hycE	Energy production and conversion	Cytoplasmic		1718	1718
Hydrogenase-4 component H	Energy production and conversion	Periplasmic		566	566
Hydrogenase	Energy production and conversion	Cytoplasmic		815	815
hycI	Energy production and conversion	Cytoplasmic		509	509
group_574	Translation, ribosomal structure and biogenesis	Cytoplasmic		1883	1883
hypC	Posttranslational modification, protein turnover, chaperones	Cytoplasmic		251	251
hypD	Posttranslational modification, protein turnover, chaperones	Cytoplasmic		1118	1118
hypE	Posttranslational modification, protein turnover, chaperones	Cytoplasmic		1004	1004
hypA	Posttranslational modification, protein turnover, chaperones	Cytoplasmic		341	341
Sugar phosphate permease	Carbohydrate transport and metabolism	InnerMembrane		1337	1337
Cof-type HAD-IIB family hydrolase	Coenzyme transport and metabolism	Cytoplasmic		812	812
Lysophospholipase L2	Lipid transport and metabolism	Cytoplasmic		1040	1040
tRNA (guanosine(18)-2'-O)-methyltransferase	Translation, ribosomal structure and biogenesis	Cytoplasmic		707	749
Bifunctional (P)ppGpp synthetase II and guanosine-3'	Signal transduction mechanisms	Cytoplasmic		2117	2117
rpoZ	Transcription	Cytoplasmic		275	275
gmk	Nucleotide transport and metabolism	Cytoplasmic		626	854
3-deoxy-D-manno-octulosonic acid kinase	Signal transduction mechanisms	Cytoplasmic		713	713
Probable biotin transporter	Carbohydrate transport and metabolism	InnerMembrane		854	854
group_6574	Unknown protein	Cytoplasmic		161	161
Integral membrane protein	Carbohydrate transport and metabolism	InnerMembrane		899	899
group_6674	Transcription	Cytoplasmic		974	974
group_6367	Translation, ribosomal structure and biogenesis	Cytoplasmic		1013	1013
Universal stress protein	Signal transduction mechanisms	Cytoplasmic		434	434
Ferritin	Inorganic ion transport and metabolism	Cytoplasmic		524	524

Putative oxidoreductase with FAD/NAD(P)-binding domain	General function prediction only	Cytoplasmic		1184	1184
group_893	Inorganic ion transport and metabolism	InnerMembrane		1220	1220
Leucine efflux protein LeuE	Amino acid transport and metabolism	InnerMembrane		638	638
group_6213	Signal transduction mechanisms	InnerMembrane		1658	1673
LytTR family two component transcriptional regulator	Transcription	Cytoplasmic		731	731
MFS domain-containing protein	Carbohydrate transport and metabolism	InnerMembrane		1190	1190
purK	Nucleotide transport and metabolism	Cytoplasmic		1124	1124
group_6477	Nucleotide transport and metabolism	Cytoplasmic		509	509
group_5726	Unknown protein	Cytoplasmic		302	302
Sodium bicarbonate cotransporter	Function unknown	InnerMembrane		926	926
group_6735	Transcription	Cytoplasmic		902	902
RRM domain-containing protein	Translation, ribosomal structure and biogenesis	Periplasmic		491	494
group_435	Unknown protein	InnerMembrane		449	449
Iron permease FTR1 family	Inorganic ion transport and metabolism	InnerMembrane		818	818
Amino_oxidase domain-containing protein	Coenzyme transport and metabolism	Cytoplasmic		1424	1424
group_438	Unknown protein	Periplasmic		560	560
Bcr/CflA family efflux transporter	Carbohydrate transport and metabolism	InnerMembrane		1109	1190
DUF4357 domain-containing protein	Unknown protein	Periplasmic		467	467
group_439	Cell wall/membrane/envelope biogenesis	Cytoplasmic		680	680
Cell division protein FtsX	Cell wall/membrane/envelope biogenesis	InnerMembrane		2435	2447
L-lactate permease	Energy production and conversion	InnerMembrane		1697	1697
LysR family transcriptional regulator	Transcription	Cytoplasmic		905	905
group_6294	Coenzyme transport and metabolism	Cytoplasmic		782	782
DUF3802 domain-containing protein	Unknown protein	Cytoplasmic		335	335
argA	Amino acid transport and metabolism	Cytoplasmic		1328	1328
Aspartokinase	Amino acid transport and metabolism	Cytoplasmic		1352	1352
group_447	Unknown protein	Cytoplasmic		1088	1088
Sulfatase domain-containing protein	Cell wall/membrane/envelope biogenesis	InnerMembrane		2261	2261
UPF0145 protein AHA_2580	Function unknown	Cytoplasmic		317	317
NADH dehydrogenase	Energy production and conversion	Cytoplasmic		1292	1292
UPF0227 protein C0073_012720	General function prediction only	Cytoplasmic		539	539
group_6368	Unknown protein	Cytoplasmic		452	455
flavohemoglobin expression-modulating QEGLA motif protein	Function unknown	Cytoplasmic		1337	1337
Thymidine kinase	Nucleotide transport and metabolism	Cytoplasmic		581	581
tet(35)	Energy production and conversion	InnerMembrane		1580	1580
Corrinoid adenosyltransferase	Coenzyme transport and metabolism	Cytoplasmic		563	563

DUF3820 family protein	Function unknown	Cytoplasmic		230	230
7-carboxy-7-deazaguanine synthase	Coenzyme transport and metabolism	Cytoplasmic		671	671
queC	Translation, ribosomal structure and biogenesis	Cytoplasmic		707	707
rrtA	Unknown protein	InnerMembrane		524	524
pyrF	Nucleotide transport and metabolism	Cytoplasmic		701	701
Probable lipopolysaccharide assembly protein A	Function unknown	InnerMembrane		284	284
ihfB	Replication, recombination and repair	Cytoplasmic		284	284
rpsA	Translation, ribosomal structure and biogenesis	Cytoplasmic		1670	1670
cmk	Nucleotide transport and metabolism	Cytoplasmic		692	692
ribA	Coenzyme transport and metabolism	Cytoplasmic		593	593
cysB	Transcription	Cytoplasmic		974	974
group_6253	Transcription	Cytoplasmic		875	875
group_6557	Carbohydrate transport and metabolism	InnerMembrane		1208	1208
DNA repair protein	Unknown protein	Cytoplasmic		764	764
group_520	Signal transduction mechanisms	Cytoplasmic		1970	1970
group_519	Transcription	Cytoplasmic		938	938
group_6417	Signal transduction mechanisms	Cytoplasmic		431	431
group_518	General function prediction only	Cytoplasmic		899	899
RNA-binding protein	Translation, ribosomal structure and biogenesis	Cytoplasmic		278	278
Penicillin-insensitive murein endopeptidase	Cell wall/membrane/envelope biogenesis	Periplasmic		764	764
group_6035	Function unknown	InnerMembrane		731	734
TPM_phosphatase domain-containing protein	Function unknown	InnerMembrane		617	617
group_6755	Cell motility	InnerMembrane		758	758
group_514	Cell motility	Periplasmic		875	875
nuoA	Energy production and conversion	InnerMembrane		404	404
nuoB	Energy production and conversion	Cytoplasmic		674	674
nuoC	Energy production and conversion	Cytoplasmic		1805	1805
nuoE	Energy production and conversion	Cytoplasmic		542	542
nuoF	Energy production and conversion	Cytoplasmic		671	1367
nuoG	Energy production and conversion	Cytoplasmic		2729	2729
nuoH	Energy production and conversion	InnerMembrane		968	968
nuoI	Energy production and conversion	Cytoplasmic		542	542
NADH-quinone oxidoreductase subunit J	Energy production and conversion	InnerMembrane		455	548
nuoK	Energy production and conversion	InnerMembrane		308	308
NADH-quinone oxidoreductase chain l	Energy production and conversion	InnerMembrane		1847	1847
NADH dehydrogenase I subunit M	Energy production and conversion	InnerMembrane		1520	1520

nuoN	Energy production and conversion	InnerMembrane		1463	1463
ABC transporter substrate binding protein	General function prediction only	InnerMembrane		908	911
PepSY-associated TM helix family	Function unknown	InnerMembrane		620	620
Putative monooxygenase YdhR	Unknown protein	Periplasmic		305	305
OMP_b-brl domain-containing protein	Cell wall/membrane/envelope biogenesis	Periplasmic		602	620
Protein ExoD	Cell wall/membrane/envelope biogenesis	InnerMembrane		638	638
Serine/threonine protein kinase	Amino acid transport and metabolism	InnerMembrane		1274	1280
group_6757	Unknown protein	Cytoplasmic		317	317
group_614	Translation, ribosomal structure and biogenesis	Cytoplasmic		413	416
thiG	Coenzyme transport and metabolism	Cytoplasmic		767	767
thiS	Coenzyme transport and metabolism	Cytoplasmic		206	206
Phosphomethylpyrimidine synthase	Coenzyme transport and metabolism	Cytoplasmic		1991	1991
group_6562	Unknown protein	Cytoplasmic		479	479
DUF5666 domain-containing protein	Unknown protein	Cytoplasmic		416	416
group_6097	Signal transduction mechanisms	Cytoplasmic		668	668
group_620	Signal transduction mechanisms	InnerMembrane		1211	1235
rimK	Amino acid transport and metabolism	Cytoplasmic		896	896
Na_H_Exchanger domain-containing protein	Inorganic ion transport and metabolism	InnerMembrane		1277	1277
lolE	Cell wall/membrane/envelope biogenesis	InnerMembrane		1241	1241
lolD	Cell wall/membrane/envelope biogenesis	Cytoplasmic		698	698
Lipoprotein-releasing system transmembrane protein LolC	Cell wall/membrane/envelope biogenesis	InnerMembrane		1235	1235
PilZ domain-containing protein	Unknown protein	Cytoplasmic		575	575
mfd	Replication, recombination and repair	Cytoplasmic		3377	3455
group_6698	Carbohydrate transport and metabolism	InnerMembrane		917	917
DUF1107 domain-containing protein	Unknown protein	Cytoplasmic		206	206
sixA	Signal transduction mechanisms	Cytoplasmic		467	467
Pitrilysin	Posttranslational modification, protein turnover, chaperones	Cytoplasmic		2774	2774
tmpT	Unknown protein	Cytoplasmic		653	653
Glucose 1-dehydrogenase 2	Lipid transport and metabolism	InnerMembrane		764	764
Methylated-DNA--[protein]-cysteine S-methyltransferase	Replication, recombination and repair	Cytoplasmic		827	827
group_5833	Unknown protein	Periplasmic		272	272
guaA	Nucleotide transport and metabolism	Cytoplasmic		1589	1589
guaB	Nucleotide transport and metabolism	Cytoplasmic		1463	1463
kup1	Inorganic ion transport and metabolism	InnerMembrane		1871	1871
MotA_ExbB domain-containing protein	Intracellular trafficking, secretion, and vesicular transport	InnerMembrane		755	755
group_6395	Carbohydrate transport and metabolism	InnerMembrane		929	929

Exodeoxyribonuclease 7 large subunit	Replication, recombination and repair	Cytoplasmic		686	1364
Elp3 domain-containing protein	Coenzyme transport and metabolism	Cytoplasmic		1370	1370
Inner membrane transport protein YhaO	Amino acid transport and metabolism	InnerMembrane		1295	1295
L-PSP (mRNA) endoribonuclease	Defense mechanisms	Cytoplasmic		383	383
UPF0597 protein AHA_1619	Amino acid transport and metabolism	InnerMembrane		1289	1289
PAS domain-containing protein	Transcription	Cytoplasmic		653	653
Fructose-26-bisphosphatase	Carbohydrate transport and metabolism	Cytoplasmic		587	587
DUF3427 domain-containing protein	Transcription	Cytoplasmic		3143	3143
group_5861	Function unknown	Periplasmic		467	467
Glyoxalase domain-containing protein	Secondary metabolites biosynthesis, transport and catabolism	Cytoplasmic		368	368
Hydrolase_4 domain-containing protein	Lipid transport and metabolism	InnerMembrane		1583	1583
CMP/dCMP-type deaminase domain-containing protein	Translation, ribosomal structure and biogenesis	Periplasmic		503	503
group_6717	Transcription	Cytoplasmic		848	848
group_1075	Carbohydrate transport and metabolism	InnerMembrane		1199	1202
DUF2496 domain-containing protein	Unknown protein	Cytoplasmic		155	155
Inner membrane protein YebE	Function unknown	Periplasmic		650	650
group_1078	Carbohydrate transport and metabolism	InnerMembrane		1172	1181
2	Secondary metabolites biosynthesis, transport and catabolism	Cytoplasmic		800	803
group_1079	Transcription	Cytoplasmic		902	902
glpK	Energy production and conversion	Cytoplasmic		1502	1502
Aquaporin	Carbohydrate transport and metabolism	InnerMembrane		848	848
glpT	Carbohydrate transport and metabolism	InnerMembrane		1355	1355
DNA-binding transcriptional repressor	Transcription	Cytoplasmic		758	758
glpD	Energy production and conversion	Cytoplasmic		1487	1487
pdxJ	Coenzyme transport and metabolism	Cytoplasmic		737	737
recO	Replication, recombination and repair	Cytoplasmic		707	710
era	Translation, ribosomal structure and biogenesis	Cytoplasmic		899	899
rnc	Transcription	Cytoplasmic		671	671
lepB	Intracellular trafficking, secretion, and vesicular transport	InnerMembrane		923	923
lepA	Translation, ribosomal structure and biogenesis	Cytoplasmic		1793	1793
Sigma-E factor regulatory protein RseC	Signal transduction mechanisms	InnerMembrane		461	461
Sigma-E factor regulatory protein RseB	Signal transduction mechanisms	Cytoplasmic		884	980
DUF853 domain-containing protein	Replication, recombination and repair	Cytoplasmic		1508	1508
group_1551	Function unknown	InnerMembrane		494	494
DUF2062 domain-containing protein	Function unknown	InnerMembrane		518	518
group_1552	Intracellular trafficking, secretion, and vesicular transport	InnerMembrane		1529	2114

msbA	Defense mechanisms	InnerMembrane		1769	1769
lpxK	Cell wall/membrane/envelope biogenesis	InnerMembrane		1001	1001
UPF0434 protein AHA_2776	Translation, ribosomal structure and biogenesis	Cytoplasmic		188	188
group_6119	Inorganic ion transport and metabolism	InnerMembrane		788	788
arcD	Amino acid transport and metabolism	InnerMembrane		1436	1436
fabG	Lipid transport and metabolism	InnerMembrane		761	761
mmsB	Lipid transport and metabolism	Cytoplasmic		920	932
Crotonase	Lipid transport and metabolism	Cytoplasmic		1103	1103
3-hydroxybutyryl-CoA dehydratase	Lipid transport and metabolism	Cytoplasmic		797	812
Acyl-CoA dehydrogenase	Lipid transport and metabolism	Cytoplasmic		1157	1157
mmsA	Lipid transport and metabolism	Cytoplasmic		1286	1511
Isovaleryl-CoA dehydrogenase	Lipid transport and metabolism	Cytoplasmic		1148	1148
3-methylcrotonyl CoA carboxylase	Lipid transport and metabolism	Cytoplasmic		1601	1601
Enoyl-CoA hydratase/isomerase family protein	Lipid transport and metabolism	Cytoplasmic		839	839
Hydroxymethylglutaryl-CoA lyase	Amino acid transport and metabolism	Cytoplasmic		956	956
Transcriptional regulator	Transcription	Cytoplasmic		422	422
Acetate uptake transporter	Energy production and conversion	InnerMembrane		596	728
Glucose-regulated metallo-peptidase M90	Signal transduction mechanisms	Cytoplasmic		809	809
Sodium:alanine symporter family protein	Amino acid transport and metabolism	InnerMembrane		1499	1499
crotonase/enoyl-CoA hydratase family protein	Lipid transport and metabolism	Cytoplasmic		806	806
actP	Energy production and conversion	InnerMembrane		1655	1655
group_6729	General function prediction only	Cytoplasmic		1568	1592
Peptidylprolyl cis-trans isomerase D	Posttranslational modification, protein turnover, chaperones	Periplasmic		1913	1913
lon	Posttranslational modification, protein turnover, chaperones	Cytoplasmic		2354	2354
clpX	Posttranslational modification, protein turnover, chaperones	Cytoplasmic		1274	1274
clpP	Posttranslational modification, protein turnover, chaperones	Cytoplasmic		623	623
tig	Posttranslational modification, protein turnover, chaperones	Cytoplasmic		1310	1310
Inner membrane protein YccF	Function unknown	InnerMembrane		434	434
5'-deoxynucleotidase BTN33_16320	Nucleotide transport and metabolism	Cytoplasmic		590	590
pgi	Carbohydrate transport and metabolism	Cytoplasmic		1649	1649
Holin	Unknown protein	InnerMembrane		188	188
Mechanosensitive ion channel	Cell wall/membrane/envelope biogenesis	InnerMembrane		836	836
Nucleoid occlusion factor SlmA	Transcription	Cytoplasmic		596	596
dut	Nucleotide transport and metabolism	Cytoplasmic		458	458
coaBC	Coenzyme transport and metabolism	Cytoplasmic		1202	1202
UPF0758 protein AHA_0160	Replication, recombination and repair	Cytoplasmic		674	746

rpmB	Translation, ribosomal structure and biogenesis	Cytoplasmic		236	236
rpmG	Translation, ribosomal structure and biogenesis	Cytoplasmic		167	167
mutM	Replication, recombination and repair	Cytoplasmic		812	812
DUF535 family protein YbjX	Function unknown	Cytoplasmic		944	944
coaD	Coenzyme transport and metabolism	Cytoplasmic		482	482
ADP-heptose--LPS heptosyltransferase WaaF	Cell wall/membrane/envelope biogenesis	Periplasmic		1043	1043
Glycosyl transferase family protein	Cell wall/membrane/envelope biogenesis	Cytoplasmic		788	788
waaF	Cell wall/membrane/envelope biogenesis	Cytoplasmic		1109	1115
3-deoxy-D-manno-octulosonic acid transferase	Cell wall/membrane/envelope biogenesis	Cytoplasmic		1265	1265
Phosphotransferase enzyme II	Carbohydrate transport and metabolism	Cytoplasmic		287	287
cmtB	Carbohydrate transport and metabolism	Cytoplasmic		320	443
group_6255	Transcription	Cytoplasmic		1022	1022
group_1437	Energy production and conversion	Cytoplasmic		980	980
DUF2799 domain-containing protein	Unknown protein	Cytoplasmic		335	335
group_5724	Unknown protein	Cytoplasmic		170	170
Flagellar protein FliL	Cell motility	Cytoplasmic		434	434
ubiA	Coenzyme transport and metabolism	InnerMembrane		863	863
plsB	Lipid transport and metabolism	Cytoplasmic		2423	2423
lexA	Transcription	Cytoplasmic		623	623
SCO family protein	Posttranslational modification, protein turnover, chaperones	Periplasmic		626	626
trmL	Translation, ribosomal structure and biogenesis	Cytoplasmic		470	470
group_1431	Signal transduction mechanisms	InnerMembrane		1355	1355
group_6753	Signal transduction mechanisms	Cytoplasmic		695	695
fieF	Inorganic ion transport and metabolism	InnerMembrane		911	911
cysE	Amino acid transport and metabolism	Cytoplasmic		779	779
group_1430	Unknown protein	InnerMembrane		1280	1280
zapB	Cell cycle control, cell division, chromosome partitioning	Cytoplasmic		209	209
Glyoxalase	Defense mechanisms	Cytoplasmic		650	650
polA	Replication, recombination and repair	Cytoplasmic		2750	2753
rpoE	Transcription	Cytoplasmic		581	581
nadB	Coenzyme transport and metabolism	Cytoplasmic		1607	1607
FAD assembly factor SdhE	Posttranslational modification, protein turnover, chaperones	Cytoplasmic		269	269
ygfZ	Posttranslational modification, protein turnover, chaperones	Periplasmic		905	905
HTH lysR-type domain-containing protein	Transcription	Cytoplasmic		878	878
Chlorhexidine efflux transporter	Function unknown	InnerMembrane		437	437
group_1271	Unknown protein	Cytoplasmic		338	341

fbaA	Carbohydrate transport and metabolism	Cytoplasmic		1079	1079
pgk	Carbohydrate transport and metabolism	Cytoplasmic		1163	1163
epd	Carbohydrate transport and metabolism	Cytoplasmic		1010	1010
Sugar kinase	Carbohydrate transport and metabolism	Cytoplasmic		929	929
hemE	Coenzyme transport and metabolism	Cytoplasmic		1067	1067
Bifunctional protein PutA	Amino acid transport and metabolism	Cytoplasmic		3164	3164
SH3b domain-containing protein	General function prediction only	Periplasmic		605	605
group_6404	Signal transduction mechanisms	InnerMembrane		1250	1250
N-acetyltransferase domain-containing protein	Amino acid transport and metabolism	Cytoplasmic		440	440
adenylyltransferase/adenylyl-removing enzyme	Posttranslational modification, protein turnover, chaperones	Cytoplasmic		2873	2873
Restriction endonuclease	Function unknown	Cytoplasmic		1091	1091
Threonine synthase	Amino acid transport and metabolism	Cytoplasmic		1211	1211
amino acid ABC transporter permease	Amino acid transport and metabolism	InnerMembrane		665	665
ABC-type glutamine/glutamate transporter	Amino acid transport and metabolism	InnerMembrane		662	662
group_1263	Amino acid transport and metabolism	InnerMembrane		761	761
PBPb domain-containing protein	Amino acid transport and metabolism	Periplasmic		836	836
macB1	Cell wall/membrane/envelope biogenesis	InnerMembrane		1946	1949
DNA-binding response regulator	Signal transduction mechanisms	Cytoplasmic		680	680
group_1065	Signal transduction mechanisms	InnerMembrane		1655	1655
folE	Coenzyme transport and metabolism	Cytoplasmic		656	656
Molybdopterin molybdenumtransferase	Coenzyme transport and metabolism	Cytoplasmic		1226	1226
moeB	Coenzyme transport and metabolism	Cytoplasmic		755	755
AB hydrolase-1 domain-containing protein	Coenzyme transport and metabolism	Cytoplasmic		776	776
modC	Inorganic ion transport and metabolism	InnerMembrane		1076	1076
modB	Inorganic ion transport and metabolism	InnerMembrane		680	719
modA	Inorganic ion transport and metabolism	Periplasmic		728	728
MPT synthase subunit 2	Coenzyme transport and metabolism	Cytoplasmic		473	473
moaD	Coenzyme transport and metabolism	Cytoplasmic		245	245
Cytochrome c-type protein	Energy production and conversion	Periplasmic		590	590
Periplasmic nitrate reductase	Energy production and conversion	Periplasmic		455	455
napH	Energy production and conversion	InnerMembrane		875	875
Ferredoxin-type protein NapG	Energy production and conversion	Cytoplasmic		734	734
napA	Energy production and conversion	Periplasmic		2489	2489
moaA	Coenzyme transport and metabolism	Cytoplasmic		1028	1028
Sensor protein	Signal transduction mechanisms	Cytoplasmic		1712	1712
LuxR family transcriptional regulator 11	Signal transduction mechanisms	Cytoplasmic		638	638

ATP-dependent protease	Function unknown	Cytoplasmic		575	575
group_80	Signal transduction mechanisms	Cytoplasmic		632	1178
Putative gluconeogenesis factor	Carbohydrate transport and metabolism	Cytoplasmic		911	911
Class II glutamine amidotransferase	General function prediction only	Cytoplasmic		779	779
group_6670	Carbohydrate transport and metabolism	Cytoplasmic		578	578
fadE	Lipid transport and metabolism	InnerMembrane		2456	2456
Phosphodiesterase	Signal transduction mechanisms	Cytoplasmic		1517	1517
dnaQ	Replication, recombination and repair	Cytoplasmic		734	734
rnhA	Replication, recombination and repair	Cytoplasmic		464	464
Methyltransf_11 domain-containing protein	Coenzyme transport and metabolism	Cytoplasmic		722	722
Peptidoglycan lytic exotransglycosylase	Cell wall/membrane/envelope biogenesis	Periplasmic		1586	1586
Endo/exonuclease/phosphatase domain-containing protein	General function prediction only	Cytoplasmic		845	845
group_5807	Unknown protein	Cytoplasmic		224	224
Acyl-CoA thioester hydrolase YciA	Lipid transport and metabolism	Cytoplasmic		395	395
group_6517	Unknown protein	Cytoplasmic		188	188
Epimerase domain-containing protein	Cell wall/membrane/envelope biogenesis	Cytoplasmic		1013	1019
Membrane protein	Function unknown	InnerMembrane		626	626
ABC-type hemin transporter	Inorganic ion transport and metabolism	InnerMembrane		1031	1031
group_1166	Inorganic ion transport and metabolism	Periplasmic		839	848
hutX	Inorganic ion transport and metabolism	Cytoplasmic		512	512
hutZ	Inorganic ion transport and metabolism	Cytoplasmic		557	557
Lipoprotein	Unknown protein	Periplasmic		404	404
MFS_1_like domain-containing protein	Unknown protein	InnerMembrane		1139	1154
group_1162	Transcription	Cytoplasmic		209	209
group_6516	Amino acid transport and metabolism	InnerMembrane		3500	3500
Chitinase	Carbohydrate transport and metabolism	Periplasmic		2609	2609
group_1160	Carbohydrate transport and metabolism	Periplasmic		1922	1922
Catabolite activator protein	Signal transduction mechanisms	Cytoplasmic		638	638
Chain A	General function prediction only	Cytoplasmic		404	404
group_1158	Cell wall/membrane/envelope biogenesis	Periplasmic		296	296
DUF4136 domain-containing protein	Unknown protein	Periplasmic		530	533
Phosphoribulokinase	Carbohydrate transport and metabolism	Cytoplasmic		869	869
arnE	Carbohydrate transport and metabolism	InnerMembrane		344	344
arnT	Cell wall/membrane/envelope biogenesis	InnerMembrane		1643	1643
arnD	Carbohydrate transport and metabolism	Cytoplasmic		899	899
arnA	Translation, ribosomal structure and biogenesis	Cytoplasmic		1991	1991

ychF	Translation, ribosomal structure and biogenesis	Cytoplasmic		1091	1091
pth	Translation, ribosomal structure and biogenesis	Cytoplasmic		596	596
5'-nucleotidase/2'	Nucleotide transport and metabolism	Periplasmic		1991	1991
prs	Nucleotide transport and metabolism	Cytoplasmic		947	947
lolB	Cell wall/membrane/envelope biogenesis	Periplasmic		578	578
hemA	Coenzyme transport and metabolism	Cytoplasmic		1259	1259
prfA	Translation, ribosomal structure and biogenesis	Cytoplasmic		1088	1088
prmC	Translation, ribosomal structure and biogenesis	Cytoplasmic		848	848
Invasion gene expression up-regulator	Function unknown	InnerMembrane		383	392
Transglut_core2 domain-containing protein	Signal transduction mechanisms	Cytoplasmic		800	800
kdsA	Cell wall/membrane/envelope biogenesis	Cytoplasmic		854	854
dctA	Energy production and conversion	InnerMembrane		1280	1280
grcA	Coenzyme transport and metabolism	Cytoplasmic		380	380
ung	Replication, recombination and repair	Cytoplasmic		668	668
DUF3545 domain-containing protein	Unknown protein	Cytoplasmic		182	182
tal	Carbohydrate transport and metabolism	Cytoplasmic		950	962
Alanine:cation symporter family protein	Amino acid transport and metabolism	InnerMembrane		1442	1442
MoxR protein	General function prediction only	Cytoplasmic		920	920
cobW domain-containing protein	Posttranslational modification, protein turnover, chaperones	Cytoplasmic		650	650
group_140	Posttranslational modification, protein turnover, chaperones	InnerMembrane		638	788
ABC transmembrane type-1 domain-containing protein	Amino acid transport and metabolism	InnerMembrane		812	812
group_581	Amino acid transport and metabolism	InnerMembrane		1004	1004
group_582	Amino acid transport and metabolism	Periplasmic		1631	1631
hybD	Energy production and conversion	Cytoplasmic		530	530
Hydrogenase-2 large chain	Energy production and conversion	Periplasmic		1703	1703
Hydrogenase 2 b cytochrome subunit	Energy production and conversion	InnerMembrane		1187	1187
hydrogenase 2 operon protein HybA	Energy production and conversion	Periplasmic		1025	1025
Hydrogenase (acceptor)	Energy production and conversion	Cytoplasmic		1133	1133
amoG	Secondary metabolites biosynthesis, transport and catabolism	Cytoplasmic		2246	6293
amonabactin ABC transporter ATP-binding protein	Inorganic ion transport and metabolism	Cytoplasmic		803	803
amonabactin ABC transporter permease subunit 1	Inorganic ion transport and metabolism	InnerMembrane		1067	1067
amonabactin ABC transporter permease subunit 2	Inorganic ion transport and metabolism	InnerMembrane		1016	1016
siderophore amonabactin export MFS transporter	Carbohydrate transport and metabolism	InnerMembrane		1229	1229
Inner membrane protein YfeZ	Unknown protein	InnerMembrane		461	470
corA	Inorganic ion transport and metabolism	Cytoplasmic		947	947
Electron transfer flavoprotein-ubiquinone oxidoreductase	Energy production and conversion	Cytoplasmic		1643	1643

Electron transfer flavoprotein beta-subunit	Energy production and conversion	Cytoplasmic		749	749
ETF domain-containing protein	Energy production and conversion	Cytoplasmic		926	926
serC	Coenzyme transport and metabolism	Cytoplasmic		1088	1088
aroA	Amino acid transport and metabolism	Cytoplasmic		1283	1283
3-deoxy-D-arabino-heptulosonate 7-phosphate synthase	Amino acid transport and metabolism	Cytoplasmic		1055	1055
group_555	Signal transduction mechanisms	Cytoplasmic		404	1595
vgrG	Intracellular trafficking, secretion, and vesicular transport	Cytoplasmic		638	2231
type VI secretion system contractile sheath small subunit	Intracellular trafficking, secretion, and vesicular transport	Cytoplasmic		395	503
AHA_1833	Intracellular trafficking, secretion, and vesicular transport	Cytoplasmic		929	1478
AHA_1834	Intracellular trafficking, secretion, and vesicular transport	Cytoplasmic		431	431
AHA_1835	Intracellular trafficking, secretion, and vesicular transport	Cytoplasmic		1766	1766
AHA_1836	Intracellular trafficking, secretion, and vesicular transport	Cytoplasmic		998	998
AHA_1838	Intracellular trafficking, secretion, and vesicular transport	Cytoplasmic		515	515
AHA_1839	Intracellular trafficking, secretion, and vesicular transport	Cytoplasmic		1334	1334
AHA_1840	Intracellular trafficking, secretion, and vesicular transport	Cytoplasmic		779	779
Response regulator receiver domain	Signal transduction mechanisms	Cytoplasmic		971	971
YciI family protein	Secondary metabolites biosynthesis, transport and catabolism	Periplasmic		296	296
AHA_3493	Intracellular trafficking, secretion, and vesicular transport	InnerMembrane		635	635
Putative transport protein ASA_0825	General function prediction only	InnerMembrane		1661	1661
fkpA	Posttranslational modification, protein turnover, chaperones	Periplasmic		806	806
Protein SlyX homolog	Function unknown	Cytoplasmic		215	215
slyD	Posttranslational modification, protein turnover, chaperones	Cytoplasmic		635	638
DNA-binding protein	General function prediction only	Cytoplasmic		206	206
arnB	Cell wall/membrane/envelope biogenesis	Cytoplasmic		257	1136
group_1070	Unknown protein	Cytoplasmic		188	188
speA	Amino acid transport and metabolism	Cytoplasmic		1901	1901
pepT	Amino acid transport and metabolism	Cytoplasmic		1223	1223
group_1068	General function prediction only	Cytoplasmic		1724	1724
S-adenosyl-L-homocysteine hydrolase	Coenzyme transport and metabolism	Cytoplasmic		1121	1121
group_1067	Unknown protein	InnerMembrane		737	737
YceI domain-containing protein	General function prediction only	Periplasmic		560	560
group_6118	Transcription	Cytoplasmic		902	902
Biotin_lipoyl_2 domain-containing protein	Defense mechanisms	Cytoplasmic		1004	1004
tRNA-dihydrouridine synthase B	Translation, ribosomal structure and biogenesis	Cytoplasmic		860	965
fis	Transcription	Cytoplasmic		296	296
group_5699	Unknown protein	Cytoplasmic		263	263

group_622	Signal transduction mechanisms	Cytoplasmic		821	821
Glucitol operon repressor	Transcription	Cytoplasmic		797	797
Tagatose-bisphosphate aldolase subunit KbaZ	Carbohydrate transport and metabolism	Cytoplasmic		1313	1313
Putative tagatose-6-phosphate ketose/aldose isomerase	Cell wall/membrane/envelope biogenesis	Cytoplasmic		1169	1169
PTS N-acetylgalactosamine transporter subunit IIB	Carbohydrate transport and metabolism	Cytoplasmic		473	473
N-acetylgalactosamine permease IIC component 1	Carbohydrate transport and metabolism	InnerMembrane		776	776
N-acetylgalactosamine-specific enzyme IID component of PTS	Carbohydrate transport and metabolism	InnerMembrane		878	878
N-acetyl-galactosamine	Carbohydrate transport and metabolism	Cytoplasmic		434	434
